# Different metabolic responses to PI3K inhibition in NSCLC cells harboring wild-type and G12C mutant KRAS

**DOI:** 10.18632/oncotarget.9849

**Published:** 2016-06-06

**Authors:** Elisa Caiola, Laura Brunelli, Mirko Marabese, Massimo Broggini, Monica Lupi, Roberta Pastorelli

**Affiliations:** ^1^ Laboratory of Molecular Pharmacology, Department of Oncology, IRCCS-Istituto di Ricerche Farmacologiche “Mario Negri”, Milan, Italy; ^2^ Protein and Gene Biomarkers Unit, Laboratory of Mass Spectrometry, Department of Environmental Health Sciences, IRCCS-Istituto di Ricerche Farmacologiche “Mario Negri”, Milan, Italy; ^3^ Laboratory of Cancer Pharmacology, Department of Oncology, IRCCS-Istituto di Ricerche Farmacologiche “Mario Negri”, Milan, Italy

**Keywords:** KRAS, NSCLC, metabolomics, BEZ235, BKM120

## Abstract

KRAS mutations in non-small-cell lung cancer (NSCLC) patients are considered a negative predictive factor and indicate poor response to anticancer treatments. KRAS mutations lead to activation of the PI3K/akt/mTOR pathway, whose inhibition remains a challenging clinical target. Since the PI3K/akt/mTOR pathway and KRAS oncogene mutations all have roles in cancer cell metabolism, we investigated whether the activity of PI3K/akt/mTOR inhibitors (BEZ235 and BKM120) in cells harboring different KRAS status is related to their metabolic effect. Isogenic NSCLC cell clones expressing wild-type (WT) and mutated (G12C) KRAS were used to determine the response to BEZ235 and BKM120. Metabolomics analysis indicated the impairment of glutamine in KRAS-G12C and serine metabolism in KRAS-WT, after pharmacological blockade of the PI3K signaling, although the net effect on cell growth, cell cycle distribution and caspase activation was similar. PI3K inhibitors caused autophagy in KRAS-WT, but not in KRAS-G12C, where there was a striking decrease in ammonia production, probably a consequence of glutamine metabolism impairment.

These findings lay the grounds for more effective therapeutic combinations possibly distinguishing wild-type and mutated KRAS cancer cells in NSCLC, exploiting their different metabolic responses to PI3K/akt/mTOR inhibitors.

## INTRODUCTION

Lung cancer is the leading cause of cancer deaths worldwide and non-small cell lung cancer (NSCLC) accounts for 80% of all lung cancer cases [1]. Five years survival for advanced NSCLC is currently below 20%, highlighting the need for new treatment strategies [2, 3]. KRAS is one of the most frequently mutated oncogenes in NSCLC and 90% of these mutations affect codon 12, in which the glycine (G) can be replaced with aspartic acid (D), valine (V), or cysteine (C), the G12C substitution being the most frequent. These mutations lead to uncontrolled cell growth, proliferation and survival [4]. At preclinical level, the different substitutions of glycine at position 12 give different responses *in vitro* and *in vivo* to conventional chemotherapeutics [5, 6]. Although KRAS is one of the earliest known oncogenic drivers in NSCLC, effective targeting remains a therapeutic challenge. All attempts to target it directly have failed and KRAS is widely assumed to be undruggable [7]. Recently, a specific allosteric inhibitor of G12C mutated KRAS was described, showing promising preclinical results [8].

KRAS signaling is highly complex and dynamic, engaging various downstream effectors, such as canonical Raf/Mek/Erk and PI3K/akt/mTOR signaling networks [9, 10]. KRAS mutations lead to the activation of PI3Ks in lung tumor maintenance [11]. The PI3Ks are members of a conserved family of lipid kinases, grouped in three classes: I (the most studied in cancer), II and III according to their substrate preference and sequence homology [12]. Activation of PI3Ks leads to the activation of several proteins that can phosphorylate target proteins regulating many cellular functions. The main consequences of this activation cascade in cancer are cell survival, proliferation and growth [13, 14].

Several approaches are currently attempting to inhibit downstream molecules in the PI3K/akt/mTOR pathway to impair its activation [15]. A number of inhibitors are available for preclinical research such as BEZ235 (a dual PI3K/mTOR inhibitor) and BKM120 (a pan PI3K inhibitor). Although preclinically promising, these agents have shown only limited activity in early phase clinical trials and it is likely that cancer cells acquire resistance through different feedback loops and crosstalk mechanisms [16, 17]. Novel inhibitors of the PI3K/akt/mTOR pathway are under investigation, and their potential clinical utility may well be demonstrated soon. Nevertheless, the pivotal importance of PI3K signaling activation in cancer and the potential effectiveness of inhibitors shown at preclinical level, mean that we need a better comprehension of the mechanism by which these compounds inhibit cell growth, to help achieve better clinical responses.

In recent years, particular attention has been paid to the role of cellular metabolism not only in cancer cell growth, but also in the cellular response to treatment [18–20]. Considering the role of PI3K/akt/mTOR pathway in cell metabolic control [14, 21, 22] and knowing that KRAS-mutated NSCLC cells display a distinct metabolic profile [23], it is important to understand whether the activity of these inhibitors is related to their effect at metabolic level in cells with a different KRAS mutational status. This would lay the grounds for new therapeutic combinations, possibly distinguishing between wild-type (WT) and mutated cancer cells, to contribute to patient-tailored treatments.

We employed our robust isogenic system [5], and applied a targeted metabolomics strategy to profile the metabolic cellular responses after the inhibition of PI3K signaling in NSCLC clones harboring KRAS-G12C or -WT isoforms. Although there is ample knowledge of the specific mechanisms of action of BEZ235 and BKM120 on NSCLC [24–26], little is known about the metabolic responses to PI3K signaling impairment in NSCLC tumor cells with KRAS-G12C mutations, thus hampering the discovery of possible new metabolic targets with better drug responses.

## RESULTS

### BEZ235 and BKM120 inhibited cell growth in NSCLC cell lines harboring KRAS-G12C or KRAS-WT isoforms

**Figure 1:**
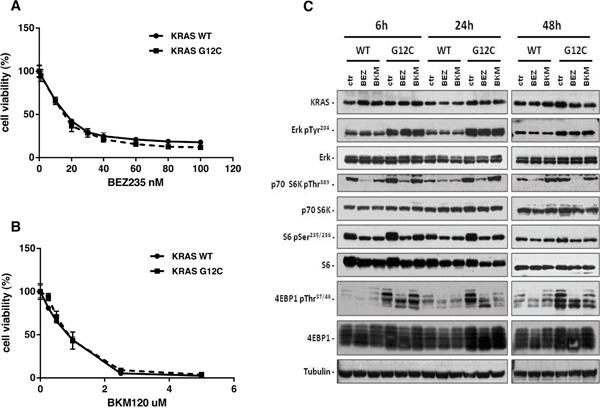
KRAS-G12C and KRAS-WT clone responses to BEZ235 and BKM120 treatments and PI3K pathway modulation *Panels* **A, B.** Responses of cells to BEZ235 (A) and BKM120 (B), detected by MTS assay. The average of three independent experiments and SD are shown. *Panel* **C.** Representative Western blot analysis reporting the expression of different proteins from the MAPK and PI3K pathways in the KRAS expressing clones treated with BEZ235 (25 nM) or BKM120 (1 μM) at 48h. Tubulin was used as loading control.

Using isogenic NCI-H1299 derived clones, previously characterized for their *in vitro* and *in vivo* growth, KRAS protein expression and activation levels [6, 23], we determined the *in vitro* growth inhibitory activity of BEZ235, a dual PI3K/mTOR inhibitor (Figure 1A) and BKM120, a pan PI3K inhibitor (Figure 1B). Different KRAS status, KRAS-G12C or KRAS-WT, did not cause distinct sensitivity patterns towards the two drugs detected by MTS assay after 72h of treatment. The calculated IC50 values for BEZ235 were 15.6 nM and 13.1 nM, and respectively 0.7 μM and 0.84 μM for BKM120 in the KRAS-G12C or KRAS-WT expressing clones.

To check whether the presence of WT or mutated KRAS induced different downstream signaling, we evaluated the activation of PI3K and MAPK pathways at different times after BEZ235 (25 nM) or BKM120 (1 μM). For this experiment we applied a drug concentration able to kill 60% of cells (IC60) at 72h, therefore inducing pathway modifications also at early time points.

As shown in Figure 1C, BKM120 induced a transient decrease of p-p70S6K around 6-24h post-treatment in the KRAS-WT clone, while no effect was detected for KRAS-G12C. Expression of p-p70S6K was reduced at all times for both WT and G12C clones after BEZ235 treatment. The pattern of p-S6 was similar in all clones with both drugs: activation of S6 was lower 6 and 24h after the start of treatment and comparable to the untreated control after 48h. Analysis of p-4EBP1 did not indicate any clear BKM120-induced changes in the different clones. However, BEZ235 slightly reduced protein phosphorylation in both WT and G12C. In the MAPK pathway, neither drugs modified the activation of Erk at any time although, as expected, basal p-Erk in KRAS-G12C was higher than KRAS-WT.

### Different KRAS isoforms displayed distinct metabolic responses to PI3K inhibitor treatments

To investigate the metabolic effects of PI3K inhibitors in NCI-H1299 cells overexpressing KRAS-G12C or KRAS-WT isoforms (three biological replicates/condition), we applied a targeted quantitative metabolomics analysis at 6, 24 and 48h post-treatment.

Our strategy allowed to unambiguously identify and quantify 186 metabolites including lipids, aminoacids, biogenic amines and acylcarnitines. To ensure data quality and robust statistical analysis the following filtering criteria were applied: (i) metabolites measured with more than 20% missing data (no detectable peak) were excluded for any further data elaboration; (ii) metabolites for which the sample concentration was below the limit of detection (<LOD) in at least ≥ 50% of analyzed samples were excluded. In total, 150 metabolites were selected after this quality control: 1 hexose, 21 amino acids, 13 biogenic amines, 13 acylcarnitines, 15 sphingomyelins, 87 glycerophospholipids. Analytical details and specifications are reported in Supplementary Information. Concentrations of all analyzed metabolites for each sample are expressed as μM and shown in Supplementary Table S1.

To identify the significant metabolites involved in the responses to PI3K inhibitors, we used a multivariate statistical approach (OPLS-DA). We selected a panel of deregulated metabolites (s-plot) to significantly distinguish treated KRAS-G12C or KRAS-WT clones from their untreated counterparts for each time (Supplementary Table S2).

**Figure 2:**
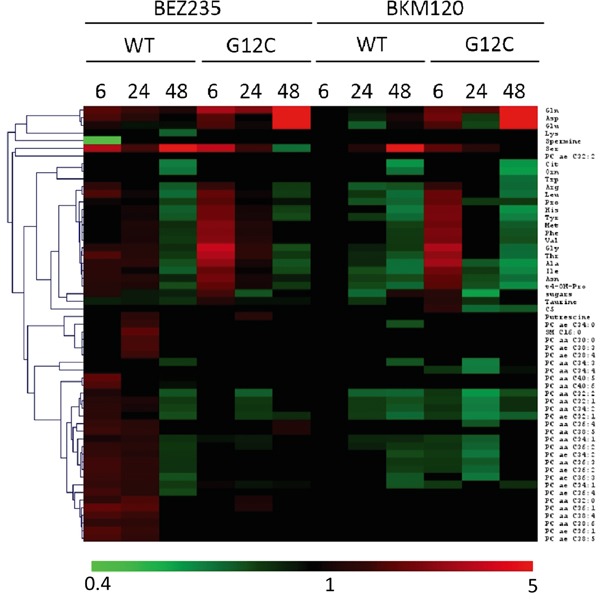
Metabolic responses to PI3K inhibition in NSCLC harboring KRAS-G12C or KRAS-WT isoforms Heat map and hierarchical clustering of discriminant metabolites (OPLS-DA, s-plot) in KRAS-G12C and KRAS-WT NSCLC cell clones treated with BEZ235 (25 nM) or BKM120 (1 μM) for 6, 24, 48h. Each row represents a metabolite, each column the average metabolite concentration (three biological replicates) for each experimental condition.

The PI3K inhibitors treatment caused substantial metabolic changes, dependent on time, treatment and KRAS mutational status in NCI-H1299 cells overexpressing KRAS-G12C or -WT isoforms (Figure 2). BEZ235 induced metabolic changes in KRAS-WT as well as in KRAS-G12C, already observable 6h after the treatment and continuing for all the time points. BEZ235 affected fewer metabolites in KRAS-G12C mutant than in KRAS-WT. BKM120 induced more metabolic alterations in the KRAS-G12C mutant clone starting from 6h and for all the subsequent time points, while in KRAS-WT metabolite levels changed only after 24h of treatment (Table 1).

**Table 1:** Numbers of metabolites distinguishing treated KRAS-WT or KRAS-G12C clones from their untreated counterparts for each time point, using multivariate analysis OPLS-DA, s-plot

	Number of Metabolites					
	6h		24h		48h	
	WT	G12C	WT	G12C	WT	G12C
BEZ235 (25 nM)	38	23	42	28	37	20
BKM120 (1 μM)	0	32	22	25	34	27

**Figure 3:**
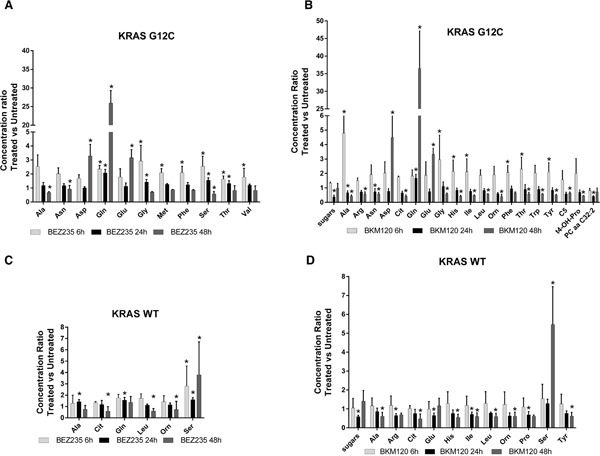
Significantly deregulated metabolites in presence of KRAS isoforms after PI3K signaling impairment *Panels* **A, B.** Significant (one-way ANOVA, Tukey-Kramer HSD) metabolite concentration ratio (treated vs untreated condition) in NSCLC harboring KRAS-G12C mutation, at 6, 24, 48h after 25 nM BEZ235 (A) or 1 μM BKM120 (B). *Panels* **C, D.** Significant (one-way ANOVA, Tukey-Kramer HSD) metabolite concentration ratio (treated vs untreated condition) in NSCLC harboring KRAS-WT, at 6, 24, 48h after 25 nM BEZ235 (C) or 1 μM BKM120 (D).

When we focused on metabolites whose abundances differed significantly (p<0.05, ANOVA Tukey-Kramer HSD) between treated and untreated KRAS isoforms, we found that PI3K inhibitors mainly altered amino acids. Overall, amino acids concentration tended to decrease after 48h of treatment with BEZ235 and BKM120 in both KRAS-WT and -G12C clones, with the exception of glutamine (Gln), aspartic acid (Asp) and glutamate (Glu), whose levels rose strongly only in the mutant (Figure 3A, 3B). Serine (Ser) showed a steep increase in concentration 48h after BEZ235 and BKM120 in KRAS-WT (Figure 3C, 3D) but not in the mutant, where its level was reduced only by BEZ235 (Figure 3A). Gln, Asp and Glu are intermediates of the glutaminolysis to fuel the TCA cycle, raising the possibility that the PI3K signaling impairment in the KRAS-G12C mutant might interfere with this primary source of energy of this cell clone. Indeed, as we reported recently [23], only the NSCLC harboring KRAS-G12C mutation was addicted to Gln to sustain its growth and proliferation. Similar significant (p<0.05, ANOVA Tukey-Kramer HSD) decreases in the uptake of Gln and Ser were observed in KRAS-G12C and KRAS-WT conditioned culture medium after 48h of treatment (Supplementary Figure S1A). Neither PI3K inhibitor changed hexose consumption (Supplementary Figure S1B).

To identify how PI3K inhibitors affected the glutamine metabolism, we treated KRAS-G12C and KRAS-WT clones with BEZ235 and BKM120 to test the gene expression of the key enzymes involved in the glutaminolysis pathway (Supplementary Figure S2A) and c-MYC, which regulates glutamine uptake and metabolism [27]. We found no significant differences in the expression levels of these proteins (Supplementary Figure S2B, S2C).

### PI3K signaling impairment affected autophagy in NSCLC KRAS-G12C clone only

**Figure 4:**
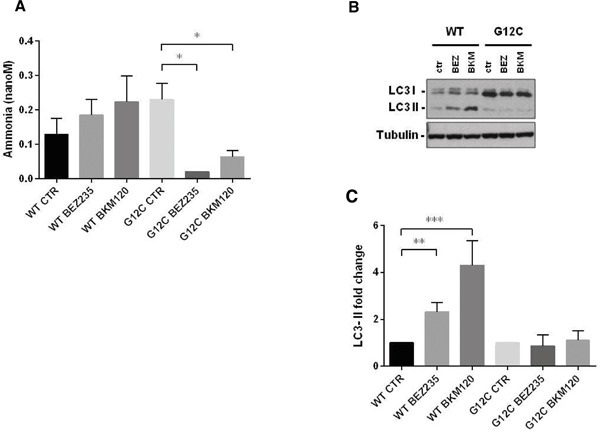
Ammonia release and autophagy in presence of KRAS isoforms after PI3K inhibitors *Panel* **A.** Ammonia release in conditioned medium of NSCLC cell clones harboring KRAS-G12C or KRAS-WT isoforms 48h after BEZ235 (25 nM) or BKM120 (1 μM) treatment. Data are the mean ± SD of three independent experiments. *P < 0.05, one-way ANOVA, Tukey-Kramer HSD. *Panel* **B.** Representative Western blot analysis reporting the expression of LC3 forms in the KRAS clones at 48 h after BEZ235 (25 nM) or BKM120 (1 μM) treatment. *Panel* **C**. Band intensities of LC3-II were quantified and individually normalized to Tubulin band intensities. Data are expressed as LC3-II expression fold change of treated clones vs controls, which where arbitrarily set to 1. Histograms represent mean ± SD of three independent experiments. **P <0.01, *** P <0.001, one-way ANOVA, Tukey-Kramer HSD.

A direct link has been reported between the Gln metabolism and autophagic activity, mainly through the ammonia produced by the deamination of Gln (Supplementary Figure S2A) [28, 29]. We measured ammonia released in growth medium in our KRAS-G12C and KRAS-WT clones after 48h of BEZ235 or BKM120 treatment. Release was significantly reduced only in the KRAS mutant. In the KRAS-WT clone BEZ235 and BKM120 did not significantly alter the ammonia level (Figure 4A).

To link differences in ammonia release to differences in autophagic induction, we investigated the level of the autophagosomal marker LC3 in KRAS-WT and KRAS-G12C cell clones 48h after P13K inhibitor treatment. As shown in Figure 4B and 4C, both PI3K inhibitors induced an increase in LC3-II protein in KRAS-WT (suggestive of autophagic activity) but not in the KRAS-G12C mutant clone. To note, the KRAS-WT showed an accumulation of LC3-II also at 6 and 24h post-treatment (data not shown). The data obtained were confirmed using an independent assay (Cyto-ID) measuring stained autophagic compartments either in untreated and PI3K inhibitors-treated cells (Supplementary Figure S5).

PI3K inhibitors were able to induce authophagy only in KRAS-WT clone.

### PI3Ks inhibitors, at doses relevant for autophagy, did not alter apoptosis and cell cycle phases distribution in KRAS-WT and KRAS-G12C clones

**Figure 5:**
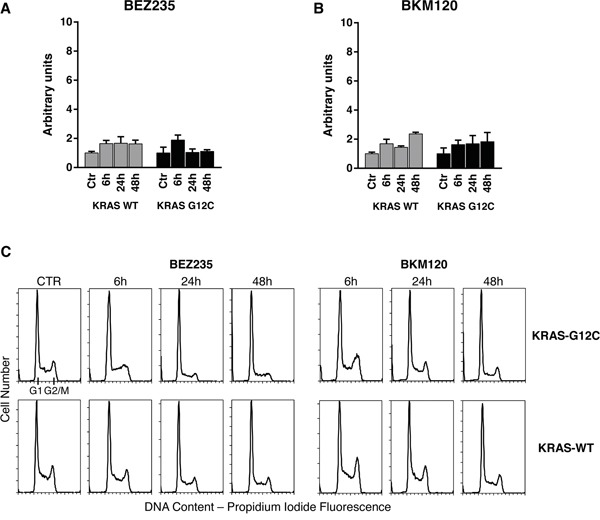
Apoptosis and cell cycle phase distribution in KRAS-G12C and KRAS-WT cells treated with PI3K inhibitors *Panel* **A, B.** Caspases 3 and 7 activities in clones treated with 25 nM BEZ235 (A) and 1 μM BKM120 (B) at the times indicated. The averages of three different biological replicates and SD are shown. No significant differences were observed (ANOVA, Tukey-Kramer HSD). *Panel* **C.** Cell cycle phase distribution in KRAS-G12C and KRAS-WT cells treated with 25 nM BEZ235 and 1 μM BKM120.

Since autophagy and apoptosis can be triggered by common upstream signals, and sometimes this results in combined induction of autophagy and apoptosis, we investigated the activation of caspase 3/7 in our system after treatment. Neither of the drugs at the doses used for autophagy evaluation had any relevant effect on the central step of apoptosis in the two KRAS clones (Figure 5A and 5B). When the doses of PI3Ks inhibitors were doubled (BEZ235 50 nM, BKM120 2 μM), caspase 3/7 was activated only in the KRAS-WT clone after BKM120 (Supplementary Figure S3).

To further clarify the behavior of clones, cell cycle perturbation was evaluated by flow cytometric analysis at different times after BEZ235 or BKM120. Cell cycle distribution after the PI3K inhibitors at low doses (25 nM BEZ235, 1 μM BKM120) was similar for both KRAS isoforms. BEZ235 induced a partial accumulation of cells in G1 phase starting at 6h and up to 48h of treatment, while a small amount of cells treated with BKM120 was intercepted by the G2/M checkpoint 6h after the start of treatment (Figure 5C). The percentages of cells in cell cycle phase analysis are reported in Supplementary Table S3. This pattern was confirmed at higher doses (50 nM BEZ235, 2 μM BKM120) (Supplementary Figure S4 and Supplementary Table S4), where accumulation in G1 and G2/M became evident. At 24h and 48h, clones treated with BKM120 showed a reduction of the G2/M peak and an increasing sub-G1 peak, more marked in KRAS-WT than KRAS-G12C (Supplementary Figure S4), in agreement with the observed caspase activity (Supplementary Figure S3).

## DISCUSSION

KRAS mutations are genetic events that occur early in tumor progression and are associated with more aggressive tumor phenotypes and/or resistance to treatment. KRAS mutations lead to the activation of several signaling pathways including PI3K/akt/mTOR [11]. In NSCLC, roughly one third of the patients present mutation in the KRAS oncogene, with G12C substitution being the most frequent and constituting a negative predictive factor for response to first-line cisplatin therapy, as recently reported by our group [30].

Here we show that the dual PI3K/mTOR inhibitor BEZ235 and the pan PI3K inhibitor BKM120 have similar activity in terms of cell growth inhibition in KRAS-WT and KRAS-G12C mutated clones. In view of this similar growth inhibition, and since they induce a comparable block in the PI3K signaling pathway, we wondered whether this final effect was reached through different mechanisms in KRAS-WT and -G12C cells, considering their different basal metabolic profiles recently reported by our group [23]. This is important since, despite the strong efficacy of these inhibitors in pharmacological modulation of the PI3K/akt/mTOR pathway at the preclinical level, their therapeutic clinical efficacy has been far below expectancy. This has been ascribed to several factors: lack of specificity, feedback loops and crosstalk mechanisms and adaptation of the cellular metabolism [31–33].

More is known about the mechanism of action of PI3K inhibitors but nothing about the metabolic rewiring they induce on NSCLC harboring different KRAS isoforms. Identification of mutant KRAS specific metabolic alterations in response to treatment would open up the possibility of combining these drugs with specific agents on metabolism to maximize their anticancer activity. We found that the presence of different KRAS isoforms in NSCLC cell clones did indeed induce different metabolic responses after pharmacological impairment of the PI3K signaling. In particular, the KRAS-G12C mutant clone presented alterations of the glutamine metabolism supported by the accumulation of glutamine, glutamate and aspartic acid, all utilized to generate α-ketoglutarate and ammonia to sustain cell proliferation [34]. We have already reported that the KRAS-G12C clone was addicted to glutamine to grow and proliferate [23], so the accumulation of the two main players in the glutaminolysis pathway (glutamine and glutamate) indicates that the PI3K inhibitors interfered with the principal energy source for this cancer cell clone. This effect of the PI3K inhibitors was not ascribable to changes in the expression levels of the key enzymes in the glutaminolysis pathway, nor to any alteration of the expression of the c-MYC gene, which regulates glutamine uptake and metabolism [27]. Further studies will be necessary to explore the role of other pathways to link the PI3K signaling to glutaminolysis in the KRAS mutant clone.

In contrast, the KRAS-WT clone did not show any significant accumulation of metabolites involved in the glutamine metabolism after BEZ235 and BKM120 treatment, again supporting our previous evidence that the KRAS-G12C mutation affects the cell responses [5, 6, 23]. Striking accumulation of serine after PI3K inhibition was only seen in the KRAS-WT clone. Serine is essential in several cellular processes such as synthesis of nucleotides, proteins and lipids required for proliferation. In anabolic pathways, the serine biosynthetic pathway is a crucial turning point in glucose conversion, acting on glycolysis [35–37]. Therefore, serine accumulation after PI3K inhibition suggests KRAS-WT is unable to use this aminoacid properly with the consequent energy unbalance that might abrogate NSCLC proliferation capacity [38].

The impairment of glutamine and serine metabolism respectively in KRAS-G12C and KRAS-WT clones, was also confirmed by the significantly lower uptake of glutamine and serine in their conditioned media. These altered metabolic pathways represent attractive therapeutic targets. For instance agents able to interfere with central metabolic pathways already exist and some have been shown to be effective in preclinical cancer models [39–42]. Despite many examples of promising metabolic targets for cancer therapy, few studies have addressed the issue in the frame of NSCLC therapeutic clinical efficacy [43, 44]. Attacking metabolism as a downstream consequence of the KRAS mutational status would be synergistic with the classical therapy and might result in improved therapeutic response of NSCLC. Moreover targeting metabolic Achilles' heels specific to KRAS isoforms will help in determining if a sufficient therapeutic window exists to spare normal cells and effectively target cancer cells. Such targeting strategy might pave the way to potential new therapeutic possibilities in NSCLC current chemotherapy. To note that despite the efficacy of PI3K/akt/mTOR inhibitors at preclinical level, their therapeutic clinical efficacy has been well below the expectancies. Metabolic stress produced by the loss of a single nutrient input (e.g. glutamine, serine) can activate autophagy [45]. This is a process the cell activates to mitigate damage and provide nutrients for short-term survival and mTOR is important in this process. Ammonia generated during glutamine metabolism induces autophagy and autophagic removal of toxic by-products. Ammonia-induced authophagy is now thought to occur independently from mTOR [29]. We observed that PI3K inhibitors induced autophagy, redistributing LC3 markers, only in KRAS-WT cells, in which the ammonia level was preserved, whereas they did not in the KRAS-G12C clone, where the striking drop in ammonia production is plausibly the consequence of glutamine metabolism impairment.

**Figure 6:**
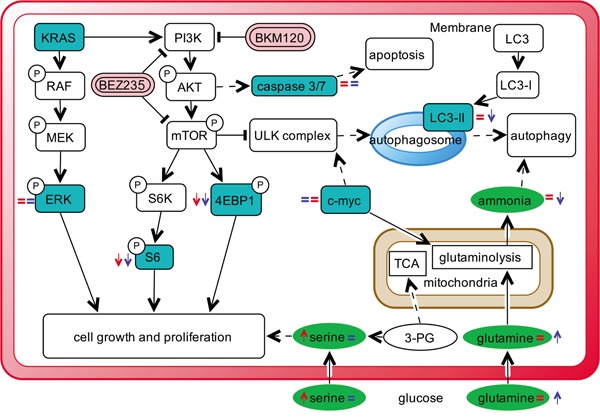
Schematic overview summing up the effects of PI3K inhibitors in NSCLC harboring G12C or WT KRAS isoforms observed in this study Schematic diagram depicting the most representative KRAS/PI3K/AKT/mTOR signaling pathway and its connection to cellular metabolism. Dashed lines refers to complex connections not detailed for clarity. Colored boxes refer to metabolites (circle)/proteins (rectangle) measured in this study. Arrows indicate metabolite/protein whose level is increased/decreased after PI3K inhibitor treatment compared to untreated conditions in G12C (red) and WT (blue) KRAS clones compare to untreated conditions. Equal (=) sign indicates metabolite/protein whose level does not change after PI3K inhibitor treatment in G12C (red) and WT (blue) KRAS clones compare to untreated conditions.

This again indicates the presence of different metabolic responses induced by KRAS-G12C and KRAS-WT in NSCLC cells after pharmacological impairment of PI3K signaling, although the net effect on cell growth, cell cycle distribution and caspase activation are similar. It is likely that KRAS-WT cells activate ammonia-induced autophagy after drug treatment probably to cope with energy imbalance stress (derangement in serine metabolism), whereas the KRAS-G12C cells do not display this compensatory mechanism triggered by ammonia because of their glutamine metabolism impairment. Figure 6 visually summarizes our main results in relation to the PI3K/akt/mTOR pathways, along with the used inhibitors and cell metabolism.

In conclusion, these findings may have important implications in NSCLC through the development of combination strategies (e.g. nutrient modification) to increase the killing of cancer cells expressing mutant KRAS, enhancing the selectivity and finally improving the therapeutic index.

## MATERIALS AND METHODS

### Cell cultures and drugs

The NCI-H1299 derived clones were grown in RPMI-1640 medium including 500 μg/ml of G418 (Gibco). Clones were obtained by transfecting the NCI-H1299 cell line with the expression plasmids encoding for the mutant KRAS-G12C and KRAS-WT as a control. Details of transfection, KRAS protein expression and activation are reported in our previous works [5, 6] and in Supplementary Information.

Cells are routinely tested for mycoplasma contamination by PCR and authenticated with the PowerPlex 16 HS System (Promega) every six months by comparing the STR profiles with those deposited in ATCC and/or DSMZ databases. All drugs were dissolved in medium just before use. Treatments were performed with BEZ235 at 25 nM or with BKM120 at 1 μM for 6h, 24h and 48h unless otherwise specified. The MTS assays (Promega) were done as described in [6]. Survival curves were plotted as percentages of untreated controls, consisting in at least six replicates for each time point, and show the mean and SD of at least three independent experiments.

### Metabolomic samples

NCI-H1299 cell lines harboring KRAS-G12C or KRAS-WT isoforms treated with BEZ235 (25 nM) or BKM (1 μM) and their untreated counterparts were collected after 6, 24 and 48h of drug treatment. Metabolites were extracted as reported [23]. Briefly, NSCLC cell, of each clone and experimental condition (three biological replicates/clone/condition) were rapidly rinsed in saline solution (~ 2s), aspirated, and metabolism was quenched by adding ~15 mL of liquid N2 to the dish. The plates were then stored at −80°C, and extracted and analyzed within seven days. Extraction was done by adding 1 mL of ice-cold MeOH to each plate and cells were scraped. Extracts were transferred to 1.5 mL micro-centrifuge tubes and pelleted at 4°C for 15 min at 10000xg.

Conditioned cell culture medium was collected 48 h after inhibitors treatment, 1mL of each culture medium was filtered (0.2 μm; Corning, Wiesbaden, Germany) and thirty microliters of filtered medium were used for metabolomic analysis.

### Absolute metabolite profiling

A targeted quantitative approach using a combined direct flow injection and liquid chromatography (LC) tandem mass spectrometry (MS/MS) assay (AbsoluteIDQ 180 kit, Biocrates) was applied for the metabolomics analysis. The method combines derivatization and extraction of analytes with the selective mass-spectrometric detection using multiple reaction monitoring (MRM) pairs. Isotope-labeled internal standards are integrated into the platform for metabolite absolute quantification. This targeted strategy allows for simultaneous detection and quantification of up to 186 metabolites from five analyte groups: acylcarnitines, amino acids, biogenic amines, hexoses (sum of hexoses), phosphatidylcholines (PCs), and sphingomyelins (SMs) (Supplementary Table S5). Samples were analyzed (30 microL) using an LC/MS (Triple quad 5500; AB Sciex) method (for analysis of amino acids and biogenic amines) followed by FIA-MS (analysis of lipids, acylcarnitines and hexose). Analytical details and specifications are reported in Supplementary Information.

The method of AbsoluteIDQ p180 kit has been proven to be in conformance with FDA Guideline Guidance for Industry—Bioanalytical Method Validation (May 2001), which implies proof of reproducibility within a given error range. Data evaluation for quantification of metabolite concentrations and quality assessment have been performed with the MetIDQ software package, which is an integral part of the AbsoluteIDQ kit. The metabolite concentration of each metabolite in each experimental condition were compared with the measurement detection limit specifications as reported by the manufacturer of the AbsoluteIDQ p180 kit (Biocrates). A metabolite was excluded from further analyses if its concentration measurement data did not meet all of the following criteria: (1) less than 20% of missing values (non-detectable peak) for each quantified metabolite in each experimental group; (2) 50% of all sample concentrations for the metabolite had to be above the limit of detection (LOD).

Metabolite data expressed as micromolar concentration (μM), from each experimental condition (cell clones/treatments), were examined using the SIMCA-P13 software package (Umetrics) for multivariate analysis. The variables were scaled using Pareto. To maximize class discrimination, the data were analyzed by orthogonal partial least-squares discriminant analysis (OPLS-DA). S-plots were calculated to visualize the relationship between covariance and correlation within the OPLS-DA results. Heat map and Hierarchical clustering were done using the MeV module of the TM4 package (http://www.TM4.org).

One-way ANOVA and multiple comparisons test were done on OPLS-DA discriminant metabolites (Tukey-Kramer HSD, Prism v. 6.05; GraphPad Software Inc) to identify metabolites differing significantly between treated and untreated KRAS isoforms.

### Real-time PCR

Total RNA was reverse-transcribed with a High-Capacity cDNA Kit (Life Technologies) and amplified by 7900HT Sequence Detection System (Life Technologies). Actin was used as internal control. Primers and TaqMan probes were purchased for all genes as ready-to-use solutions (Life Technologies). Two samples that showed at least two-fold differences were considered differently expressed.

### Western blotting analysis

Proteins were extracted and visualized as reported in [30]. Immunoblotting was carried out with the following antibodies: anti-p70S6K(Thr389) #9206, anti-p70S6K #9202, anti-S6(Ser235/236) ribosomal protein #2211, anti-S6 ribosomal protein 2217#, anti-4E-BP1(Thr37/46) #2855, anti-4E-BP1 #9644 provided by Cell Signalling Technology. Anti-Erk #sc94, anti-Erk(Tyr204) #sc7383, anti-KRAS #sc30 and anti-tubulin#9104 were obtained from Santa Cruz Biotechnology. Anti-LC3 #PM036 was obtained from MBL.

### Caspases 3 and 7 activity

Twenty-four hours after cell plating, BEZ235 (25 and 50 nM) or BKM (1 and 2 μM) was added. Twenty-four or 48h later, caspase activity was assessed using the Caspase-Glo 3/7 Assay (Promega) according to the manufacturer's instructions.

### Cell cycle analysis

Sample preparation and monoparametric DNA histograms analysis were done in untreated or treated BEZ235 (25 nM) or BKM (1 μM) as described in [6].

### Ammonia levels

Ammonia levels were measured in cell-conditioned medium in NSCLC harboring KRAS-G12C or KRAS-WT isoforms 48 hours after BEZ235 (25 nM) or BKM120 (1 μM) treatment, following the manufacturer's instruction (Ammonia assay kit, Abcam).

### Statistical analyses

Statistical analyses were done using GraphpadPrism version 6.05. Tests to analyze specific experiments are indicated in the legends to the figures. Differences between groups were considered statistically significant when the p-values were ≤0.05.

## SUPPLEMENTARY FIGURES AND TABLES







## References

[1] Jemal A, Siegel R, Xu J, Ward E (2010). Cancer statistics, 2010. CA Cancer J Clin.

[2] Oser MG, Niederst MJ, Sequist LV, Engelman JA (2015). Transformation from non-small-cell lung cancer to small-cell lung cancer: molecular drivers and cells of origin. Lancet Oncol.

[3] Piva S, Ganzinelli M, Garassino MC, Caiola E, Farina G, Broggini M, Marabese M (2014). Across the universe of K-RAS mutations in non-small-cell-lung cancer. Curr Pharm Des.

[4] Schubbert S, Shannon K, Bollag G (2007). Hyperactive Ras in developmental disorders and cancer. Nat Rev Cancer.

[5] Garassino MC, Marabese M, Rusconi P, Rulli E, Martelli O, Farina G, Scanni A, Broggini M (2011). Different types of K-Ras mutations could affect drug sensitivity and tumour behaviour in non-small-cell lung cancer. Ann Oncol.

[6] Caiola E, Salles D, Frapolli R, Lupi M, Rotella G, Ronchi A, Garassino MC, Mattschas N, Colavecchio S, Broggini M, Wiesmuller L, Marabese M (2015). Base excision repair-mediated resistance to cisplatin in KRAS(G12C) mutant NSCLC cells. Oncotarget.

[7] Cox AD, Fesik SW, Kimmelman AC, Luo J, Der CJ (2014). Drugging the undruggable RAS: Mission possible?. Nat Rev Drug Discov.

[8] Ostrem JM, Peters U, Sos ML, Wells JA, Shokat KM (2013). K-Ras(G12C) inhibitors allosterically control GTP affinity and effector interactions. Nature.

[9] Pylayeva-Gupta Y, Grabocka E, Bar-Sagi D (2011). RAS oncogenes: weaving a tumorigenic web. Nat Rev Cancer.

[10] Castellano E, Downward J (2011). RAS Interaction with PI3K: More Than Just Another Effector Pathway. Genes Cancer.

[11] Castellano E, Sheridan C, Thin MZ, Nye E, Spencer-Dene B, Diefenbacher ME, Moore C, Kumar MS, Murillo MM, Gronroos E, Lassailly F, Stamp G, Downward J (2013). Requirement for interaction of PI3-kinase p110alpha with RAS in lung tumor maintenance. Cancer Cell.

[12] Vanhaesebroeck B, Vogt PK, Rommel C (2010). PI3K: from the bench to the clinic and back. Curr Top Microbiol Immunol.

[13] Davis WJ, Lehmann PZ, Li W (2015). Nuclear PI3K signaling in cell growth and tumorigenesis. Front Cell Dev Biol.

[14] Courtnay R, Ngo DC, Malik N, Ververis K, Tortorella SM, Karagiannis TC (2015). Cancer metabolism and the Warburg effect: the role of HIF-1 and PI3K. Mol Biol Rep.

[15] Chan BA, Hughes BG (2015). Targeted therapy for non-small cell lung cancer: current standards and the promise of the future. Transl Lung Cancer Res.

[16] Hennessy BT, Smith DL, Ram PT, Lu Y, Mills GB (2005). Exploiting the PI3K/AKT pathway for cancer drug discovery. Nat Rev Drug Discov.

[17] Caino MC, Ghosh JC, Chae YC, Vaira V, Rivadeneira DB, Faversani A, Rampini P, Kossenkov AV, Aird KM, Zhang R, Webster MR, Weeraratna AT, Bosari S (2015). PI3K therapy reprograms mitochondrial trafficking to fuel tumor cell invasion. Proc Natl Acad Sci U S A.

[18] Ghosh JC, Siegelin MD, Vaira V, Faversani A, Tavecchio M, Chae YC, Lisanti S, Rampini P, Giroda M, Caino MC, Seo JH, Kossenkov AV, Michalek RD (2015). Adaptive mitochondrial reprogramming and resistance to PI3K therapy. J Natl Cancer Inst.

[19] Serizawa M, Kusuhara M, Zangiacomi V, Urakami K, Watanabe M, Takahashi T, Yamaguchi K, Yamamoto N, Koh Y (2014). Identification of metabolic signatures associated with erlotinib resistance of non-small cell lung cancer cells. Anticancer Res.

[20] Sun Y, Daemen A, Hatzivassiliou G, Arnott D, Wilson C, Zhuang G, Gao M, Liu P, Boudreau A, Johnson L, Settleman J (2014). Metabolic and transcriptional profiling reveals pyruvate dehydrogenase kinase 4 as a mediator of epithelial-mesenchymal transition and drug resistance in tumor cells. Cancer Metab.

[21] Howell JJ, Manning BD (2011). mTOR couples cellular nutrient sensing to organismal metabolic homeostasis. Trends Endocrinol Metab.

[22] Dibble CC, Cantley LC (2015). Regulation of mTORC1 by PI3K signaling. Trends Cell Biol.

[23] Brunelli L, Caiola E, Marabese M, Broggini M, Pastorelli R (2014). Capturing the metabolomic diversity of KRAS mutants in non-small-cell lung cancer cells. Oncotarget.

[24] Xu CX, Zhao L, Yue P, Fang G, Tao H, Owonikoko TK, Ramalingam SS, Khuri FR, Sun SY (2011). Augmentation of NVP-BEZ235's anticancer activity against human lung cancer cells by blockage of autophagy. Cancer Biol Ther.

[25] Qu Y, Wu X, Yin Y, Yang Y, Ma D, Li H (2014). Antitumor activity of selective MEK1/2 inhibitor AZD6244 in combination with PI3K/mTOR inhibitor BEZ235 in gefitinib-resistant NSCLC xenograft models. J Exp Clin Cancer Res.

[26] Zito CR, Jilaveanu LB, Anagnostou V, Rimm D, Bepler G, Maira SM, Hackl W, Camp R, Kluger HM, Chao HH (2012). Multi-level targeting of the phosphatidylinositol-3-kinase pathway in non-small cell lung cancer cells. PLoS One.

[27] Wise DR, DeBerardinis RJ, Mancuso A, Sayed N, Zhang XY, Pfeiffer HK, Nissim I, Daikhin E, Yudkoff M, McMahon SB, Thompson CB (2008). Myc regulates a transcriptional program that stimulates mitochondrial glutaminolysis and leads to glutamine addiction. Proc Natl Acad Sci U S A.

[28] Eng CH, Abraham RT (2010). Glutaminolysis yields a metabolic by-product that stimulates autophagy. Autophagy.

[29] Polletta L, Vernucci E, Carnevale I, Arcangeli T, Rotili D, Palmerio S, Steegborn C, Nowak T, Schutkowski M, Pellegrini L, Sansone L, Villanova L, Runci A (2015). SIRT5 regulation of ammonia-induced autophagy and mitophagy. Autophagy.

[30] Marabese M, Marchini S, Sabatino MA, Polato F, Vikhanskaya F, Marrazzo E, Riccardi E, Scanziani E, Broggini M (2005). Effects of inducible overexpression of DNp73alpha on cancer cell growth and response to treatment in vitro and in vivo. Cell Death Differ.

[31] Costa C, Ebi H, Martini M, Beausoleil SA, Faber AC, Jakubik CT, Huang A, Wang Y, Nishtala M, Hall B, Rikova K, Zhao J, Hirsch E (2015). Measurement of PIP3 levels reveals an unexpected role for p110beta in early adaptive responses to p110alpha-specific inhibitors in luminal breast cancer. Cancer Cell.

[32] Juric D, Castel P, Griffith M, Griffith OL, Won HH, Ellis H, Ebbesen SH, Ainscough BJ, Ramu A, Iyer G, Shah RH, Huynh T, Mino-Kenudson M (2015). Convergent loss of PTEN leads to clinical resistance to a PI(3)Kalpha inhibitor. Nature.

[33] Locasale JW (2012). Metabolic rewiring drives resistance to targeted cancer therapy. Mol Syst Biol.

[34] Meng M, Chen S, Lao T, Liang D, Sang N (2010). Nitrogen anabolism underlies the importance of glutaminolysis in proliferating cells. Cell Cycle.

[35] Kalhan SC, Hanson RW (2012). Resurgence of serine: an often neglected but indispensable amino Acid. J Biol Chem.

[36] Gravel SP, Hulea L, Toban N, Birman E, Blouin MJ, Zakikhani M, Zhao Y, Topisirovic I, St-Pierre J, Pollak M (2014). Serine deprivation enhances antineoplastic activity of biguanides. Cancer Res.

[37] Maddocks OD, Berkers CR, Mason SM, Zheng L, Blyth K, Gottlieb E, Vousden KH (2013). Serine starvation induces stress and p53-dependent metabolic remodelling in cancer cells. Nature.

[38] Amelio I, Cutruzzola F, Antonov A, Agostini M, Melino G (2014). Serine and glycine metabolism in cancer. Trends Biochem Sci.

[39] Altman BJ, Rathmell JC (2012). Metabolic stress in autophagy and cell death pathways. Cold Spring Harb Perspect Biol.

[40] Marabese M, Marchini S, Sabatino MA, Polato F, Vikhanskaya F, Marrazzo E, Riccardi E, Scanziani E, Broggini M (2005). Effects of inducible overexpression of DNp73alpha on cancer cell growth and response to treatment in vitro and in vivo. Cell Death Differ.

