# Supplementary material for: Different metabolic responses to PI3K inhibition in NSCLC cells harboring wild-type and G12C mutant KRAS
Source: Oncotarget. 2016 Jun 6;7(32):51462–72. doi: 10.18632/oncotarget.9849 (PMC5239488; doi:10.18632/oncotarget.9849)
Supplement: Supplementary file 1 [file oncotarget-07-51462-s001.pdf]

Different metabolic responses to PI3K inhibition in NSCLC cells harboring wild-type and G12C mutant KRAS

SUPPLEMENTARY DATA

Cell cultures and drug treatment

Expression vector containing the coding region of KRAS-WT was subjected to direct mutagenesis to generate G12C mutant. The NCI-H1299 cell line was purchased by ATCC and grown in RPMI-1640 medium. Clones were obtained by transfecting the NCI-H1299 cell line with the expression plasmids encoding for the mutant KRAS-G12C and the KRAS-WT as a control. Transfected cells were seeded at low density with G418-containing medium, to select positive clones. Isolated clones were then tested for the presence of the overexpressing form of KRAS (either mutant or WT) by western blot analyses and by PCR and sequencing. All clones were grown in medium including 500 ug/ml of G418 (Gibco).

The MTS assays (Promega) were performed as described in [6]. Briefly, cells were seeded at 12,000 cells/ml in 96-well plates. After 24h, cells were treated with BEZ235 and BKM120 at different concentrations. After 72h from treatment start, cell viability was assessed by MTS assay. The assay was performed by adding 10 µL of MTS reagent directly to culture wells, including cell-free wells which were used as blank measurement, incubating for about 3h and then recording the absorbance (Abs) at 490nm with a 96-well plate reader (TECAN).

Absolute metabolite profiling

Targeted metabolomics analysis of samples was performed using the Biocrates AbsoluteIDQTM p180 kit (Biocrates Life Science AG, Innskruck, Austria). This validated targeted assay allows for simultaneous detection and quantification of metabolites in biological samples in a high-throughput manner. The metabolite extracts were processed following the instructions by the manufacturer and analyzed on a triple-quadropole mass spectrometer (AB SCIEX triple-quad 5500) operating in the multiple reaction monitoring (MRM-MS) mode. The assay is based on PITC (phenylisothiocyanate)-derivatization in the presence of internal standards for the analysis of aminoacids and biogenic amines resolved and quantified by liquid chromatography- tandem mass spectrometry (LC-MS/MS) using scheduled MRMs. Subsequent flow injection analysis tandem mass spectrometry (FIA-MS/MS) is performed to analyze acylcarnitines, glycerophospholipids, hexose. MRM detection is used for quantification applying spectra parsing algorithm integrated into the MetIQ software (Biocrates Life Science AG, Innskruck, Austria). Concentrations are calculated and evaluated by comparing measured analytes in a defined extracted ion count section to those of specific labeled internal standards or non-labeled ones, provided by the kit. The measurements are made in a 96-well format. Seven calibration standards, four quality control samples, three zero samples (methanol) and one blank (solvents) are integrated into the plate. The limit of detection for the individual metabolites is set to three times the values of the “zero samples”. For analytical specification refer to the AbsoluteIDQp180 Kit manuals. Five quality controls (QCs) were included in the analysis to assess the quality of the data. The CVs of all metabolites measured in the (QCs) were below 15%. The list of all the measurable metabolites is provided in supplementary table S5.

For glycerophospholipids, the precise position of the double bonds and the distribution of the carbon atoms in different fatty acid side chains cannot be determined with this technology. Lipid side-chain composition is abbreviated as Cx:y, where x denotes the number of carbons in the side chain and y the number of double bonds. The nature of fatty acids linkage is expressed as aa for diacyl or ae for acyl-alkyl. For example, PCaaC32:1 denotes diacyl-phosphatidylcholine with 32 carbons in the two fatty acids side chains and a single double bond in one of them.

Cyto-ID assay

Twenty-four hours after cell plating, BEZ235 (50 nM) or BKM120 (2 µM) was added. Forty-eight hours later, Cyto-ID stained autophagic compartments were measured using the Cyto-ID kit (Enzo Life Sciences), according to the manufacturer’s instructions.

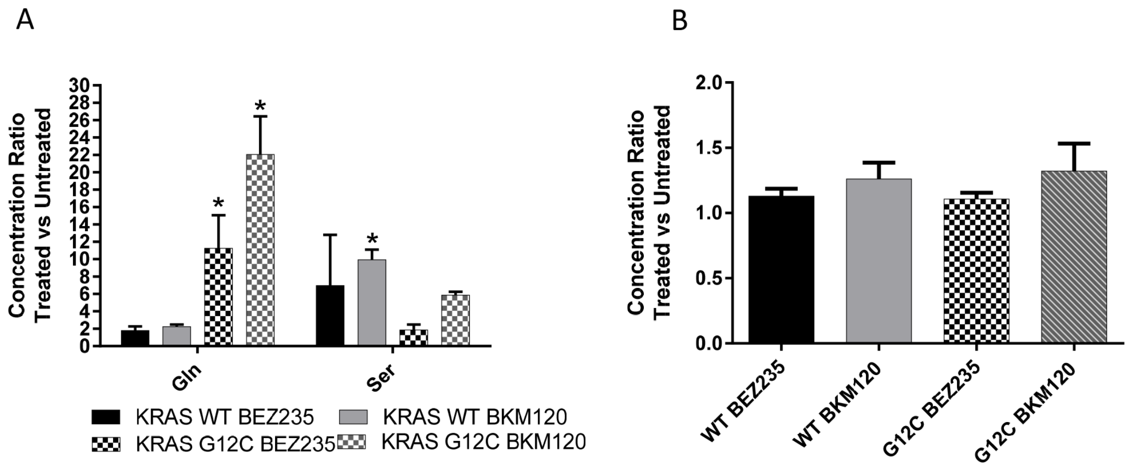

Supplementary Figure S1: Alterations of metabolite uptake in NSCLC cell clones harboring KRAS-G12C or KRAS-WT isoforms after PI3K inhibitor treatments. *Panel* **A.** Concentration ratio of glutamine (Gln) and serine (Ser) in KRAS-G12C and KRAS-WT clones after 48h of BEZ235 (25 nM) or BKM120 (1 µM) treatment in culture conditioned medium. *Panel* **B.** Hexose concentration ratio in KRAS-G12C and KRAS-WT clones 48h after BEZ235 (25 nM) or BKM120 (1 µM) treatment in culture conditioned medium. Data are the mean ± SD of three independent experiments. *P<0.05, ANOVA, Tukey-Kramer HSD.

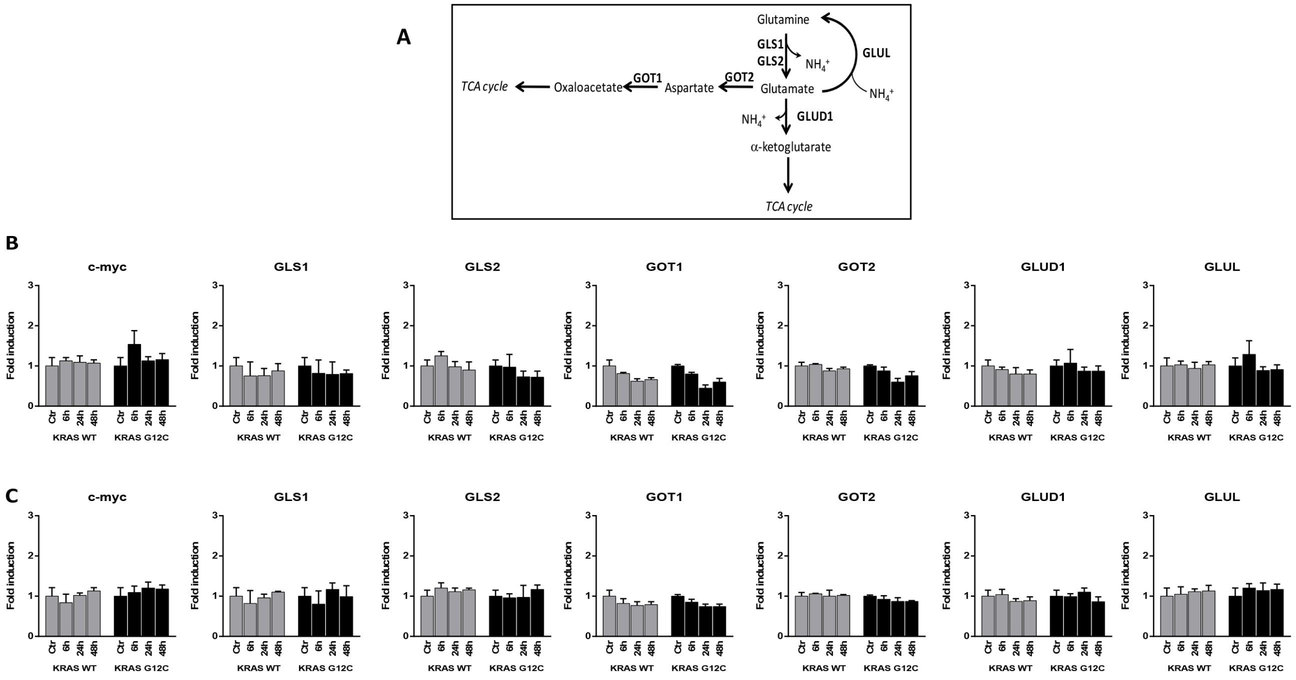

Supplementary Figure S2: Expression levels of enzymes involved in glutaminolysis or related pathways after PI3K inhibitor treatment. *Panel* **A.** Scheme showing the enzymes involved in glutaminolysis investigated in this study. GLS1 (Glutaminase kidney-type), GLS2 (Glutaminase liver-type), GOT1 (Aspartate aminotransferase, cytoplasmic), GOT2 (Aspartate aminotransferase, mitochondrial), GLUD1 (glutamate dehydrogenase 1), GLUL (glutamate-ammonia ligase). *Panels* **B-C.** Relative expression levels of genes encoding c-myc and glutaminolysis enzymes determined by real-time PCR, in NSCLC clones harboring KRAS-G12C or KRAS-WT isoforms, after 6, 24, 48h of 25 nM BEZ235 (*B*) or 1 µM BKM120 (*C*). Control samples of both clones were arbitrarily set to 1.

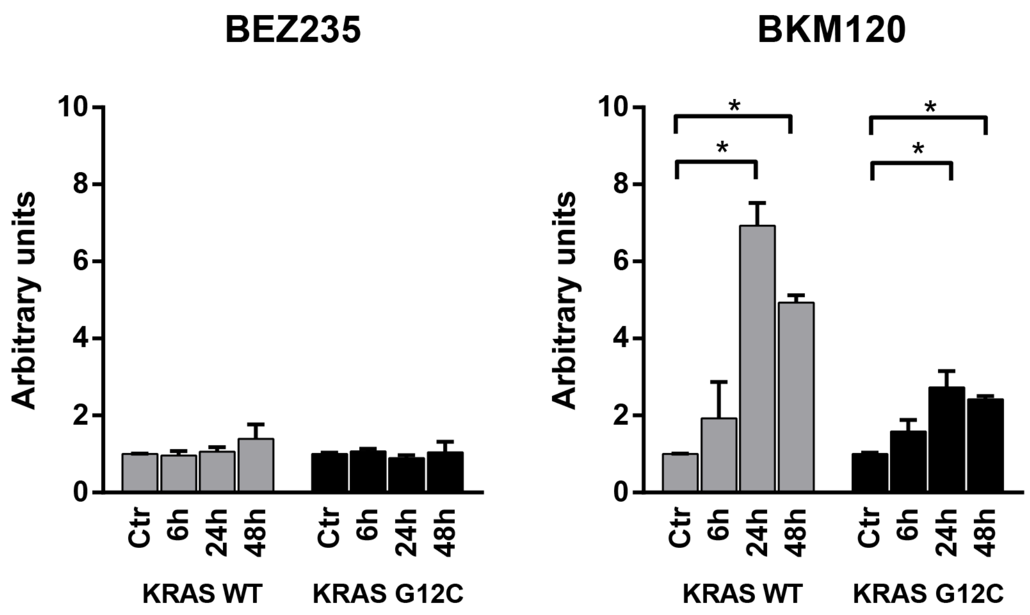

Supplementary Figure S3: Caspase 3 and 7 activities in KRAS-G12C and KRAS-WT clones after BEZ235 (50 nM) and BKM120 (2 µM) treatment assessed at the times indicated. The average of three different biological replicates and SD are shown. *P<0.05, ANOVA, Tukey-Kramer HSD.

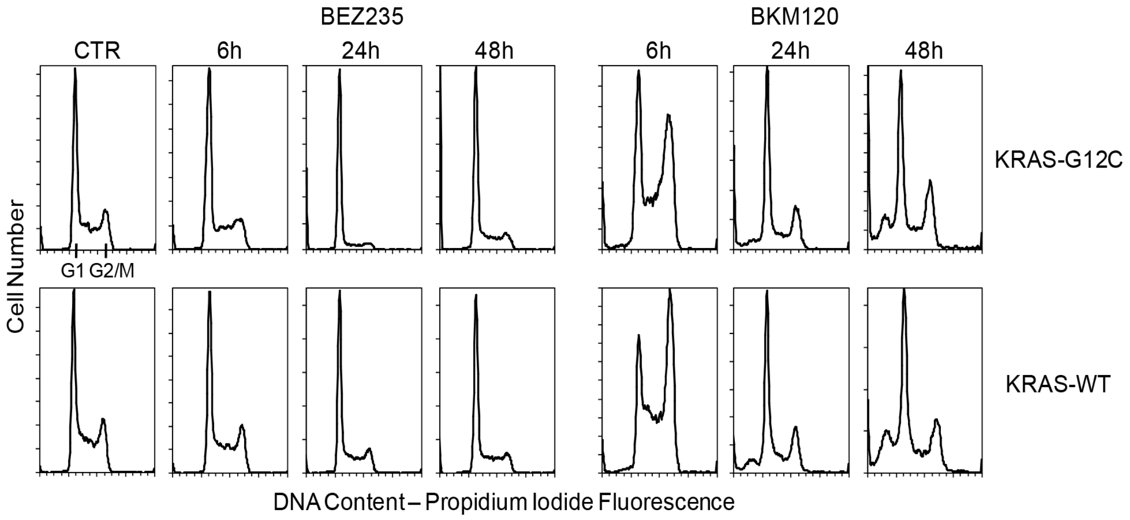

Supplementary Figure S4: Cell cycle phase distribution in KRAS-G12C and KRAS-WT cells treated with 50 nM BEZ235 and 2 µM BKM120.

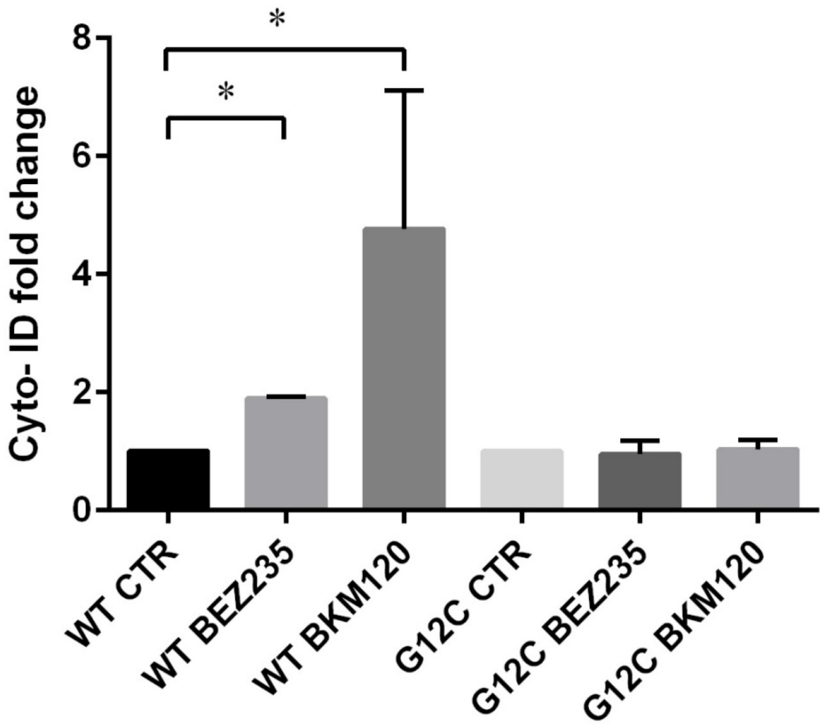

Supplementary Figure S5: Autophagy detection in KRAS-G12C and KRAS-WT cells treated with 50nM BEZ235 and 2 µM BKM120. Data are expressed as fold change of treated clones vs controls, which where arbitrarily set to 1. Histograms represent mean ± SD of three independent experiments *P<0.05, Unpaired t-test.

Supplementary Table S1: Micromolar concentrations of quantified metabolites in NSCLC cell clones harboring KRAS-G12C or KRAS-WT isoforms treated with PI3K inhibitors BEZ235 (25 nM) or BKM120 (1 µM) at 6, 24, 48h

See Supplementary File 1

Supplementary Table S2: Concentration ratio (treated versus untreated) of the discriminant metabolites (from OPLS-DA, s-plot) in KRAS-WT and KRAS-G12C clones treated with BEZ235 (25 nM) or BKM120 (1 µM), at 6, 24 and 48h

See Supplementary File 1

Supplementary Table S3: Cell cycle distribution after PI3K inhibitors at low doses (25 nM BEZ235, 1 µM BKM120)

		KRAS G12C			KRAS WT		
		**%G1**	**%S**	**%G2/M**	**%G1**	**%S**	**%G2/M**
**CTR**		41.9	47.2	10.9	32.7	52.8	14.5
	6h	51.6	39.3	9.1	36.5	49.8	13.7
**BEZ235**	24h	60.9	30.8	8.3	45.6	44.3	10.1
	48h	59.8	33.0	7.2	45.1	44.0	10.9
	6h	39.8	45.5	14.7	29.8	52.7	17.5
**BKM120**	24h	41.8	46.1	12.1	31.6	53.7	14.7
	48h	55.5	33.1	11.4	44.1	42.8	13.1

Percentages of cells in the cell cycle phase analysis

Supplementary Table S4: Cell cycle distribution after PI3K inhibitors at high doses (50 nM BEZ235, 2 µM BKM120)

		KRAS G12C			KRAS WT		
		**%G1**	**%S**	**%G2/M**	**%G1**	**%S**	**%G2/M**
**CTR**		41.9	47.2	10.9	32.7	52.8	14.5
	6h	50.7	39.2	10.1	35.3	49.9	14.8
**BEZ235**	24h	77.9	17.2	4.9	49.9	40.9	9.2
	48h	56.9	36.9	6.2	50.5	40.9	8.6
	6h	25.0	41.0	34.0	13.2	55.1	31.7
**BKM120**	24h	47.7	35.4	16.9	44.0	39.0	17.0
	48h	41.1	36.6	22.3	40.4	41.8	17.8

Percentages of cells in the cell cycle phase analysis

Supplementary Table S5: List of metabolites determined using the Biocrates Absolute IDQ p180 kit

See Supplementary File 2
