# Supplementary material for: Different metabolic responses to PI3K inhibition in NSCLC cells harboring wild-type and G12C mutant KRAS
Source: Oncotarget. 2016 Jun 6;7(32):51462–72. doi: 10.18632/oncotarget.9849 (PMC5239488; doi:10.18632/oncotarget.9849)
Supplement: Supplementary file 2 [file oncotarget-07-51462-s002.docx]

**Supplementary Table S1.** Micromolar concentrations of quantified metabolites in NSCLC cell clones harboring KRAS-G12C or KRAS-WT isoforms treated with PI3K inhibitors BEZ235 (25 nM) or BKM120 (1 µM) at 6, 24, 48h.

|  | **WT KRAS** | | | | | | | | | **G12C KRAS** | | | | | | | | |
| --- | --- | --- | --- | --- | --- | --- | --- | --- | --- | --- | --- | --- | --- | --- | --- | --- | --- | --- |
|  | **Untreated** | | | **BEZ235 6h** | | | **BKM120 6h** | | | **Untreated** | | | **BEZ235 6h** | | | **BKM120 6h** | | |
| **Metabolite** | **R1^a)^** | **R2^b)^** | **R3^c)^** | **R1^a)^** | **R2^b)^** | **R3^c)^** | **R1^a)^** | **R2^b)^** | **R3^c)^** | **R1^a)^** | **R2^b)^** | **R3^c)^** | **R1^a)^** | **R2^b)^** | **R3^c)^** | **R1^a)^** | **R2^b)^** | **R3^c)^** |
| lysoPC a C16:0 | 5.00 | 7.13 | 4.87 | 4.26 | 7.59 | 18.43 | 4.94 | 5.77 | 6.98 | 10.04 | 9.71 | 11.04 | 8.91 | 10.99 | 13.16 | 9.64 | 9.85 | 7.65 |
| lysoPC a C16:1 | 0.95 | 1.22 | 0.90 | 1.01 | 1.32 | 1.89 | 1.01 | 0.94 | 1.15 | 1.71 | 1.38 | 1.16 | 1.22 | 2.09 | 1.89 | 1.55 | 1.67 | 1.37 |
| lysoPC a C17:0 | 0.34 | 0.43 | 0.28 | 0.31 | 0.46 | 1.40 | 0.33 | 0.34 | 0.41 | 0.64 | 0.63 | 0.94 | 0.62 | 0.78 | 1.04 | 0.68 | 0.65 | 0.56 |
| lysoPC a C18:0 | 2.09 | 2.88 | 2.05 | 1.72 | 3.05 | 15.46 | 2.29 | 2.44 | 2.99 | 4.47 | 4.07 | 9.90 | 4.57 | 5.35 | 9.83 | 4.90 | 4.97 | 4.06 |
| lysoPC a C18:1 | 4.98 | 7.50 | 5.52 | 5.15 | 7.62 | 13.53 | 5.37 | 5.87 | 6.93 | 8.38 | 6.86 | 6.76 | 6.79 | 10.47 | 9.26 | 7.90 | 8.01 | 6.01 |
| lysoPC a C18:2 | 0.36 | 0.44 | 0.35 | 0.37 | 0.44 | 0.85 | 0.36 | 0.37 | 0.42 | 0.57 | 0.42 | 0.54 | 0.42 | 0.71 | 0.82 | 0.51 | 0.56 | 0.41 |
| lysoPC a C20:3 | 0.21 | 0.27 | 0.22 | 0.22 | 0.31 | 0.67 | 0.20 | 0.21 | 0.30 | 0.41 | 0.28 | 0.49 | 0.29 | 0.52 | 0.68 | 0.40 | 0.36 | 0.30 |
| lysoPC a C20:4 | 0.17 | 0.40 | 0.34 | 0.21 | 0.43 | 1.09 | 0.29 | 0.30 | 0.41 | 0.57 | 0.38 | 0.60 | 0.32 | 0.66 | 1.02 | 0.53 | 0.40 | 0.31 |
| lysoPC a C24:0 | 0.21 | 0.25 | 0.23 | 0.18 | 0.22 | 0.29 | 0.20 | 0.17 | 0.21 | 0.31 | 0.22 | 0.23 | 0.26 | 0.46 | 0.30 | 0.32 | 0.32 | 0.29 |
| lysoPC a C26:0 | 0.61 | 0.61 | 0.67 | 0.52 | 0.58 | 0.78 | 0.55 | 0.44 | 0.60 | 0.96 | 0.69 | 0.57 | 0.84 | 1.33 | 0.79 | 0.87 | 0.84 | 0.72 |
| lysoPC a C26:1 | 0.30 | 0.38 | 0.31 | 0.31 | 0.38 | 0.42 | 0.31 | 0.25 | 0.38 | 0.49 | 0.36 | 0.40 | 0.55 | 0.78 | 0.47 | 0.53 | 0.61 | 0.45 |
| lysoPC a C28:0 | 0.72 | 1.01 | 0.75 | 0.72 | 1.21 | 1.33 | 0.74 | 0.67 | 1.00 | 1.35 | 1.03 | 0.91 | 1.29 | 1.70 | 0.93 | 1.18 | 1.16 | 1.05 |
| lysoPC a C28:1 | 0.58 | 0.74 | 0.62 | 0.60 | 0.73 | 0.95 | 0.57 | 0.54 | 0.68 | 0.91 | 0.75 | 0.61 | 0.96 | 1.22 | 0.75 | 0.95 | 0.92 | 0.74 |
| PC aa C24:0 | 0.25 | 0.26 | 0.29 | 0.19 | 0.17 | 0.27 | 0.23 | 0.17 | 0.20 | 0.25 | 0.16 | 0.14 | 0.14 | 0.26 | 0.24 | 0.21 | 0.19 | 0.16 |
| PC aa C28:1 | 2.00 | 2.61 | 2.26 | 1.89 | 2.62 | 3.45 | 1.98 | 1.86 | 2.23 | 2.38 | 1.86 | 1.38 | 1.95 | 2.73 | 1.55 | 2.02 | 1.88 | 1.64 |
| PC aa C30:0 | 11.17 | 15.50 | 8.42 | 10.83 | 22.22 | 22.45 | 11.16 | 10.42 | 14.93 | 13.07 | 11.35 | 9.23 | 15.18 | 17.91 | 6.63 | 11.93 | 11.04 | 11.35 |
| PC aa C30:2 | 0.46 | 0.57 | 0.32 | 0.48 | 0.77 | 0.69 | 0.32 | 0.37 | 0.55 | 0.77 | 0.70 | 0.50 | 0.75 | 0.91 | 0.44 | 0.62 | 0.68 | 0.60 |
| PC aa C32:0 | 134.84 | 181.53 | 105.09 | 108.69 | 226.46 | 274.56 | 115.68 | 106.36 | 114.73 | 124.10 | 104.89 | 77.96 | 125.49 | 156.37 | 46.54 | 88.51 | 73.04 | 88.99 |
| PC aa C32:1 | 1212.67 | 1729.86 | 1037.92 | 1036.23 | 2015.88 | 2234.49 | 1135.16 | 1013.97 | 1251.07 | 1178.40 | 1058.83 | 796.41 | 1139.37 | 1415.99 | 500.17 | 779.66 | 765.19 | 739.50 |
| PC aa C32:2 | 157.59 | 226.03 | 139.44 | 135.45 | 230.71 | 271.37 | 134.47 | 133.56 | 156.06 | 168.34 | 155.29 | 103.00 | 164.90 | 187.65 | 75.84 | 117.88 | 115.24 | 102.08 |
| PC aa C32:3 | 8.26 | 11.99 | 7.11 | 7.11 | 12.58 | 13.83 | 6.98 | 6.69 | 8.01 | 9.44 | 7.92 | 5.87 | 8.97 | 10.78 | 4.12 | 6.37 | 6.27 | 5.61 |
| PC aa C34:1 | 2590.89 | 3280.84 | 1991.96 | 2065.84 | 3913.70 | 3217.31 | 2344.84 | 1945.24 | 2552.69 | 1732.02 | 1472.37 | 1410.90 | 1539.68 | 2007.15 | 841.94 | 1173.21 | 1236.21 | 1192.09 |
| PC aa C34:2 | 895.25 | 1357.15 | 760.92 | 801.16 | 1589.33 | 1829.90 | 814.90 | 794.33 | 1029.95 | 866.89 | 792.54 | 595.05 | 910.98 | 1095.49 | 387.59 | 645.02 | 607.79 | 570.06 |
| PC aa C34:3 | 56.51 | 82.74 | 48.50 | 50.42 | 96.37 | 110.75 | 50.98 | 48.26 | 62.12 | 67.35 | 59.43 | 45.31 | 70.43 | 86.69 | 30.65 | 46.67 | 46.49 | 40.98 |
| PC aa C34:4 | 10.00 | 13.90 | 8.69 | 9.28 | 16.35 | 18.89 | 9.29 | 8.33 | 10.75 | 12.16 | 11.17 | 7.47 | 12.12 | 14.68 | 6.24 | 8.26 | 8.75 | 7.47 |
| PC aa C36:0 | 12.73 | 21.06 | 10.86 | 13.92 | 28.72 | 34.07 | 12.28 | 11.55 | 18.29 | 13.23 | 12.44 | 9.54 | 14.97 | 18.55 | 5.57 | 9.26 | 9.79 | 8.73 |
| PC aa C36:1 | 167.44 | 257.67 | 140.54 | 158.87 | 407.88 | 452.48 | 168.40 | 167.95 | 208.73 | 148.39 | 111.26 | 112.14 | 179.39 | 211.08 | 56.16 | 103.13 | 100.74 | 109.10 |
| PC aa C36:2 | 1293.36 | 1961.16 | 1104.45 | 1188.95 | 2529.86 | 2643.32 | 1193.95 | 1133.07 | 1598.85 | 992.03 | 860.27 | 722.50 | 1069.24 | 1362.78 | 426.75 | 714.03 | 692.80 | 667.33 |
| PC aa C36:3 | 189.48 | 297.10 | 158.58 | 176.11 | 410.66 | 418.72 | 173.52 | 167.29 | 234.92 | 202.95 | 167.66 | 139.76 | 220.12 | 275.36 | 87.71 | 135.61 | 137.71 | 125.95 |
| PC aa C36:4 | 78.45 | 119.80 | 66.80 | 74.38 | 155.78 | 172.53 | 74.01 | 68.86 | 91.96 | 100.40 | 89.32 | 68.71 | 108.84 | 128.07 | 46.81 | 68.87 | 70.77 | 63.94 |
| PC aa C36:5 | 31.85 | 48.07 | 28.40 | 30.79 | 57.32 | 67.56 | 30.60 | 27.86 | 34.88 | 46.44 | 40.57 | 30.84 | 48.52 | 57.59 | 21.89 | 32.25 | 32.36 | 29.09 |
| PC aa C36:6 | 16.59 | 25.19 | 13.29 | 13.74 | 31.75 | 34.72 | 13.06 | 12.92 | 17.44 | 15.47 | 12.42 | 10.28 | 15.86 | 20.11 | 6.46 | 10.51 | 9.66 | 9.06 |
| PC aa C38:0 | 8.68 | 14.57 | 7.70 | 9.22 | 21.21 | 23.22 | 9.25 | 8.56 | 12.03 | 10.99 | 8.78 | 8.16 | 12.89 | 14.90 | 4.75 | 7.94 | 8.12 | 7.32 |
| PC aa C38:1 | 0.04 | 0.04 | 0.04 | 0.04 | 0.04 | 0.22 | 0.04 | 0.04 | 0.04 | 0.84 | 0.84 | 0.04 | 0.78 | 1.07 | 0.24 | 0.89 | 0.40 | 0.90 |
| PC aa C38:3 | 25.57 | 40.02 | 20.63 | 25.25 | 60.18 | 62.08 | 26.11 | 23.25 | 33.20 | 28.18 | 21.83 | 21.36 | 32.03 | 40.63 | 11.56 | 20.12 | 20.17 | 19.32 |
| PC aa C38:4 | 42.03 | 67.97 | 33.98 | 39.40 | 91.07 | 101.58 | 42.10 | 37.60 | 53.44 | 53.37 | 43.73 | 38.13 | 63.00 | 74.53 | 23.84 | 39.67 | 41.15 | 38.47 |
| PC aa C38:5 | 59.10 | 90.63 | 52.75 | 55.64 | 118.37 | 138.11 | 59.11 | 54.58 | 72.51 | 71.35 | 58.60 | 51.21 | 77.53 | 94.76 | 33.78 | 51.13 | 52.60 | 47.58 |
| PC aa C38:6 | 53.70 | 78.61 | 43.87 | 47.32 | 98.28 | 110.30 | 50.12 | 45.55 | 59.76 | 61.22 | 53.75 | 42.37 | 67.87 | 81.39 | 28.95 | 45.56 | 46.83 | 40.36 |
| PC aa C40:2 | 2.66 | 4.34 | 2.36 | 2.80 | 7.29 | 7.46 | 3.23 | 2.73 | 3.77 | 2.35 | 2.31 | 2.09 | 2.91 | 3.70 | 1.29 | 2.20 | 2.04 | 2.12 |
| PC aa C40:3 | 2.35 | 3.63 | 1.97 | 2.40 | 5.22 | 5.24 | 2.36 | 2.00 | 3.02 | 2.29 | 1.75 | 2.11 | 2.47 | 3.17 | 1.08 | 1.55 | 1.57 | 1.44 |
| PC aa C40:4 | 3.47 | 6.34 | 3.51 | 4.16 | 10.00 | 9.30 | 4.26 | 4.06 | 5.37 | 4.23 | 3.40 | 3.36 | 5.30 | 6.33 | 1.92 | 3.36 | 3.28 | 3.48 |
| PC aa C40:5 | 14.42 | 22.47 | 11.32 | 14.99 | 33.19 | 36.88 | 14.53 | 13.50 | 18.99 | 14.81 | 12.09 | 11.49 | 17.12 | 21.43 | 6.52 | 11.11 | 11.46 | 10.44 |
| PC aa C40:6 | 20.50 | 33.22 | 19.83 | 20.59 | 47.14 | 52.94 | 21.35 | 19.81 | 28.08 | 26.81 | 22.54 | 19.08 | 30.87 | 38.10 | 13.14 | 21.00 | 21.10 | 19.94 |
| PC aa C42:0 | 0.49 | 0.86 | 0.54 | 0.43 | 1.18 | 1.00 | 0.42 | 0.42 | 0.62 | 0.65 | 0.51 | 0.42 | 0.63 | 0.78 | 0.36 | 0.51 | 0.50 | 0.61 |
| PC aa C42:1 | 0.86 | 0.85 | 0.52 | 0.62 | 1.06 | 1.40 | 0.54 | 0.51 | 0.56 | 0.83 | 0.70 | 0.49 | 0.94 | 1.00 | 0.43 | 0.66 | 0.65 | 0.90 |
| PC aa C42:2 | 0.63 | 1.26 | 0.75 | 1.00 | 1.96 | 2.09 | 0.96 | 0.69 | 1.08 | 1.16 | 0.82 | 0.89 | 1.35 | 1.76 | 0.64 | 0.89 | 0.85 | 1.12 |
| PC aa C42:4 | 0.70 | 1.10 | 0.72 | 0.82 | 1.71 | 1.51 | 0.62 | 0.63 | 1.12 | 0.81 | 0.63 | 0.66 | 0.81 | 1.24 | 0.44 | 0.61 | 0.73 | 0.69 |
| PC aa C42:5 | 2.38 | 3.18 | 1.77 | 2.39 | 4.90 | 6.09 | 2.38 | 1.78 | 2.98 | 2.31 | 1.73 | 1.72 | 2.71 | 3.20 | 0.78 | 1.63 | 1.69 | 1.66 |
| PC aa C42:6 | 5.39 | 9.79 | 5.22 | 5.95 | 14.65 | 14.98 | 5.22 | 5.71 | 8.37 | 5.80 | 4.65 | 4.45 | 7.03 | 8.59 | 2.78 | 4.51 | 4.76 | 3.97 |
| PC ae C30:0 | 2.41 | 3.30 | 1.87 | 2.32 | 4.96 | 5.26 | 2.53 | 2.38 | 3.36 | 3.65 | 3.00 | 2.55 | 4.09 | 4.92 | 1.90 | 3.18 | 3.05 | 2.97 |
| PC ae C30:1 | 1.22 | 1.90 | 1.02 | 1.22 | 2.46 | 2.77 | 1.31 | 1.25 | 1.74 | 1.67 | 1.49 | 1.22 | 1.98 | 2.23 | 0.94 | 1.50 | 1.44 | 1.40 |
| PC ae C30:2 | 0.18 | 0.25 | 0.15 | 0.18 | 0.32 | 0.34 | 0.16 | 0.17 | 0.24 | 0.25 | 0.25 | 0.17 | 0.28 | 0.31 | 0.15 | 0.23 | 0.22 | 0.19 |
| PC ae C32:1 | 204.96 | 289.80 | 174.19 | 170.70 | 346.52 | 405.33 | 182.79 | 167.03 | 209.71 | 226.65 | 197.21 | 147.37 | 220.49 | 267.62 | 91.07 | 150.53 | 142.84 | 140.40 |
| PC ae C32:2 | 47.37 | 68.87 | 40.13 | 38.53 | 77.88 | 89.31 | 41.87 | 37.76 | 49.20 | 42.34 | 37.44 | 30.22 | 42.43 | 55.42 | 18.63 | 29.33 | 27.08 | 26.35 |
| PC ae C34:0 | 31.01 | 37.97 | 22.23 | 28.43 | 51.81 | 60.68 | 27.10 | 22.09 | 22.62 | 33.40 | 26.37 | 19.94 | 32.56 | 38.24 | 13.83 | 23.61 | 22.06 | 28.05 |
| PC ae C34:1 | 453.40 | 662.23 | 374.08 | 373.84 | 868.83 | 989.48 | 417.73 | 391.43 | 525.73 | 419.26 | 365.18 | 299.45 | 450.28 | 553.31 | 167.04 | 283.39 | 269.36 | 271.43 |
| PC ae C34:2 | 182.06 | 269.49 | 150.77 | 156.92 | 349.58 | 394.88 | 168.53 | 156.41 | 203.36 | 165.66 | 139.14 | 112.66 | 175.92 | 215.19 | 66.35 | 107.59 | 104.17 | 103.26 |
| PC ae C34:3 | 24.25 | 36.52 | 20.07 | 20.72 | 45.95 | 48.07 | 21.29 | 20.31 | 28.05 | 21.12 | 18.68 | 15.27 | 21.77 | 27.89 | 8.81 | 14.05 | 13.69 | 12.25 |
| PC ae C36:0 | 11.63 | 17.89 | 9.66 | 11.59 | 20.82 | 23.38 | 9.75 | 9.65 | 13.03 | 10.22 | 8.65 | 7.26 | 10.71 | 14.31 | 4.42 | 7.50 | 7.72 | 6.61 |
| PC ae C36:1 | 94.13 | 138.17 | 79.35 | 85.55 | 205.79 | 226.21 | 86.76 | 83.22 | 111.30 | 90.60 | 74.38 | 63.57 | 100.84 | 120.99 | 35.59 | 58.82 | 58.30 | 61.32 |
| PC ae C36:2 | 178.93 | 262.83 | 145.60 | 157.81 | 369.25 | 395.79 | 164.04 | 152.00 | 203.60 | 160.17 | 136.69 | 118.04 | 177.41 | 223.40 | 70.58 | 112.18 | 112.18 | 103.86 |
| PC ae C36:3 | 71.64 | 116.22 | 59.72 | 67.43 | 155.94 | 157.63 | 65.45 | 61.52 | 89.79 | 68.79 | 56.00 | 49.95 | 73.56 | 94.11 | 27.53 | 44.07 | 44.24 | 41.44 |
| PC ae C36:4 | 45.15 | 66.16 | 38.12 | 41.60 | 93.90 | 99.56 | 44.41 | 41.13 | 56.05 | 55.08 | 47.80 | 37.38 | 60.20 | 74.07 | 23.78 | 37.49 | 38.71 | 35.93 |
| PC ae C36:5 | 27.17 | 40.68 | 23.36 | 24.73 | 53.05 | 57.94 | 25.42 | 24.70 | 32.59 | 35.73 | 30.86 | 23.67 | 38.39 | 47.39 | 15.84 | 25.07 | 24.32 | 22.54 |
| PC ae C38:0 | 9.10 | 14.50 | 8.01 | 8.71 | 17.63 | 19.89 | 8.29 | 8.81 | 10.76 | 11.06 | 9.44 | 6.99 | 11.41 | 14.13 | 5.24 | 8.12 | 8.03 | 7.00 |
| PC ae C38:1 | 5.82 | 8.94 | 5.38 | 6.46 | 14.26 | 15.80 | 6.09 | 5.34 | 7.39 | 5.57 | 4.96 | 5.02 | 6.49 | 8.80 | 2.69 | 3.78 | 4.26 | 4.48 |
| PC ae C38:2 | 28.81 | 44.28 | 25.58 | 27.92 | 60.99 | 65.49 | 26.52 | 25.27 | 35.45 | 26.77 | 22.86 | 21.65 | 28.85 | 36.01 | 12.15 | 18.76 | 18.26 | 18.49 |
| PC ae C38:3 | 20.85 | 35.61 | 17.11 | 20.27 | 55.46 | 53.99 | 19.37 | 18.76 | 29.76 | 20.02 | 15.65 | 14.99 | 21.62 | 27.32 | 7.94 | 13.31 | 12.80 | 12.71 |
| PC ae C38:4 | 22.22 | 37.23 | 20.29 | 22.47 | 54.75 | 58.49 | 22.27 | 19.97 | 29.72 | 27.24 | 21.40 | 20.05 | 31.23 | 39.78 | 11.57 | 18.69 | 18.45 | 17.53 |
| PC ae C38:5 | 34.63 | 55.83 | 29.60 | 34.69 | 76.97 | 82.05 | 35.06 | 32.17 | 46.99 | 44.70 | 36.49 | 30.53 | 49.86 | 59.50 | 19.32 | 31.68 | 31.13 | 28.99 |
| PC ae C38:6 | 32.81 | 52.35 | 26.94 | 31.80 | 71.11 | 78.64 | 31.20 | 29.33 | 40.01 | 41.51 | 33.93 | 27.86 | 44.13 | 55.81 | 17.25 | 29.52 | 28.58 | 27.18 |
| PC ae C40:1 | 2.04 | 2.96 | 1.74 | 2.06 | 4.08 | 5.99 | 1.92 | 1.76 | 2.38 | 2.49 | 2.23 | 1.59 | 2.65 | 3.21 | 1.65 | 1.92 | 1.95 | 1.84 |
| PC ae C40:2 | 5.89 | 7.77 | 5.30 | 5.24 | 9.53 | 9.63 | 4.40 | 4.91 | 5.77 | 4.32 | 3.59 | 3.86 | 4.33 | 5.33 | 2.47 | 2.98 | 3.03 | 3.07 |
| PC ae C40:3 | 3.00 | 4.31 | 2.96 | 2.85 | 5.82 | 6.01 | 2.82 | 2.52 | 3.27 | 2.98 | 2.70 | 2.66 | 3.17 | 4.34 | 1.69 | 2.31 | 2.26 | 2.13 |
| PC ae C40:4 | 4.04 | 5.93 | 3.19 | 4.18 | 8.55 | 9.92 | 3.79 | 3.73 | 5.34 | 4.45 | 3.80 | 3.49 | 5.28 | 6.49 | 2.18 | 3.42 | 3.29 | 3.45 |
| PC ae C40:5 | 12.53 | 19.80 | 10.53 | 12.43 | 30.17 | 30.25 | 11.39 | 10.91 | 15.65 | 15.17 | 12.49 | 10.95 | 16.41 | 21.70 | 6.31 | 10.87 | 10.72 | 10.14 |
| PC ae C40:6 | 10.70 | 16.71 | 9.07 | 10.79 | 24.03 | 25.99 | 10.87 | 9.73 | 14.23 | 13.74 | 10.97 | 10.15 | 15.19 | 19.11 | 6.05 | 10.03 | 9.69 | 8.85 |
| PC ae C42:0 | 2.50 | 3.42 | 2.04 | 2.38 | 5.24 | 4.95 | 2.30 | 1.82 | 2.89 | 2.56 | 2.42 | 1.98 | 2.80 | 3.34 | 1.16 | 2.11 | 2.11 | 1.82 |
| PC ae C42:2 | 1.25 | 1.41 | 0.86 | 1.10 | 2.10 | 3.06 | 0.92 | 0.87 | 1.24 | 1.27 | 1.05 | 1.01 | 1.28 | 1.58 | 0.95 | 1.04 | 1.05 | 0.94 |
| PC ae C42:3 | 1.32 | 1.78 | 1.09 | 1.17 | 2.61 | 3.70 | 1.16 | 1.13 | 1.61 | 1.53 | 1.18 | 1.34 | 1.38 | 1.84 | 1.13 | 1.15 | 1.10 | 1.14 |
| PC ae C42:4 | 1.11 | 1.49 | 0.64 | 0.93 | 2.19 | 2.31 | 0.92 | 0.83 | 1.35 | 1.11 | 1.00 | 1.09 | 1.21 | 1.55 | 0.69 | 0.84 | 0.78 | 0.82 |
| PC ae C42:5 | 2.46 | 3.78 | 2.29 | 2.38 | 6.06 | 6.15 | 2.36 | 2.34 | 3.24 | 2.69 | 2.18 | 2.08 | 2.96 | 3.62 | 1.33 | 2.08 | 1.92 | 1.98 |
| PC ae C44:3 | 0.58 | 0.72 | 0.56 | 0.49 | 0.81 | 0.98 | 0.41 | 0.46 | 0.62 | 0.63 | 0.50 | 0.45 | 0.66 | 0.70 | 0.37 | 0.48 | 0.50 | 0.39 |
| PC ae C44:4 | 0.49 | 0.68 | 0.49 | 0.39 | 0.98 | 1.06 | 0.38 | 0.41 | 0.60 | 0.52 | 0.40 | 0.37 | 0.50 | 0.76 | 0.32 | 0.43 | 0.34 | 0.41 |
| PC ae C44:5 | 0.63 | 0.82 | 0.48 | 0.59 | 1.23 | 1.38 | 0.60 | 0.60 | 0.64 | 0.59 | 0.51 | 0.51 | 0.57 | 0.85 | 0.50 | 0.37 | 0.42 | 0.39 |
| PC ae C44:6 | 0.58 | 0.87 | 0.53 | 0.66 | 1.34 | 1.62 | 0.46 | 0.48 | 0.83 | 0.71 | 0.59 | 0.50 | 0.70 | 0.86 | 0.31 | 0.54 | 0.48 | 0.55 |
| Sugars | 3453.91 | 2795.92 | 1790.71 | 2553.19 | 4043.11 | 3789.36 | 2941.91 | 1856.80 | 2911.68 | 2052.24 | 2023.16 | 2137.81 | 2656.00 | 2534.44 | 4597.72 | 2545.73 | 2793.38 | 2894.61 |
| Ala | 271.00 | 270.67 | 189.33 | 433.33 | 483.33 | 87.67 | 267.33 | 232.67 | 310.00 | 793.33 | 693.33 | 109.67 | 1420.00 | 1643.33 | 376.67 | 1243.33 | 1236.67 | 1206.67 |
| Arg | 114.00 | 98.00 | 70.00 | 109.67 | 160.67 | 133.67 | 111.33 | 81.00 | 123.00 | 111.67 | 113.00 | 92.33 | 152.00 | 172.33 | 155.67 | 150.00 | 161.00 | 158.67 |
| Asn | 653.33 | 660.00 | 433.33 | 943.33 | 1033.33 | 440.00 | 593.33 | 516.67 | 776.67 | 703.33 | 550.00 | 326.33 | 1076.67 | 1196.67 | 763.33 | 990.00 | 923.33 | 876.67 |
| Asp | 143.33 | 156.00 | 103.33 | 235.33 | 232.67 | 180.00 | 154.67 | 133.67 | 191.67 | 207.00 | 186.67 | 120.33 | 286.00 | 353.33 | 212.33 | 315.00 | 304.33 | 353.33 |
| Cit | 4.63 | 4.23 | 3.33 | 6.73 | 5.47 | 4.23 | 4.53 | 3.80 | 3.97 | 3.90 | 3.17 | 3.00 | 6.83 | 8.17 | 4.80 | 7.03 | 5.33 | 5.33 |
| Gln | 1056.67 | 1106.67 | 646.67 | 1573.33 | 1836.67 | 1366.67 | 1130.00 | 980.00 | 1266.67 | 526.67 | 456.67 | 620.00 | 1103.33 | 1193.33 | 1463.33 | 853.33 | 1056.67 | 966.67 |
| Glu | 3866.67 | 3866.67 | 2806.67 | 4866.67 | 5400.00 | 2180.00 | 3093.33 | 2776.67 | 4066.67 | 2866.67 | 2406.67 | 1220.00 | 3466.67 | 4166.67 | 2923.33 | 3466.67 | 3500.00 | 3600.00 |
| Gly | 546.67 | 540.00 | 327.00 | 773.33 | 846.67 | 208.33 | 530.00 | 476.67 | 646.67 | 366.67 | 296.67 | 120.00 | 633.33 | 920.00 | 473.33 | 620.00 | 670.00 | 586.67 |
| His | 35.00 | 33.67 | 20.63 | 42.33 | 50.00 | 31.63 | 34.67 | 29.27 | 41.33 | 33.23 | 29.73 | 16.70 | 50.33 | 58.33 | 40.33 | 50.33 | 55.00 | 50.67 |
| Ile | 117.33 | 107.00 | 69.33 | 135.00 | 164.00 | 107.67 | 111.67 | 92.00 | 121.00 | 129.00 | 108.67 | 62.33 | 186.67 | 208.33 | 147.33 | 179.67 | 197.67 | 194.67 |
| Leu | 109.33 | 99.67 | 59.67 | 154.67 | 158.67 | 129.00 | 104.00 | 88.00 | 120.00 | 119.33 | 102.00 | 82.67 | 172.00 | 196.67 | 176.33 | 173.00 | 200.00 | 182.33 |
| Lys | 10.03 | 7.20 | 5.67 | 10.93 | 17.90 | 31.07 | 13.60 | 7.20 | 14.47 | 9.37 | 8.10 | 18.33 | 16.17 | 18.50 | 39.67 | 17.93 | 19.33 | 22.30 |
| Met | 23.87 | 26.83 | 16.73 | 28.63 | 38.33 | 33.27 | 25.77 | 21.60 | 28.93 | 23.57 | 22.37 | 16.47 | 42.67 | 46.33 | 39.33 | 37.67 | 40.33 | 39.67 |
| Orn | 5.23 | 4.27 | 3.05 | 4.87 | 8.43 | 4.17 | 5.17 | 3.15 | 6.07 | 5.63 | 4.80 | 3.28 | 7.37 | 8.87 | 7.13 | 7.87 | 8.43 | 8.40 |
| Phe | 27.43 | 25.10 | 16.53 | 32.87 | 40.00 | 33.33 | 28.63 | 24.10 | 30.83 | 25.97 | 22.93 | 17.33 | 42.33 | 47.67 | 44.33 | 41.33 | 46.33 | 44.67 |
| Pro | 275.00 | 278.67 | 173.33 | 319.67 | 346.67 | 131.00 | 237.67 | 222.67 | 285.67 | 270.33 | 244.67 | 103.67 | 360.00 | 386.67 | 296.33 | 360.00 | 383.33 | 366.67 |
| Ser | 64.67 | 60.67 | 36.00 | 104.67 | 117.33 | 174.00 | 79.67 | 59.67 | 86.67 | 83.00 | 71.33 | 82.67 | 154.33 | 176.67 | 273.33 | 136.67 | 145.00 | 138.67 |
| Thr | 90.67 | 90.67 | 58.67 | 150.67 | 167.33 | 80.67 | 93.67 | 77.00 | 105.00 | 102.33 | 80.33 | 50.33 | 210.67 | 208.67 | 141.67 | 176.67 | 157.00 | 163.33 |
| Trp | 8.87 | 8.23 | 5.67 | 10.57 | 12.50 | 10.43 | 9.47 | 7.87 | 10.20 | 8.83 | 7.53 | 5.47 | 13.47 | 14.53 | 12.87 | 13.77 | 14.57 | 14.23 |
| Tyr | 40.33 | 40.33 | 24.77 | 49.33 | 58.33 | 38.00 | 42.67 | 35.67 | 46.00 | 41.00 | 35.33 | 22.50 | 62.67 | 70.67 | 52.33 | 64.33 | 67.00 | 63.33 |
| Val | 31.40 | 31.00 | 19.23 | 39.67 | 49.67 | 47.67 | 37.00 | 31.40 | 40.00 | 33.67 | 28.80 | 25.17 | 55.00 | 63.33 | 62.33 | 55.67 | 58.67 | 55.67 |
| Ac-Orn | 1.16 | 1.06 | 0.82 | 1.96 | 2.16 | 0.73 | 1.03 | 0.83 | 1.71 | 1.40 | 1.08 | 0.39 | 2.03 | 2.71 | 1.50 | 2.10 | 2.32 | 2.06 |
| ADMA | 0.17 | 0.07 | 0.11 | 0.21 | 0.35 | 0.10 | 0.33 | 0.15 | 0.13 | 0.11 | 0.19 | 0.12 | 0.34 | 0.23 | 0.18 | 0.21 | 0.26 | 0.32 |
| alpha-AAA | 2.26 | 2.58 | 1.29 | 2.79 | 3.25 | 1.69 | 1.99 | 1.58 | 2.14 | 2.57 | 1.69 | 0.92 | 2.84 | 3.32 | 2.13 | 3.23 | 2.89 | 2.56 |
| Carnosine | 0.56 | 0.53 | 0.32 | 0.47 | 0.71 | 0.63 | 0.52 | 0.33 | 0.53 | 0.25 | 0.33 | 0.36 | 0.36 | 0.33 | 0.56 | 0.41 | 0.37 | 0.43 |
| DOPA | 0.07 | 0.06 | 0.03 | 0.07 | 0.07 | 0.03 | 0.07 | 0.06 | 0.05 | 0.14 | 0.10 | 0.05 | 0.23 | 0.22 | 0.10 | 0.15 | 0.14 | 0.19 |
| Kynurenine | 0.37 | 0.43 | 0.31 | 0.49 | 0.50 | 0.26 | 0.43 | 0.37 | 0.46 | 0.41 | 0.40 | 0.23 | 0.55 | 0.63 | 0.41 | 0.53 | 0.51 | 0.60 |
| Met-SO | 1.74 | 1.37 | 0.99 | 1.67 | 2.01 | 1.68 | 1.52 | 1.22 | 1.49 | 1.33 | 1.17 | 0.69 | 1.92 | 2.39 | 1.56 | 2.27 | 2.23 | 2.34 |
| Putrescine | 27.67 | 30.10 | 19.97 | 29.97 | 37.33 | 17.00 | 19.03 | 10.77 | 23.17 | 25.57 | 19.67 | 24.20 | 27.33 | 29.27 | 29.40 | 20.73 | 21.30 | 23.03 |
| Serotonin | 0.13 | 0.15 | 0.09 | 0.11 | 0.20 | 0.25 | 0.14 | 0.08 | 0.15 | 0.08 | 0.05 | 0.12 | 0.10 | 0.07 | 0.14 | 0.08 | 0.09 | 0.07 |
| Spermidine | 6.57 | 6.57 | 5.13 | 10.47 | 7.83 | 4.07 | 6.80 | 3.60 | 4.93 | 6.43 | 4.27 | 3.47 | 6.43 | 6.77 | 6.63 | 8.47 | 8.23 | 8.87 |
| Spermine | 3333.33 | 4.10 | 3.57 | 7.57 | 4.50 | 2.27 | 4.37 | 1.94 | 2.17 | 3.26 | 2.39 | 1.35 | 3.11 | 3.09 | 3.23 | 4.67 | 4.47 | 5.23 |
| t4-OH-Pro | 229.67 | 223.00 | 138.33 | 290.00 | 343.33 | 142.67 | 179.00 | 168.67 | 211.67 | 243.67 | 227.00 | 101.33 | 363.33 | 366.67 | 280.00 | 305.00 | 350.00 | 322.00 |
| Taurine | 204.67 | 202.00 | 158.67 | 202.33 | 225.33 | 73.33 | 188.67 | 176.67 | 198.67 | 216.33 | 204.67 | 119.33 | 238.67 | 243.67 | 208.33 | 235.00 | 236.67 | 229.00 |
| total DMA | 0.17 | 0.14 | 0.09 | 0.21 | 0.33 | 0.14 | 0.19 | 0.12 | 0.19 | 0.15 | 0.12 | 0.09 | 0.29 | 0.29 | 0.19 | 0.27 | 0.26 | 0.24 |
| SM (OH) C14:1 | 1.49 | 2.06 | 1.20 | 1.42 | 2.91 | 2.95 | 1.76 | 1.58 | 2.47 | 2.16 | 1.89 | 1.69 | 2.68 | 3.15 | 1.27 | 2.10 | 2.08 | 1.82 |
| SM (OH) C16:1 | 0.23 | 0.37 | 0.19 | 0.20 | 0.64 | 0.53 | 0.29 | 0.32 | 0.44 | 0.39 | 0.36 | 0.28 | 0.49 | 0.45 | 0.23 | 0.37 | 0.40 | 0.37 |
| SM (OH) C22:1 | 0.10 | 0.11 | 0.03 | 0.10 | 0.28 | 0.24 | 0.08 | 0.10 | 0.17 | 0.20 | 0.14 | 0.10 | 0.25 | 0.31 | 0.05 | 0.22 | 0.18 | 0.11 |
| SM (OH) C22:2 | 0.01 | 0.00 | 0.02 | 0.01 | 0.00 | 0.09 | 0.03 | 0.02 | 0.00 | 0.07 | 0.12 | 0.12 | 0.17 | 0.20 | 0.06 | 0.10 | 0.17 | 0.09 |
| SM (OH) C24:1 | 0.00 | 0.00 | 0.00 | 0.00 | 0.05 | 0.01 | 0.00 | 0.00 | 0.00 | 0.01 | 0.00 | 0.00 | 0.03 | 0.04 | 0.00 | 0.01 | 0.00 | 0.01 |
| SM C16:0 | 22.36 | 32.34 | 17.10 | 20.15 | 46.36 | 44.51 | 26.12 | 23.08 | 36.47 | 24.50 | 20.69 | 19.30 | 30.71 | 37.24 | 14.64 | 24.34 | 22.58 | 21.29 |
| SM C16:1 | 3.11 | 4.78 | 2.83 | 3.25 | 6.37 | 6.49 | 3.75 | 3.60 | 5.38 | 4.27 | 3.84 | 3.13 | 5.60 | 6.21 | 2.61 | 4.30 | 4.46 | 3.63 |
| SM C18:0 | 0.30 | 0.44 | 0.20 | 0.28 | 1.10 | 0.64 | 0.29 | 0.27 | 0.33 | 0.27 | 0.35 | 0.38 | 0.36 | 0.77 | 0.12 | 0.34 | 0.17 | 0.33 |
| SM C18:1 | 0.52 | 0.82 | 0.45 | 0.54 | 1.15 | 0.99 | 0.55 | 0.55 | 0.95 | 0.75 | 0.64 | 0.75 | 0.97 | 1.13 | 0.52 | 0.69 | 0.83 | 0.58 |
| SM C20:2 | 0.14 | 0.28 | 0.10 | 0.15 | 0.35 | 0.25 | 0.12 | 0.15 | 0.30 | 0.20 | 0.14 | 0.22 | 0.24 | 0.32 | 0.12 | 0.16 | 0.14 | 0.12 |
| SM C22:3 | 0.06 | 0.10 | 0.06 | 0.03 | 0.28 | 0.12 | 0.00 | 0.06 | 0.15 | 0.06 | 0.05 | 0.10 | 0.08 | 0.18 | 0.09 | 0.07 | 0.04 | 0.02 |
| SM C24:0 | 1.37 | 2.29 | 1.09 | 1.85 | 4.39 | 4.02 | 1.75 | 1.61 | 3.21 | 1.99 | 1.51 | 1.99 | 2.93 | 3.17 | 1.20 | 2.08 | 2.13 | 2.10 |
| SM C24:1 | 0.44 | 0.54 | 0.37 | 0.52 | 1.43 | 1.33 | 0.46 | 0.58 | 0.98 | 0.70 | 0.59 | 0.77 | 1.03 | 1.09 | 0.50 | 0.77 | 0.80 | 0.85 |
| SM C26:0 | 0.03 | 0.05 | 0.02 | 0.03 | 0.04 | 0.07 | 0.02 | 0.02 | 0.03 | 0.04 | 0.01 | 0.03 | 0.04 | 0.02 | 0.00 | 0.02 | 0.02 | 0.03 |
| SM C26:1 | 0.05 | 0.07 | 0.03 | 0.06 | 0.12 | 0.08 | 0.03 | 0.04 | 0.08 | 0.07 | 0.03 | 0.08 | 0.09 | 0.10 | 0.04 | 0.05 | 0.07 | 0.06 |
| C0 | 6.59 | 7.04 | 4.22 | 7.48 | 8.19 | 7.38 | 5.56 | 5.08 | 6.08 | 14.44 | 12.70 | 9.15 | 15.57 | 17.34 | 15.23 | 14.90 | 16.47 | 15.74 |
| C14 | 0.06 | 0.06 | 0.03 | 0.05 | 0.07 | 0.09 | 0.04 | 0.04 | 0.05 | 0.06 | 0.05 | 0.08 | 0.06 | 0.06 | 0.06 | 0.06 | 0.06 | 0.06 |
| C16 | 0.03 | 0.04 | 0.03 | 0.03 | 0.04 | 0.05 | 0.03 | 0.03 | 0.03 | 0.07 | 0.06 | 0.04 | 0.07 | 0.09 | 0.06 | 0.07 | 0.08 | 0.08 |
| C18 | 0.03 | 0.03 | 0.02 | 0.03 | 0.03 | 0.05 | 0.03 | 0.03 | 0.03 | 0.04 | 0.04 | 0.05 | 0.04 | 0.05 | 0.04 | 0.04 | 0.05 | 0.04 |
| C18:1 | 0.09 | 0.08 | 0.05 | 0.07 | 0.10 | 0.14 | 0.06 | 0.07 | 0.09 | 0.09 | 0.08 | 0.16 | 0.09 | 0.11 | 0.09 | 0.08 | 0.10 | 0.12 |
| C2 | 2.88 | 2.76 | 1.69 | 2.82 | 3.27 | 1.24 | 2.08 | 1.92 | 2.25 | 2.86 | 2.77 | 1.19 | 3.93 | 4.28 | 1.79 | 3.94 | 4.23 | 3.76 |
| C3 | 0.51 | 0.49 | 0.32 | 0.49 | 0.63 | 0.47 | 0.45 | 0.38 | 0.51 | 0.87 | 0.75 | 0.29 | 0.94 | 1.02 | 0.47 | 0.90 | 1.06 | 0.93 |
| C3-DC-M / C5-OH | 0.04 | 0.04 | 0.03 | 0.03 | 0.04 | 0.04 | 0.04 | 0.03 | 0.04 | 0.04 | 0.04 | 0.03 | 0.04 | 0.05 | 0.04 | 0.04 | 0.05 | 0.04 |
| C4 | 3.38 | 3.20 | 1.89 | 3.33 | 3.84 | 2.18 | 2.55 | 2.17 | 2.70 | 6.83 | 6.08 | 2.98 | 7.37 | 7.67 | 4.55 | 6.46 | 7.03 | 6.66 |
| C5 | 12.07 | 11.62 | 7.29 | 11.77 | 12.94 | 9.67 | 9.90 | 8.83 | 10.85 | 15.25 | 13.10 | 7.22 | 15.62 | 18.31 | 12.32 | 15.89 | 17.82 | 16.36 |
| C5-M-DC | 0.07 | 0.06 | 0.05 | 0.06 | 0.06 | 0.07 | 0.06 | 0.05 | 0.07 | 0.07 | 0.06 | 0.10 | 0.06 | 0.07 | 0.06 | 0.06 | 0.06 | 0.06 |
| C5:1 | 0.05 | 0.04 | 0.03 | 0.03 | 0.04 | 0.06 | 0.04 | 0.04 | 0.05 | 0.06 | 0.05 | 0.05 | 0.05 | 0.06 | 0.05 | 0.05 | 0.06 | 0.06 |
| C6 / C4:1-DC | 0.03 | 0.03 | 0.02 | 0.03 | 0.03 | 0.03 | 0.03 | 0.02 | 0.02 | 0.04 | 0.04 | 0.03 | 0.05 | 0.04 | 0.03 | 0.04 | 0.04 | 0.04 |

|  | **WT KRAS** | | | | | | | | | **G12C KRAS** | | | | | | | | |
| --- | --- | --- | --- | --- | --- | --- | --- | --- | --- | --- | --- | --- | --- | --- | --- | --- | --- | --- |
|  | **Untreated** | | | **BEZ235 24h** | | | **BKM120 24h** | | | **Untreated** | | | **BEZ235 24h** | | | **BKM120 24h** | | |
| **Metabolite** | **R1^a)^** | **R2^b)^** | **R3^c)^** | **R1^a)^** | **R2^b)^** | **R3^c)^** | **R1^a)^** | **R2^b)^** | **R3^c)^** | **R1^a)^** | **R2^b)^** | **R3^c)^** | **R1^a)^** | **R2^b)^** | **R3^c)^** | **R1^a)^** | **R2^b)^** | **R3^c)^** |
| lysoPC a C16:0 | 5.90 | 7.22 | 5.71 | 5.97 | 7.06 | 9.65 | 7.92 | 3.25 | 4.37 | 11.33 | 8.36 | 10.84 | 11.39 | 13.47 | 4.68 | 7.39 | 6.59 | 6.25 |
| lysoPC a C16:1 | 0.81 | 1.11 | 0.96 | 1.08 | 0.96 | 1.38 | 1.35 | 0.48 | 0.55 | 1.71 | 1.35 | 1.72 | 1.34 | 1.68 | 1.01 | 1.34 | 0.92 | 1.09 |
| lysoPC a C17:0 | 0.37 | 0.43 | 0.38 | 0.40 | 0.43 | 0.55 | 0.49 | 0.25 | 0.30 | 0.72 | 0.56 | 0.70 | 0.75 | 0.82 | 0.38 | 0.55 | 0.46 | 0.46 |
| lysoPC a C18:0 | 2.07 | 2.72 | 2.45 | 2.72 | 2.78 | 3.77 | 4.01 | 1.85 | 2.24 | 3.51 | 3.70 | 4.10 | 4.68 | 5.06 | 2.10 | 3.57 | 3.18 | 3.44 |
| lysoPC a C18:1 | 4.75 | 6.78 | 5.34 | 5.76 | 6.05 | 8.74 | 8.67 | 2.86 | 3.90 | 7.67 | 6.13 | 8.06 | 7.93 | 8.91 | 3.60 | 5.97 | 4.94 | 5.02 |
| lysoPC a C18:2 | 0.32 | 0.44 | 0.34 | 0.44 | 0.34 | 0.53 | 0.50 | 0.20 | 0.26 | 0.48 | 0.47 | 0.51 | 0.53 | 0.57 | 0.35 | 0.49 | 0.37 | 0.36 |
| lysoPC a C20:3 | 0.17 | 0.27 | 0.20 | 0.27 | 0.23 | 0.41 | 0.38 | 0.15 | 0.15 | 0.30 | 0.26 | 0.34 | 0.33 | 0.33 | 0.21 | 0.31 | 0.25 | 0.27 |
| lysoPC a C20:4 | 0.19 | 0.36 | 0.23 | 0.43 | 0.32 | 0.45 | 0.53 | 0.13 | 0.18 | 0.31 | 0.23 | 0.37 | 0.37 | 0.42 | 0.12 | 0.44 | 0.22 | 0.31 |
| lysoPC a C24:0 | 0.12 | 0.22 | 0.17 | 0.36 | 0.19 | 0.27 | 0.27 | 0.17 | 0.16 | 0.28 | 0.28 | 0.31 | 0.34 | 0.30 | 0.21 | 0.33 | 0.33 | 0.21 |
| lysoPC a C26:0 | 0.26 | 0.60 | 0.41 | 1.01 | 0.46 | 0.71 | 0.75 | 0.47 | 0.42 | 0.67 | 0.75 | 0.93 | 0.99 | 0.89 | 0.76 | 0.91 | 0.91 | 0.59 |
| lysoPC a C26:1 | 0.17 | 0.31 | 0.23 | 0.41 | 0.26 | 0.42 | 0.40 | 0.21 | 0.23 | 0.40 | 0.41 | 0.47 | 0.55 | 0.56 | 0.43 | 0.46 | 0.42 | 0.37 |
| lysoPC a C28:0 | 0.46 | 0.89 | 0.75 | 1.14 | 0.79 | 1.35 | 1.16 | 0.62 | 0.61 | 0.87 | 1.17 | 1.26 | 1.23 | 1.25 | 0.94 | 1.00 | 0.92 | 0.84 |
| lysoPC a C28:1 | 0.31 | 0.64 | 0.52 | 0.77 | 0.55 | 0.85 | 0.81 | 0.38 | 0.44 | 0.74 | 0.77 | 0.93 | 0.82 | 0.87 | 0.68 | 0.76 | 0.70 | 0.60 |
| PC aa C24:0 | 0.12 | 0.23 | 0.19 | 0.37 | 0.19 | 0.22 | 0.22 | 0.17 | 0.18 | 0.19 | 0.16 | 0.25 | 0.20 | 0.20 | 0.12 | 0.26 | 0.26 | 0.15 |
| PC aa C28:1 | 1.05 | 2.08 | 1.98 | 2.12 | 1.93 | 2.87 | 2.54 | 1.23 | 1.54 | 1.68 | 1.76 | 2.39 | 1.79 | 2.06 | 1.28 | 1.69 | 1.54 | 1.44 |
| PC aa C30:0 | 6.06 | 11.49 | 11.75 | 11.41 | 12.67 | 22.62 | 17.22 | 7.37 | 8.41 | 8.57 | 9.72 | 11.38 | 10.05 | 12.98 | 9.56 | 7.73 | 8.04 | 8.03 |
| PC aa C30:2 | 0.30 | 0.51 | 0.54 | 0.49 | 0.48 | 0.70 | 0.54 | 0.27 | 0.27 | 0.78 | 0.78 | 0.76 | 0.68 | 0.80 | 0.51 | 0.39 | 0.44 | 0.48 |
| PC aa C32:0 | 51.56 | 105.07 | 115.46 | 92.81 | 119.88 | 228.66 | 139.86 | 57.11 | 86.60 | 61.02 | 78.78 | 89.70 | 67.99 | 103.29 | 104.57 | 53.10 | 65.41 | 59.34 |
| PC aa C32:1 | 527.04 | 1188.41 | 1274.96 | 709.76 | 1111.98 | 1803.07 | 1166.41 | 471.38 | 602.71 | 772.73 | 941.13 | 1073.20 | 670.44 | 857.41 | 610.07 | 440.43 | 435.15 | 521.41 |
| PC aa C32:2 | 83.52 | 162.73 | 172.21 | 87.35 | 134.07 | 202.57 | 129.44 | 57.48 | 73.79 | 134.90 | 161.09 | 172.85 | 96.03 | 120.64 | 79.95 | 60.49 | 57.27 | 73.85 |
| PC aa C32:3 | 4.88 | 9.01 | 9.36 | 5.63 | 8.30 | 12.47 | 8.47 | 3.54 | 4.83 | 7.27 | 8.79 | 9.60 | 6.18 | 7.76 | 4.99 | 4.18 | 3.89 | 4.70 |
| PC aa C34:1 | 1041.31 | 2257.43 | 2346.00 | 1584.50 | 2221.76 | 3393.77 | 2188.21 | 1123.00 | 1550.48 | 1106.49 | 1380.30 | 1531.45 | 1115.50 | 1335.36 | 1250.89 | 837.43 | 882.02 | 854.44 |
| PC aa C34:2 | 387.52 | 842.86 | 932.75 | 486.35 | 753.56 | 1268.65 | 865.52 | 341.02 | 420.36 | 576.36 | 711.37 | 757.13 | 504.36 | 665.77 | 445.73 | 318.31 | 313.31 | 412.82 |
| PC aa C34:3 | 27.41 | 57.55 | 60.19 | 35.17 | 54.28 | 84.12 | 59.49 | 23.67 | 29.74 | 47.31 | 55.38 | 62.72 | 43.43 | 53.80 | 34.22 | 26.32 | 25.37 | 31.73 |
| PC aa C34:4 | 6.00 | 11.15 | 11.99 | 6.61 | 10.48 | 15.34 | 11.09 | 4.93 | 6.29 | 9.14 | 10.82 | 11.93 | 8.31 | 10.08 | 6.25 | 5.14 | 5.12 | 6.25 |
| PC aa C36:0 | 5.10 | 12.81 | 15.57 | 10.16 | 13.85 | 24.20 | 17.24 | 5.92 | 7.76 | 7.70 | 10.66 | 11.00 | 7.61 | 10.89 | 6.95 | 5.58 | 5.67 | 7.51 |
| PC aa C36:1 | 67.90 | 134.00 | 159.41 | 104.92 | 147.71 | 351.11 | 253.93 | 89.58 | 125.97 | 72.19 | 85.96 | 104.63 | 78.58 | 123.09 | 123.46 | 70.81 | 85.55 | 75.22 |
| PC aa C36:2 | 498.09 | 1104.33 | 1265.52 | 725.28 | 1080.47 | 2041.70 | 1383.45 | 514.56 | 634.04 | 539.59 | 703.18 | 774.72 | 528.15 | 775.35 | 577.81 | 378.96 | 399.06 | 464.90 |
| PC aa C36:3 | 78.45 | 178.66 | 194.90 | 113.88 | 179.67 | 341.54 | 235.59 | 87.24 | 109.66 | 112.51 | 140.33 | 151.77 | 119.91 | 158.50 | 108.93 | 81.99 | 79.97 | 100.55 |
| PC aa C36:4 | 36.57 | 84.39 | 93.65 | 57.82 | 87.35 | 145.61 | 107.96 | 42.94 | 52.16 | 57.13 | 73.62 | 79.76 | 63.42 | 82.65 | 55.15 | 42.85 | 44.37 | 51.77 |
| PC aa C36:5 | 17.20 | 37.40 | 39.73 | 24.36 | 37.00 | 60.17 | 45.18 | 18.59 | 22.47 | 28.55 | 35.15 | 38.13 | 31.50 | 39.15 | 25.08 | 21.32 | 19.74 | 23.61 |
| PC aa C36:6 | 7.11 | 15.82 | 17.94 | 9.60 | 15.13 | 29.16 | 18.68 | 6.77 | 9.20 | 9.25 | 11.36 | 12.87 | 8.77 | 11.67 | 7.87 | 5.80 | 5.73 | 6.81 |
| PC aa C38:0 | 3.69 | 8.74 | 8.95 | 5.76 | 8.99 | 15.41 | 12.72 | 4.60 | 5.78 | 5.70 | 7.81 | 8.20 | 5.97 | 8.15 | 6.10 | 4.55 | 4.58 | 5.46 |
| PC aa C38:1 | 0.04 | 0.04 | 0.04 | 0.17 | 0.59 | 1.11 | 0.33 | 0.29 | 0.04 | 0.16 | 0.69 | 0.83 | 0.46 | 1.04 | 1.40 | 0.45 | 0.78 | 0.59 |
| PC aa C38:3 | 10.30 | 22.57 | 23.95 | 17.28 | 24.90 | 49.59 | 34.47 | 12.56 | 16.56 | 14.09 | 18.05 | 20.52 | 15.78 | 23.67 | 19.11 | 11.86 | 13.27 | 14.12 |
| PC aa C38:4 | 17.42 | 40.71 | 44.16 | 30.97 | 43.56 | 81.80 | 63.49 | 24.00 | 29.19 | 26.68 | 36.13 | 37.84 | 31.71 | 44.30 | 32.69 | 23.03 | 26.10 | 28.33 |
| PC aa C38:5 | 28.41 | 62.80 | 67.88 | 45.42 | 65.41 | 111.78 | 87.58 | 35.30 | 41.15 | 41.07 | 51.91 | 53.16 | 45.03 | 57.43 | 39.59 | 32.40 | 33.79 | 38.09 |
| PC aa C38:6 | 25.41 | 55.88 | 60.12 | 39.31 | 61.13 | 99.83 | 80.36 | 31.89 | 37.33 | 34.61 | 44.49 | 48.15 | 41.17 | 53.55 | 34.41 | 28.38 | 28.83 | 34.49 |
| PC aa C40:2 | 1.16 | 2.26 | 2.64 | 2.16 | 2.45 | 4.99 | 3.76 | 1.49 | 1.89 | 1.58 | 2.21 | 2.28 | 1.49 | 2.02 | 2.30 | 1.36 | 1.62 | 1.33 |
| PC aa C40:3 | 0.84 | 1.89 | 2.13 | 1.56 | 2.03 | 4.33 | 2.70 | 1.20 | 1.36 | 1.28 | 1.72 | 1.79 | 1.22 | 1.71 | 1.80 | 1.06 | 1.31 | 1.22 |
| PC aa C40:4 | 1.92 | 4.17 | 3.87 | 3.13 | 3.97 | 8.12 | 6.66 | 2.29 | 3.00 | 2.31 | 3.05 | 3.30 | 2.64 | 3.57 | 3.26 | 2.28 | 2.21 | 2.46 |
| PC aa C40:5 | 6.37 | 14.02 | 15.65 | 11.94 | 15.74 | 30.29 | 25.62 | 8.80 | 10.21 | 8.30 | 9.95 | 10.64 | 8.76 | 13.35 | 10.98 | 7.02 | 8.36 | 8.44 |
| PC aa C40:6 | 10.00 | 21.87 | 23.10 | 17.59 | 22.21 | 43.38 | 33.35 | 12.68 | 15.26 | 13.75 | 18.96 | 20.45 | 16.84 | 23.36 | 16.20 | 12.69 | 13.08 | 14.84 |
| PC aa C42:0 | 0.29 | 0.60 | 0.51 | 0.46 | 0.55 | 0.98 | 0.62 | 0.31 | 0.28 | 0.42 | 0.44 | 0.53 | 0.46 | 0.52 | 0.61 | 0.42 | 0.35 | 0.37 |
| PC aa C42:1 | 0.41 | 0.58 | 0.62 | 0.47 | 0.51 | 1.07 | 0.80 | 0.38 | 0.40 | 0.52 | 0.72 | 0.60 | 0.46 | 0.78 | 1.13 | 0.64 | 0.69 | 0.62 |
| PC aa C42:2 | 0.41 | 0.55 | 0.76 | 0.63 | 0.84 | 1.73 | 1.12 | 0.48 | 0.59 | 0.69 | 1.00 | 0.90 | 0.63 | 0.90 | 1.27 | 0.62 | 0.82 | 0.60 |
| PC aa C42:4 | 0.47 | 0.85 | 0.59 | 0.69 | 0.73 | 1.55 | 1.08 | 0.52 | 0.69 | 0.52 | 0.59 | 0.72 | 0.52 | 0.67 | 0.77 | 0.46 | 0.47 | 0.47 |
| PC aa C42:5 | 0.98 | 2.13 | 2.39 | 1.68 | 2.48 | 5.40 | 3.74 | 1.20 | 1.64 | 1.24 | 1.67 | 1.60 | 1.30 | 1.95 | 2.13 | 1.04 | 1.27 | 1.28 |
| PC aa C42:6 | 2.97 | 6.37 | 6.49 | 4.95 | 6.44 | 13.85 | 10.75 | 3.48 | 4.34 | 3.28 | 3.85 | 4.60 | 4.15 | 5.49 | 4.39 | 2.96 | 3.10 | 3.21 |
| PC ae C30:0 | 1.18 | 2.30 | 2.38 | 2.31 | 2.43 | 4.40 | 3.21 | 1.37 | 1.54 | 2.29 | 2.54 | 3.03 | 2.46 | 3.22 | 2.44 | 1.87 | 1.86 | 1.97 |
| PC ae C30:1 | 0.63 | 1.15 | 1.26 | 1.14 | 1.33 | 2.43 | 2.03 | 0.79 | 0.89 | 1.23 | 1.23 | 1.54 | 1.20 | 1.60 | 1.10 | 0.88 | 0.89 | 1.01 |
| PC ae C30:2 | 0.12 | 0.19 | 0.20 | 0.18 | 0.19 | 0.31 | 0.22 | 0.11 | 0.12 | 0.22 | 0.23 | 0.25 | 0.21 | 0.23 | 0.17 | 0.13 | 0.15 | 0.15 |
| PC ae C32:1 | 76.18 | 176.72 | 189.69 | 103.13 | 150.39 | 259.20 | 176.01 | 68.63 | 84.80 | 134.42 | 165.83 | 192.67 | 106.42 | 158.79 | 108.57 | 71.75 | 72.61 | 90.07 |
| PC ae C32:2 | 20.63 | 41.24 | 44.80 | 25.39 | 39.77 | 65.37 | 44.50 | 18.78 | 23.71 | 27.23 | 33.25 | 37.77 | 23.34 | 31.60 | 21.72 | 15.72 | 15.99 | 19.29 |
| PC ae C34:0 | 11.68 | 24.87 | 22.97 | 21.13 | 25.14 | 43.15 | 29.74 | 13.24 | 17.16 | 19.41 | 24.20 | 25.87 | 20.52 | 27.08 | 33.30 | 15.60 | 18.78 | 17.85 |
| PC ae C34:1 | 170.96 | 363.86 | 431.20 | 246.90 | 360.76 | 701.89 | 455.18 | 161.01 | 222.53 | 232.72 | 281.55 | 321.64 | 216.83 | 323.99 | 244.07 | 150.20 | 162.00 | 181.69 |
| PC ae C34:2 | 75.69 | 151.28 | 174.19 | 94.27 | 148.85 | 269.39 | 181.05 | 68.90 | 87.78 | 96.56 | 114.55 | 131.18 | 84.44 | 120.77 | 91.30 | 56.97 | 58.53 | 68.80 |
| PC ae C34:3 | 10.54 | 22.81 | 24.56 | 13.85 | 21.63 | 38.95 | 24.43 | 10.02 | 12.88 | 13.08 | 16.01 | 18.43 | 11.63 | 16.13 | 11.51 | 7.69 | 7.86 | 9.46 |
| PC ae C36:0 | 6.34 | 12.24 | 14.04 | 7.33 | 11.10 | 18.05 | 12.46 | 4.86 | 6.43 | 7.37 | 9.09 | 9.39 | 6.77 | 8.29 | 6.84 | 4.63 | 4.90 | 5.73 |
| PC ae C36:1 | 39.22 | 81.65 | 89.92 | 61.53 | 86.23 | 171.99 | 116.58 | 41.87 | 60.91 | 48.00 | 59.91 | 69.54 | 51.71 | 75.94 | 62.72 | 39.36 | 43.58 | 43.34 |
| PC ae C36:2 | 70.97 | 155.18 | 173.76 | 99.36 | 155.75 | 295.78 | 188.19 | 70.30 | 94.60 | 92.07 | 115.11 | 125.28 | 90.79 | 131.50 | 94.36 | 61.67 | 63.86 | 75.25 |
| PC ae C36:3 | 29.68 | 63.99 | 71.60 | 42.59 | 63.56 | 127.69 | 76.09 | 26.14 | 35.45 | 37.37 | 48.70 | 52.83 | 36.51 | 49.06 | 34.93 | 23.10 | 23.53 | 27.34 |
| PC ae C36:4 | 18.69 | 41.56 | 44.76 | 27.61 | 39.96 | 73.91 | 54.68 | 20.02 | 23.80 | 31.17 | 37.15 | 42.63 | 30.15 | 42.29 | 28.48 | 20.67 | 21.16 | 24.64 |
| PC ae C36:5 | 11.93 | 25.20 | 28.36 | 17.40 | 26.31 | 44.07 | 35.38 | 13.06 | 15.27 | 20.70 | 25.47 | 28.60 | 21.13 | 28.25 | 18.96 | 14.13 | 14.19 | 17.28 |
| PC ae C38:0 | 5.07 | 10.44 | 10.85 | 6.13 | 9.32 | 15.95 | 11.72 | 4.67 | 5.78 | 7.62 | 9.03 | 9.52 | 6.99 | 8.88 | 6.23 | 4.88 | 4.78 | 6.10 |
| PC ae C38:1 | 2.40 | 5.17 | 5.93 | 4.19 | 5.37 | 11.88 | 7.65 | 2.95 | 3.92 | 3.61 | 4.49 | 4.82 | 3.39 | 5.26 | 4.91 | 2.94 | 3.42 | 2.98 |
| PC ae C38:2 | 13.13 | 25.58 | 29.54 | 18.17 | 25.31 | 52.61 | 34.12 | 13.58 | 17.47 | 15.13 | 19.31 | 21.32 | 14.92 | 22.19 | 18.04 | 11.50 | 12.01 | 12.86 |
| PC ae C38:3 | 7.72 | 17.72 | 20.31 | 12.96 | 18.37 | 42.68 | 25.80 | 8.42 | 11.63 | 9.82 | 13.58 | 15.01 | 10.41 | 14.98 | 11.90 | 7.53 | 7.94 | 9.02 |
| PC ae C38:4 | 9.54 | 20.87 | 22.44 | 15.64 | 22.82 | 43.50 | 31.87 | 10.95 | 14.38 | 14.04 | 17.61 | 19.15 | 14.97 | 21.53 | 16.02 | 10.55 | 11.41 | 12.92 |
| PC ae C38:5 | 13.36 | 30.58 | 34.64 | 22.09 | 31.92 | 59.89 | 44.10 | 16.95 | 20.25 | 23.55 | 28.55 | 33.86 | 23.68 | 33.24 | 23.04 | 17.98 | 18.11 | 21.02 |
| PC ae C38:6 | 12.70 | 29.57 | 33.70 | 18.07 | 29.47 | 52.98 | 40.15 | 14.43 | 17.71 | 23.51 | 27.87 | 31.90 | 20.92 | 31.82 | 21.29 | 15.28 | 16.34 | 18.80 |
| PC ae C40:1 | 1.12 | 2.00 | 1.85 | 1.57 | 2.27 | 3.67 | 2.79 | 0.89 | 1.25 | 1.64 | 1.91 | 1.96 | 1.55 | 2.16 | 1.54 | 1.14 | 1.28 | 1.53 |
| PC ae C40:2 | 3.19 | 5.20 | 5.06 | 3.91 | 4.90 | 7.79 | 5.37 | 3.39 | 4.54 | 2.92 | 3.52 | 3.46 | 2.59 | 3.52 | 3.39 | 2.24 | 2.50 | 2.25 |
| PC ae C40:3 | 1.38 | 2.68 | 3.02 | 2.25 | 2.51 | 5.32 | 3.51 | 1.64 | 2.06 | 1.85 | 2.29 | 2.52 | 1.95 | 2.53 | 2.28 | 1.56 | 1.57 | 1.64 |
| PC ae C40:4 | 1.82 | 3.86 | 4.07 | 2.69 | 4.08 | 8.61 | 5.85 | 2.05 | 2.46 | 2.62 | 3.47 | 3.56 | 2.60 | 4.09 | 3.39 | 2.28 | 2.50 | 2.63 |
| PC ae C40:5 | 5.12 | 11.63 | 12.93 | 8.30 | 11.69 | 23.47 | 15.86 | 6.24 | 7.75 | 8.15 | 10.31 | 11.46 | 7.97 | 12.55 | 9.54 | 6.19 | 6.96 | 7.07 |
| PC ae C40:6 | 4.60 | 9.77 | 10.90 | 7.40 | 10.04 | 20.19 | 14.38 | 5.34 | 6.82 | 7.30 | 8.95 | 10.35 | 7.48 | 10.32 | 8.42 | 5.46 | 6.31 | 6.62 |
| PC ae C42:0 | 1.31 | 2.41 | 2.32 | 2.09 | 2.47 | 4.65 | 3.20 | 1.66 | 1.74 | 1.62 | 2.02 | 1.96 | 1.82 | 2.41 | 1.82 | 1.26 | 1.36 | 1.55 |
| PC ae C42:2 | 0.62 | 1.05 | 1.06 | 0.81 | 1.12 | 1.80 | 1.40 | 0.55 | 0.79 | 0.78 | 0.78 | 0.89 | 0.79 | 1.03 | 0.82 | 0.59 | 0.68 | 0.72 |
| PC ae C42:3 | 0.71 | 1.27 | 1.20 | 1.01 | 1.28 | 2.49 | 1.76 | 0.74 | 0.83 | 0.90 | 1.21 | 1.07 | 0.91 | 1.20 | 1.06 | 0.74 | 0.71 | 0.77 |
| PC ae C42:4 | 0.58 | 1.04 | 1.13 | 0.89 | 1.28 | 2.11 | 1.17 | 0.57 | 0.83 | 0.67 | 1.11 | 0.92 | 0.77 | 0.89 | 0.80 | 0.61 | 0.61 | 0.65 |
| PC ae C42:5 | 1.49 | 2.26 | 2.40 | 2.02 | 2.44 | 4.90 | 3.94 | 1.60 | 1.92 | 1.50 | 2.00 | 2.06 | 1.85 | 2.26 | 2.09 | 1.41 | 1.45 | 1.49 |
| PC ae C44:3 | 0.24 | 0.46 | 0.41 | 0.53 | 0.66 | 0.95 | 0.67 | 0.45 | 0.36 | 0.40 | 0.56 | 0.48 | 0.40 | 0.45 | 0.49 | 0.30 | 0.36 | 0.37 |
| PC ae C44:4 | 0.31 | 0.51 | 0.57 | 0.44 | 0.46 | 0.76 | 0.63 | 0.13 | 0.38 | 0.28 | 0.46 | 0.37 | 0.36 | 0.44 | 0.33 | 0.25 | 0.28 | 0.31 |
| PC ae C44:5 | 0.36 | 0.51 | 0.65 | 0.52 | 0.59 | 1.03 | 0.80 | 0.41 | 0.45 | 0.35 | 0.55 | 0.47 | 0.43 | 0.49 | 0.47 | 0.34 | 0.36 | 0.32 |
| PC ae C44:6 | 0.32 | 0.66 | 0.65 | 0.48 | 0.70 | 1.15 | 0.80 | 0.31 | 0.36 | 0.42 | 0.51 | 0.57 | 0.40 | 0.64 | 0.53 | 0.31 | 0.40 | 0.38 |
| Sugars | 4281.02 | 3038.59 | 2997.45 | 3653.88 | 3215.25 | 2588.45 | 2165.67 | 1923.04 | 1908.60 | 5304.41 | 3895.19 | 3755.51 | 2612.49 | 2819.02 | 3226.10 | 1537.38 | 1990.75 | 1293.30 |
| Ala | 353.33 | 380.00 | 386.67 | 580.00 | 516.67 | 486.67 | 360.00 | 250.33 | 346.67 | 1466.67 | 1123.33 | 1350.00 | 1450.00 | 1590.00 | 1530.00 | 836.67 | 926.67 | 810.00 |
| Arg | 147.67 | 126.00 | 107.67 | 148.33 | 126.67 | 128.33 | 82.67 | 79.67 | 81.67 | 167.00 | 165.33 | 132.00 | 148.00 | 140.33 | 183.33 | 132.33 | 126.67 | 79.33 |
| Asn | 790.00 | 843.33 | 846.67 | 1166.67 | 1026.67 | 1020.00 | 656.67 | 486.67 | 703.33 | 980.00 | 886.67 | 1183.33 | 1153.33 | 1163.33 | 1233.33 | 733.33 | 850.00 | 600.00 |
| Asp | 164.67 | 181.00 | 172.67 | 236.67 | 233.33 | 218.00 | 141.00 | 140.00 | 178.00 | 253.33 | 239.33 | 272.00 | 244.00 | 265.00 | 259.00 | 190.33 | 229.00 | 162.33 |
| Cit | 4.67 | 5.50 | 5.90 | 7.40 | 5.17 | 5.77 | 4.43 | 2.85 | 4.73 | 7.10 | 6.00 | 6.73 | 7.37 | 8.33 | 6.57 | 4.07 | 4.73 | 4.07 |
| Gln | 1136.67 | 1430.00 | 1076.67 | 1946.67 | 1803.33 | 1810.00 | 1316.67 | 906.67 | 1206.67 | 556.67 | 526.67 | 490.00 | 1040.00 | 1046.67 | 1160.00 | 890.00 | 1036.67 | 703.33 |
| Glu | 4133.33 | 5166.67 | 4833.33 | 4733.33 | 4166.67 | 4533.33 | 3146.67 | 2636.67 | 3333.33 | 3566.67 | 2810.00 | 3600.00 | 3366.67 | 3900.00 | 3733.33 | 2570.00 | 2616.67 | 2090.00 |
| Gly | 543.33 | 616.67 | 536.67 | 743.33 | 700.00 | 763.33 | 556.67 | 410.00 | 550.00 | 483.33 | 383.33 | 440.00 | 596.67 | 606.67 | 650.00 | 493.33 | 556.67 | 370.00 |
| His | 41.33 | 44.00 | 36.67 | 47.00 | 45.67 | 46.67 | 34.67 | 24.47 | 32.90 | 53.67 | 43.67 | 45.00 | 52.00 | 53.00 | 58.67 | 41.33 | 44.00 | 33.67 |
| Ile | 145.00 | 141.33 | 123.67 | 148.33 | 144.33 | 148.00 | 106.67 | 81.00 | 101.00 | 211.00 | 178.00 | 178.00 | 196.67 | 193.33 | 214.33 | 140.67 | 166.00 | 124.00 |
| Leu | 136.33 | 135.67 | 119.00 | 143.00 | 141.00 | 147.67 | 105.67 | 100.67 | 99.33 | 217.33 | 183.67 | 174.00 | 190.00 | 207.33 | 226.00 | 159.33 | 178.33 | 140.33 |
| Lys | 15.03 | 9.63 | 8.90 | 17.30 | 13.33 | 11.00 | 9.07 | 8.13 | 8.33 | 16.27 | 14.77 | 8.23 | 13.63 | 12.10 | 21.67 | 15.63 | 15.13 | 8.23 |
| Met | 31.27 | 33.30 | 27.97 | 38.00 | 37.33 | 37.67 | 28.73 | 19.57 | 26.03 | 36.67 | 33.67 | 34.33 | 45.33 | 40.33 | 45.67 | 30.93 | 36.67 | 28.57 |
| Orn | 10.83 | 8.43 | 6.47 | 12.20 | 8.60 | 8.53 | 5.23 | 5.10 | 5.17 | 11.70 | 10.63 | 7.83 | 9.77 | 8.30 | 11.63 | 7.40 | 7.03 | 3.97 |
| Phe | 32.87 | 32.33 | 30.27 | 38.67 | 37.33 | 37.00 | 30.80 | 21.83 | 28.43 | 41.00 | 35.00 | 34.67 | 43.33 | 44.67 | 47.33 | 34.33 | 40.33 | 29.27 |
| Pro | 309.33 | 353.33 | 291.33 | 326.00 | 311.67 | 326.67 | 235.67 | 177.33 | 235.67 | 380.00 | 363.33 | 370.00 | 366.67 | 386.67 | 393.33 | 274.33 | 321.67 | 248.33 |
| Ser | 64.33 | 74.33 | 68.67 | 114.67 | 106.33 | 108.33 | 92.00 | 77.00 | 96.33 | 90.00 | 84.33 | 81.00 | 127.67 | 125.33 | 142.00 | 122.00 | 133.67 | 88.00 |
| Thr | 117.00 | 119.67 | 130.67 | 177.67 | 160.00 | 143.33 | 104.67 | 86.00 | 121.67 | 154.33 | 126.33 | 154.67 | 215.33 | 203.67 | 203.67 | 126.67 | 160.33 | 93.33 |
| Trp | 10.43 | 10.60 | 9.33 | 12.37 | 11.80 | 12.00 | 9.63 | 7.20 | 8.53 | 13.70 | 11.47 | 11.47 | 14.77 | 14.73 | 15.30 | 11.07 | 12.87 | 8.87 |
| Tyr | 50.33 | 50.33 | 46.00 | 55.33 | 53.33 | 56.67 | 39.33 | 31.90 | 41.00 | 64.33 | 53.33 | 55.33 | 63.00 | 67.00 | 71.33 | 47.33 | 55.33 | 42.00 |
| Val | 40.00 | 38.33 | 36.33 | 49.33 | 42.67 | 45.33 | 37.67 | 25.60 | 33.03 | 48.67 | 42.67 | 42.67 | 54.67 | 54.00 | 62.67 | 44.00 | 49.00 | 37.67 |
| Ac-Orn | 1.63 | 1.59 | 1.37 | 2.41 | 1.92 | 1.81 | 1.00 | 0.70 | 1.13 | 2.17 | 1.77 | 1.91 | 3.03 | 2.03 | 2.90 | 1.64 | 1.51 | 1.23 |
| ADMA | 0.17 | 0.19 | 0.18 | 0.17 | 0.40 | 0.14 | 0.27 | 0.22 | 0.13 | 0.26 | 0.61 | 0.24 | 0.34 | 0.31 | 0.49 | 0.24 | 0.21 | 0.24 |
| alpha-AAA | 2.76 | 3.47 | 2.20 | 2.76 | 2.39 | 2.81 | 1.74 | 1.03 | 1.77 | 3.25 | 2.55 | 2.72 | 2.83 | 3.04 | 4.07 | 2.32 | 2.28 | 1.58 |
| Carnosine | 0.66 | 0.49 | 0.50 | 0.60 | 0.56 | 0.55 | 0.42 | 0.28 | 0.36 | 0.68 | 0.57 | 0.38 | 0.33 | 0.39 | 0.51 | 0.26 | 0.46 | 0.24 |
| DOPA | 0.07 | 0.07 | 0.09 | 0.09 | 0.09 | 0.09 | 0.07 | 0.07 | 0.07 | 0.19 | 0.20 | 0.21 | 0.22 | 0.24 | 0.20 | 0.10 | 0.14 | 0.15 |
| Kynurenine | 0.53 | 0.53 | 0.47 | 0.52 | 0.41 | 0.49 | 0.36 | 0.29 | 0.37 | 0.65 | 0.59 | 0.61 | 0.63 | 0.67 | 0.69 | 0.46 | 0.53 | 0.39 |
| Met-SO | 1.92 | 1.63 | 1.69 | 1.93 | 1.87 | 1.86 | 1.43 | 0.92 | 1.12 | 1.89 | 1.87 | 2.33 | 2.03 | 1.99 | 2.43 | 1.65 | 1.84 | 1.42 |
| Putrescine | 41.00 | 27.60 | 27.23 | 57.00 | 38.00 | 40.00 | 21.00 | 22.43 | 24.23 | 38.67 | 36.67 | 27.93 | 53.00 | 34.00 | 51.33 | 25.93 | 25.17 | 12.97 |
| Serotonin | 0.20 | 0.19 | 0.17 | 0.17 | 0.16 | 0.15 | 0.13 | 0.08 | 0.12 | 0.11 | 0.07 | 0.06 | 0.06 | 0.07 | 0.07 | 0.04 | 0.06 | 0.04 |
| Spermidine | 7.33 | 4.90 | 8.50 | 8.70 | 7.70 | 4.77 | 3.40 | 3.60 | 4.80 | 9.37 | 8.40 | 7.43 | 8.23 | 9.00 | 14.87 | 6.57 | 7.10 | 3.37 |
| Spermine | 5.13 | 3.43 | 7.57 | 4.97 | 6.13 | 2.92 | 2.02 | 2.23 | 3.60 | 5.83 | 4.23 | 4.73 | 3.73 | 5.83 | 9.63 | 3.83 | 5.23 | 1.81 |
| t4-OH-Pro | 263.00 | 286.33 | 249.67 | 336.67 | 315.33 | 332.33 | 209.33 | 146.00 | 220.00 | 426.67 | 353.33 | 396.67 | 440.00 | 463.33 | 456.67 | 262.33 | 312.67 | 239.33 |
| Taurine | 222.33 | 229.33 | 218.67 | 210.00 | 200.67 | 202.67 | 180.00 | 161.33 | 188.00 | 267.33 | 251.33 | 257.00 | 254.33 | 241.67 | 248.67 | 213.33 | 218.00 | 196.67 |
| total DMA | 0.22 | 0.21 | 0.18 | 0.27 | 0.23 | 0.20 | 0.16 | 0.13 | 0.18 | 0.32 | 0.25 | 0.22 | 0.25 | 0.24 | 0.33 | 0.17 | 0.20 | 0.15 |
| SM (OH) C14:1 | 0.93 | 1.82 | 1.54 | 1.84 | 1.76 | 3.24 | 2.47 | 1.11 | 1.19 | 1.61 | 2.05 | 2.14 | 2.17 | 2.54 | 1.69 | 1.45 | 1.44 | 1.48 |
| SM (OH) C16:1 | 0.20 | 0.39 | 0.27 | 0.33 | 0.34 | 0.68 | 0.46 | 0.22 | 0.27 | 0.28 | 0.25 | 0.41 | 0.38 | 0.53 | 0.44 | 0.28 | 0.27 | 0.28 |
| SM (OH) C22:1 | 0.02 | 0.09 | 0.02 | 0.13 | 0.11 | 0.17 | 0.13 | 0.04 | 0.03 | 0.09 | 0.11 | 0.10 | 0.16 | 0.13 | 0.20 | 0.08 | 0.10 | 0.08 |
| SM (OH) C22:2 | 0.05 | 0.03 | 0.03 | 0.09 | 0.06 | 0.09 | 0.15 | 0.07 | 0.05 | 0.12 | 0.12 | 0.10 | 0.11 | 0.20 | 0.20 | 0.09 | 0.10 | 0.07 |
| SM (OH) C24:1 | 0.00 | 0.00 | 0.00 | 0.00 | 0.00 | 0.03 | 0.01 | 0.00 | 0.00 | 0.00 | 0.00 | 0.00 | 0.02 | 0.01 | 0.00 | 0.00 | 0.00 | 0.00 |
| SM C16:0 | 12.78 | 23.96 | 22.24 | 26.52 | 25.51 | 50.96 | 40.16 | 16.45 | 17.43 | 16.75 | 19.78 | 22.81 | 23.28 | 28.03 | 19.95 | 15.03 | 16.05 | 15.92 |
| SM C16:1 | 2.28 | 3.90 | 3.55 | 3.58 | 3.84 | 6.25 | 5.80 | 2.55 | 2.48 | 3.47 | 4.15 | 4.39 | 4.41 | 4.95 | 3.08 | 2.90 | 2.78 | 3.11 |
| SM C18:0 | 0.17 | 0.19 | 0.34 | 0.35 | 0.45 | 0.86 | 0.64 | 0.23 | 0.27 | 0.17 | 0.05 | 0.17 | 0.13 | 0.66 | 0.33 | 0.24 | 0.19 | 0.36 |
| SM C18:1 | 0.39 | 0.63 | 0.56 | 0.69 | 0.61 | 1.19 | 0.87 | 0.44 | 0.41 | 0.61 | 0.77 | 0.66 | 0.76 | 0.79 | 0.61 | 0.48 | 0.44 | 0.47 |
| SM C20:2 | 0.10 | 0.18 | 0.14 | 0.19 | 0.15 | 0.38 | 0.21 | 0.08 | 0.09 | 0.13 | 0.22 | 0.10 | 0.15 | 0.16 | 0.11 | 0.09 | 0.08 | 0.09 |
| SM C22:3 | 0.08 | 0.03 | 0.08 | 0.13 | 0.07 | 0.17 | 0.09 | 0.05 | 0.01 | 0.06 | 0.17 | 0.00 | 0.06 | 0.00 | 0.01 | 0.02 | 0.10 | 0.09 |
| SM C24:0 | 0.80 | 1.38 | 1.39 | 1.72 | 1.46 | 3.73 | 2.84 | 1.09 | 1.16 | 1.32 | 1.67 | 1.60 | 1.64 | 2.10 | 2.09 | 1.29 | 1.45 | 1.26 |
| SM C24:1 | 0.36 | 0.43 | 0.44 | 0.58 | 0.45 | 0.99 | 0.84 | 0.36 | 0.37 | 0.51 | 0.71 | 0.57 | 0.59 | 0.76 | 0.94 | 0.58 | 0.62 | 0.44 |
| SM C26:0 | 0.02 | 0.02 | 0.02 | 0.02 | 0.04 | 0.07 | 0.02 | 0.01 | 0.02 | 0.02 | 0.01 | 0.01 | 0.02 | 0.03 | 0.04 | 0.01 | 0.01 | 0.03 |
| SM C26:1 | 0.05 | 0.06 | 0.04 | 0.07 | 0.04 | 0.10 | 0.07 | 0.03 | 0.04 | 0.05 | 0.06 | 0.05 | 0.05 | 0.07 | 0.09 | 0.04 | 0.03 | 0.04 |
| C0 | 6.63 | 7.47 | 6.53 | 7.43 | 7.22 | 7.43 | 6.44 | 4.56 | 5.52 | 23.46 | 18.46 | 20.18 | 19.53 | 20.27 | 21.17 | 14.99 | 17.19 | 13.74 |
| C14 | 0.06 | 0.06 | 0.05 | 0.06 | 0.07 | 0.09 | 0.06 | 0.04 | 0.05 | 0.08 | 0.07 | 0.07 | 0.08 | 0.07 | 0.07 | 0.06 | 0.06 | 0.05 |
| C16 | 0.02 | 0.03 | 0.03 | 0.03 | 0.03 | 0.04 | 0.04 | 0.02 | 0.03 | 0.09 | 0.08 | 0.09 | 0.09 | 0.12 | 0.07 | 0.12 | 0.10 | 0.11 |
| C18 | 0.03 | 0.03 | 0.03 | 0.03 | 0.03 | 0.04 | 0.04 | 0.02 | 0.03 | 0.05 | 0.04 | 0.05 | 0.05 | 0.06 | 0.04 | 0.06 | 0.06 | 0.06 |
| C18:1 | 0.10 | 0.10 | 0.10 | 0.08 | 0.10 | 0.12 | 0.10 | 0.06 | 0.10 | 0.12 | 0.11 | 0.12 | 0.13 | 0.13 | 0.11 | 0.13 | 0.13 | 0.13 |
| C2 | 3.51 | 3.70 | 3.13 | 3.30 | 3.09 | 3.49 | 2.31 | 1.61 | 2.05 | 5.13 | 4.53 | 4.68 | 5.83 | 5.66 | 5.90 | 4.08 | 4.42 | 3.73 |
| C3 | 0.58 | 0.57 | 0.53 | 0.63 | 0.62 | 0.67 | 0.46 | 0.33 | 0.41 | 1.37 | 1.23 | 1.32 | 1.41 | 1.34 | 1.33 | 1.00 | 1.08 | 0.96 |
| C3-DC-M / C5-OH | 0.04 | 0.04 | 0.03 | 0.04 | 0.04 | 0.04 | 0.04 | 0.03 | 0.03 | 0.06 | 0.05 | 0.05 | 0.05 | 0.06 | 0.06 | 0.04 | 0.05 | 0.04 |
| C4 | 3.67 | 3.68 | 3.34 | 3.41 | 3.27 | 3.79 | 2.27 | 1.56 | 2.28 | 11.25 | 9.06 | 9.57 | 8.13 | 8.96 | 8.71 | 5.62 | 6.35 | 5.21 |
| C5 | 12.72 | 12.98 | 12.26 | 10.55 | 10.89 | 12.14 | 9.74 | 6.81 | 9.25 | 29.19 | 23.14 | 25.93 | 18.89 | 22.05 | 20.43 | 15.17 | 16.84 | 13.81 |
| C5-M-DC | 0.09 | 0.07 | 0.08 | 0.07 | 0.07 | 0.08 | 0.07 | 0.05 | 0.07 | 0.08 | 0.09 | 0.08 | 0.09 | 0.07 | 0.07 | 0.05 | 0.07 | 0.06 |
| C5:1 | 0.06 | 0.06 | 0.04 | 0.05 | 0.05 | 0.06 | 0.04 | 0.04 | 0.04 | 0.08 | 0.07 | 0.06 | 0.08 | 0.07 | 0.07 | 0.06 | 0.07 | 0.05 |
| C6 / C4:1-DC | 0.03 | 0.03 | 0.03 | 0.03 | 0.02 | 0.03 | 0.02 | 0.02 | 0.02 | 0.06 | 0.06 | 0.05 | 0.05 | 0.05 | 0.05 | 0.03 | 0.04 | 0.03 |

|  | **WT KRAS** | | | | | | | | | **G12C KRAS** | | | | | | | | |
| --- | --- | --- | --- | --- | --- | --- | --- | --- | --- | --- | --- | --- | --- | --- | --- | --- | --- | --- |
|  | **Untreated** | | | **BEZ235 48h** | | | **BKM120 48h** | | | **Untreated** | | | **BEZ235 48h** | | | **BKM120 48h** | | |
| **Metabolite** | **R1^a)^** | **R2^b)^** | **R3^c)^** | **R1^a)^** | **R2^b)^** | **R3^c)^** | **R1^a)^** | **R2^b)^** | **R3^c)^** | **R1^a)^** | **R2^b)^** | **R3^c)^** | **R1^a)^** | **R2^b)^** | **R3^c)^** | **R1^a)^** | **R2^b)^** | **R3^c)^** |
| lysoPC a C16:0 | 2.85 | 7.00 | 10.58 | 5.25 | 5.43 | 5.90 | 5.33 | 6.47 | 5.24 | 7.96 | 6.92 | 6.78 | 10.01 | 7.98 | 6.14 | 9.03 | 4.68 | 8.39 |
| lysoPC a C16:1 | 0.54 | 1.24 | 1.87 | 1.33 | 0.66 | 1.35 | 1.10 | 1.00 | 1.43 | 2.21 | 1.68 | 1.68 | 1.82 | 1.52 | 1.36 | 2.18 | 1.29 | 1.64 |
| lysoPC a C17:0 | 0.19 | 0.36 | 0.49 | 0.35 | 0.36 | 0.31 | 0.30 | 0.41 | 0.33 | 0.48 | 0.45 | 0.41 | 0.66 | 0.55 | 0.45 | 0.63 | 0.35 | 0.67 |
| lysoPC a C18:0 | 0.93 | 1.78 | 2.88 | 1.74 | 2.92 | 1.75 | 2.20 | 2.91 | 1.96 | 1.96 | 1.70 | 1.61 | 3.19 | 2.59 | 1.78 | 3.99 | 1.55 | 3.85 |
| lysoPC a C18:1 | 3.09 | 6.58 | 10.09 | 6.45 | 4.36 | 6.97 | 5.51 | 5.00 | 6.59 | 7.52 | 6.50 | 6.62 | 8.49 | 7.29 | 4.76 | 9.68 | 4.79 | 7.24 |
| lysoPC a C18:2 | 0.23 | 0.39 | 0.49 | 0.45 | 0.30 | 0.41 | 0.38 | 0.35 | 0.44 | 0.60 | 0.47 | 0.48 | 0.58 | 0.46 | 0.38 | 0.78 | 0.45 | 0.79 |
| lysoPC a C20:3 | 0.11 | 0.22 | 0.28 | 0.25 | 0.18 | 0.23 | 0.22 | 0.20 | 0.30 | 0.34 | 0.28 | 0.31 | 0.37 | 0.33 | 0.20 | 0.50 | 0.29 | 0.38 |
| lysoPC a C20:4 | 0.09 | 0.28 | 0.38 | 0.24 | 0.20 | 0.29 | 0.19 | 0.15 | 0.28 | 0.36 | 0.20 | 0.25 | 0.50 | 0.42 | 0.15 | 0.71 | 0.18 | 0.47 |
| lysoPC a C24:0 | 0.12 | 0.13 | 0.22 | 0.22 | 0.21 | 0.21 | 0.21 | 0.22 | 0.26 | 0.32 | 0.16 | 0.20 | 0.37 | 0.31 | 0.18 | 0.83 | 0.19 | 0.31 |
| lysoPC a C26:0 | 0.52 | 0.36 | 0.53 | 0.76 | 0.60 | 0.67 | 0.55 | 0.53 | 0.64 | 1.00 | 0.46 | 0.55 | 1.01 | 1.10 | 0.61 | 2.81 | 0.48 | 1.00 |
| lysoPC a C26:1 | 0.16 | 0.21 | 0.33 | 0.35 | 0.29 | 0.28 | 0.33 | 0.37 | 0.40 | 0.43 | 0.33 | 0.39 | 0.70 | 0.56 | 0.37 | 0.96 | 0.41 | 0.71 |
| lysoPC a C28:0 | 0.50 | 0.52 | 0.95 | 0.91 | 0.80 | 0.95 | 0.78 | 0.90 | 0.89 | 1.35 | 1.15 | 1.19 | 1.56 | 1.25 | 0.90 | 2.42 | 0.82 | 1.56 |
| lysoPC a C28:1 | 0.29 | 0.38 | 0.60 | 0.60 | 0.52 | 0.61 | 0.66 | 0.61 | 0.76 | 0.83 | 0.68 | 0.74 | 0.97 | 0.84 | 0.64 | 1.58 | 0.56 | 1.04 |
| PC aa C24:0 | 0.17 | 0.13 | 0.16 | 0.30 | 0.20 | 0.23 | 0.18 | 0.17 | 0.27 | 0.32 | 0.12 | 0.15 | 0.20 | 0.22 | 0.13 | 0.61 | 0.10 | 0.18 |
| PC aa C28:1 | 1.01 | 1.35 | 2.01 | 2.02 | 1.32 | 1.95 | 1.81 | 1.93 | 2.14 | 1.86 | 1.75 | 1.81 | 2.06 | 1.70 | 1.44 | 3.25 | 1.18 | 1.91 |
| PC aa C30:0 | 6.18 | 9.54 | 14.23 | 10.25 | 8.27 | 12.51 | 12.97 | 13.94 | 10.69 | 9.39 | 10.43 | 11.52 | 13.51 | 9.10 | 8.50 | 14.18 | 7.63 | 14.48 |
| PC aa C30:2 | 0.29 | 0.40 | 0.55 | 0.44 | 0.33 | 0.39 | 0.43 | 0.51 | 0.42 | 0.74 | 0.87 | 0.93 | 1.02 | 0.69 | 0.55 | 0.87 | 0.59 | 0.76 |
| PC aa C32:0 | 66.88 | 111.75 | 129.60 | 76.62 | 75.61 | 117.94 | 101.32 | 92.10 | 83.20 | 68.81 | 71.06 | 77.84 | 95.63 | 73.86 | 71.65 | 91.50 | 47.27 | 90.03 |
| PC aa C32:1 | 634.76 | 1218.41 | 1597.02 | 869.46 | 574.13 | 1162.36 | 841.16 | 904.91 | 673.43 | 779.58 | 914.56 | 974.06 | 1067.50 | 791.56 | 720.62 | 860.87 | 498.13 | 830.71 |
| PC aa C32:2 | 88.29 | 185.96 | 238.64 | 116.46 | 65.22 | 161.25 | 102.49 | 118.44 | 87.81 | 151.78 | 197.92 | 192.06 | 200.87 | 155.07 | 128.22 | 152.50 | 90.99 | 136.09 |
| PC aa C32:3 | 3.62 | 7.59 | 10.18 | 4.91 | 4.74 | 6.96 | 5.26 | 5.89 | 4.34 | 5.99 | 7.68 | 8.03 | 9.05 | 6.70 | 5.89 | 7.88 | 4.35 | 6.54 |
| PC aa C34:1 | 1182.90 | 2162.59 | 2811.60 | 1643.74 | 1369.45 | 1966.92 | 1859.37 | 1896.48 | 1468.71 | 1074.04 | 1203.25 | 1255.24 | 1577.32 | 1081.33 | 1070.67 | 1285.83 | 834.10 | 1458.91 |
| PC aa C34:2 | 454.38 | 950.86 | 1286.91 | 670.42 | 403.85 | 889.93 | 612.57 | 686.19 | 490.88 | 643.82 | 768.84 | 813.88 | 890.90 | 647.68 | 574.25 | 719.47 | 412.41 | 694.68 |
| PC aa C34:3 | 25.52 | 56.90 | 78.19 | 38.38 | 29.42 | 54.24 | 36.94 | 42.38 | 28.35 | 43.79 | 57.78 | 57.67 | 71.69 | 51.26 | 43.70 | 57.03 | 31.22 | 50.93 |
| PC aa C34:4 | 4.37 | 10.20 | 12.55 | 6.16 | 5.40 | 9.14 | 6.53 | 7.14 | 5.26 | 7.54 | 9.65 | 9.74 | 12.15 | 9.70 | 7.38 | 10.60 | 5.92 | 9.17 |
| PC aa C36:0 | 5.65 | 10.83 | 16.56 | 7.90 | 6.75 | 11.52 | 10.05 | 9.74 | 8.10 | 7.59 | 8.68 | 8.45 | 11.08 | 7.77 | 6.66 | 10.68 | 5.39 | 10.76 |
| PC aa C36:1 | 60.50 | 112.83 | 134.68 | 92.29 | 94.77 | 110.61 | 122.49 | 110.44 | 91.00 | 56.88 | 57.96 | 73.24 | 86.29 | 61.23 | 66.35 | 101.11 | 53.15 | 96.06 |
| PC aa C36:2 | 598.87 | 1077.50 | 1684.75 | 867.78 | 567.29 | 1204.75 | 958.69 | 959.65 | 727.40 | 574.99 | 683.49 | 729.15 | 807.12 | 583.73 | 548.23 | 711.14 | 414.63 | 724.90 |
| PC aa C36:3 | 77.14 | 164.84 | 233.97 | 120.15 | 95.66 | 167.13 | 131.10 | 143.28 | 94.83 | 102.36 | 124.23 | 128.94 | 169.46 | 123.04 | 108.78 | 149.65 | 80.15 | 143.67 |
| PC aa C36:4 | 30.21 | 61.05 | 85.00 | 45.91 | 46.10 | 64.79 | 55.58 | 61.82 | 42.20 | 42.42 | 52.17 | 53.83 | 78.01 | 55.13 | 49.92 | 76.13 | 37.76 | 66.72 |
| PC aa C36:5 | 12.97 | 26.92 | 34.99 | 20.50 | 19.53 | 30.04 | 22.72 | 25.93 | 18.03 | 19.67 | 24.48 | 24.36 | 35.11 | 28.60 | 22.90 | 39.87 | 16.94 | 32.05 |
| PC aa C36:6 | 5.87 | 13.98 | 19.68 | 9.27 | 6.84 | 12.73 | 9.87 | 11.19 | 6.57 | 8.01 | 9.65 | 9.89 | 12.25 | 9.11 | 7.55 | 11.49 | 6.03 | 10.37 |
| PC aa C38:0 | 2.95 | 6.09 | 8.79 | 4.42 | 3.76 | 6.39 | 6.08 | 6.34 | 4.42 | 5.89 | 6.76 | 7.15 | 7.59 | 5.81 | 5.37 | 8.14 | 4.25 | 7.70 |
| PC aa C38:1 | 0.04 | 0.04 | 0.04 | 0.04 | 0.07 | 0.04 | 0.04 | 0.04 | 0.04 | 0.04 | 0.04 | 0.03 | 0.14 | 0.54 | 0.49 | 0.92 | 0.18 | 0.75 |
| PC aa C38:3 | 9.89 | 17.17 | 25.96 | 14.84 | 12.48 | 20.30 | 18.79 | 17.99 | 13.43 | 12.92 | 13.53 | 14.57 | 19.10 | 14.54 | 13.44 | 19.41 | 10.84 | 20.80 |
| PC aa C38:4 | 13.35 | 26.54 | 38.41 | 22.00 | 21.54 | 31.09 | 29.84 | 32.81 | 22.86 | 21.66 | 24.97 | 25.73 | 36.78 | 26.05 | 22.71 | 38.50 | 19.58 | 36.08 |
| PC aa C38:5 | 23.16 | 41.49 | 61.56 | 34.93 | 31.97 | 51.94 | 43.88 | 47.16 | 32.53 | 30.77 | 35.90 | 37.03 | 52.51 | 43.45 | 32.94 | 65.59 | 28.56 | 53.44 |
| PC aa C38:6 | 17.40 | 34.88 | 50.31 | 29.44 | 31.10 | 42.05 | 39.80 | 43.48 | 30.81 | 25.17 | 28.96 | 31.77 | 45.96 | 34.68 | 29.53 | 53.42 | 24.70 | 46.30 |
| PC aa C40:2 | 1.64 | 2.63 | 3.40 | 2.10 | 1.46 | 2.76 | 2.80 | 2.53 | 1.97 | 1.77 | 1.50 | 2.33 | 2.13 | 1.82 | 1.61 | 2.16 | 1.55 | 2.27 |
| PC aa C40:3 | 1.13 | 1.97 | 2.38 | 1.35 | 1.12 | 2.06 | 1.90 | 1.78 | 1.55 | 1.38 | 1.21 | 1.58 | 1.70 | 1.41 | 1.24 | 1.83 | 1.22 | 2.03 |
| PC aa C40:4 | 1.62 | 2.78 | 3.66 | 2.84 | 2.29 | 2.92 | 3.80 | 3.44 | 2.43 | 2.01 | 2.20 | 2.50 | 3.17 | 2.60 | 2.14 | 3.38 | 2.00 | 3.49 |
| PC aa C40:5 | 5.38 | 9.22 | 14.23 | 8.47 | 8.56 | 11.35 | 12.06 | 13.23 | 9.07 | 5.90 | 6.51 | 7.59 | 10.10 | 7.59 | 7.47 | 13.09 | 6.46 | 13.29 |
| PC aa C40:6 | 7.20 | 14.01 | 21.30 | 12.25 | 11.08 | 17.65 | 16.14 | 17.64 | 12.41 | 11.17 | 12.47 | 12.44 | 18.46 | 14.96 | 11.47 | 22.31 | 10.80 | 20.48 |
| PC aa C42:0 | 0.27 | 0.45 | 0.62 | 0.45 | 0.26 | 0.54 | 0.39 | 0.37 | 0.31 | 0.38 | 0.49 | 0.41 | 0.58 | 0.53 | 0.43 | 0.64 | 0.34 | 0.60 |
| PC aa C42:1 | 0.46 | 0.60 | 0.75 | 0.53 | 0.36 | 0.57 | 0.59 | 0.37 | 0.61 | 0.80 | 0.70 | 0.80 | 0.90 | 0.72 | 0.66 | 0.90 | 0.46 | 0.87 |
| PC aa C42:2 | 0.56 | 0.83 | 1.15 | 0.64 | 0.56 | 0.86 | 1.10 | 0.83 | 0.71 | 1.06 | 0.82 | 1.08 | 1.15 | 1.01 | 0.83 | 1.33 | 0.82 | 1.13 |
| PC aa C42:4 | 0.38 | 0.78 | 0.79 | 0.45 | 0.47 | 0.64 | 0.72 | 0.68 | 0.50 | 0.45 | 0.47 | 0.57 | 0.64 | 0.53 | 0.49 | 0.67 | 0.41 | 0.72 |
| PC aa C42:5 | 0.83 | 1.26 | 2.04 | 1.35 | 1.38 | 1.68 | 2.24 | 1.84 | 1.62 | 0.99 | 0.92 | 1.11 | 1.55 | 1.07 | 1.10 | 1.75 | 1.21 | 2.03 |
| PC aa C42:6 | 2.19 | 4.31 | 6.88 | 4.27 | 3.13 | 5.34 | 5.88 | 6.50 | 4.17 | 2.70 | 2.63 | 3.16 | 4.80 | 3.26 | 3.08 | 5.35 | 2.85 | 5.04 |
| PC ae C30:0 | 1.36 | 2.10 | 3.11 | 2.13 | 1.47 | 2.63 | 2.27 | 2.43 | 1.90 | 2.87 | 3.28 | 3.36 | 3.48 | 2.36 | 2.30 | 3.37 | 1.86 | 3.20 |
| PC ae C30:1 | 0.59 | 0.88 | 1.25 | 0.87 | 0.79 | 1.10 | 1.16 | 1.32 | 0.96 | 1.23 | 1.46 | 1.62 | 1.54 | 1.15 | 0.98 | 1.59 | 0.86 | 1.55 |
| PC ae C30:2 | 0.09 | 0.15 | 0.19 | 0.14 | 0.13 | 0.17 | 0.16 | 0.18 | 0.15 | 0.18 | 0.20 | 0.21 | 0.24 | 0.19 | 0.17 | 0.27 | 0.16 | 0.24 |
| PC ae C32:1 | 98.01 | 188.44 | 249.53 | 129.17 | 74.43 | 168.54 | 106.06 | 118.41 | 88.57 | 163.59 | 192.11 | 203.75 | 186.98 | 142.40 | 137.59 | 138.88 | 81.51 | 132.55 |
| PC ae C32:2 | 20.56 | 40.41 | 50.74 | 27.37 | 21.80 | 36.66 | 26.69 | 29.50 | 21.31 | 25.55 | 31.85 | 34.22 | 33.74 | 25.05 | 24.27 | 28.28 | 16.98 | 26.87 |
| PC ae C34:0 | 14.82 | 27.21 | 29.32 | 17.95 | 13.71 | 24.00 | 20.63 | 15.25 | 16.13 | 19.94 | 19.86 | 22.59 | 26.04 | 21.29 | 20.43 | 23.49 | 13.44 | 22.08 |
| PC ae C34:1 | 211.74 | 389.27 | 533.38 | 271.14 | 196.39 | 386.11 | 265.43 | 280.97 | 211.51 | 253.57 | 291.30 | 306.12 | 329.80 | 245.28 | 246.62 | 275.57 | 154.20 | 271.44 |
| PC ae C34:2 | 80.55 | 149.08 | 199.30 | 106.60 | 80.77 | 155.73 | 106.21 | 114.98 | 83.82 | 98.61 | 117.69 | 126.21 | 136.56 | 100.27 | 95.57 | 103.95 | 63.88 | 106.75 |
| PC ae C34:3 | 9.84 | 21.06 | 29.12 | 14.31 | 11.80 | 20.03 | 15.25 | 16.79 | 10.91 | 12.47 | 14.88 | 15.99 | 18.53 | 13.60 | 11.60 | 14.69 | 8.22 | 13.85 |
| PC ae C36:0 | 6.30 | 14.34 | 17.87 | 8.67 | 6.27 | 11.47 | 9.39 | 9.61 | 7.51 | 7.05 | 8.75 | 8.15 | 10.32 | 7.91 | 6.46 | 9.80 | 4.34 | 7.58 |
| PC ae C36:1 | 43.42 | 72.06 | 94.59 | 55.88 | 47.06 | 74.73 | 65.16 | 64.49 | 50.77 | 47.94 | 53.23 | 56.98 | 68.88 | 50.66 | 50.05 | 61.64 | 36.41 | 61.57 |
| PC ae C36:2 | 78.79 | 143.75 | 212.80 | 111.02 | 82.78 | 155.26 | 115.01 | 122.38 | 87.34 | 92.28 | 107.70 | 118.49 | 136.89 | 97.30 | 93.94 | 110.95 | 64.30 | 112.72 |
| PC ae C36:3 | 31.43 | 67.68 | 103.73 | 46.04 | 31.13 | 62.32 | 46.58 | 54.94 | 32.47 | 36.91 | 46.57 | 47.54 | 56.68 | 38.19 | 34.43 | 45.13 | 24.78 | 44.88 |
| PC ae C36:4 | 17.60 | 34.13 | 50.93 | 24.50 | 16.92 | 32.52 | 25.53 | 28.01 | 20.47 | 28.39 | 33.87 | 36.08 | 40.89 | 29.39 | 28.69 | 36.02 | 21.00 | 35.61 |
| PC ae C36:5 | 10.04 | 20.04 | 28.21 | 14.65 | 11.72 | 20.21 | 16.75 | 16.97 | 11.93 | 17.86 | 22.55 | 22.26 | 26.64 | 19.37 | 17.87 | 25.16 | 13.58 | 23.56 |
| PC ae C38:0 | 3.95 | 8.13 | 11.44 | 5.56 | 4.62 | 8.04 | 7.36 | 7.50 | 5.63 | 6.52 | 8.37 | 8.33 | 10.65 | 7.92 | 6.38 | 9.70 | 4.88 | 8.44 |
| PC ae C38:1 | 2.62 | 4.49 | 6.36 | 3.77 | 2.64 | 5.17 | 4.66 | 3.75 | 3.42 | 4.24 | 3.39 | 4.15 | 4.64 | 3.78 | 3.25 | 5.40 | 2.64 | 4.26 |
| PC ae C38:2 | 14.45 | 24.02 | 31.90 | 19.85 | 14.82 | 25.11 | 22.60 | 21.29 | 16.27 | 16.53 | 17.69 | 19.38 | 21.81 | 16.15 | 15.77 | 20.26 | 12.71 | 20.78 |
| PC ae C38:3 | 8.68 | 17.29 | 24.62 | 11.85 | 9.59 | 17.13 | 14.74 | 15.47 | 10.08 | 9.39 | 10.14 | 11.63 | 14.42 | 10.11 | 9.21 | 13.13 | 7.60 | 13.21 |
| PC ae C38:4 | 7.84 | 15.74 | 24.91 | 12.04 | 10.19 | 15.96 | 14.13 | 14.92 | 10.89 | 13.04 | 14.98 | 16.24 | 20.32 | 14.28 | 12.58 | 18.99 | 9.93 | 18.91 |
| PC ae C38:5 | 13.11 | 25.34 | 36.44 | 17.69 | 13.58 | 24.91 | 19.88 | 22.02 | 16.59 | 22.55 | 27.12 | 29.16 | 32.51 | 23.06 | 20.77 | 32.46 | 16.34 | 30.48 |
| PC ae C38:6 | 10.58 | 22.78 | 33.02 | 16.28 | 11.50 | 22.10 | 18.66 | 19.63 | 13.58 | 20.46 | 24.65 | 26.24 | 28.54 | 20.78 | 20.11 | 29.08 | 15.50 | 27.26 |
| PC ae C40:1 | 0.66 | 1.48 | 1.88 | 0.98 | 1.19 | 1.72 | 1.67 | 1.72 | 1.28 | 1.24 | 1.59 | 1.47 | 2.02 | 1.91 | 1.39 | 2.38 | 1.13 | 2.16 |
| PC ae C40:2 | 3.49 | 5.86 | 6.17 | 3.95 | 3.47 | 4.68 | 4.16 | 4.18 | 3.22 | 2.82 | 3.07 | 3.20 | 3.61 | 2.80 | 2.42 | 3.48 | 2.39 | 3.34 |
| PC ae C40:3 | 1.58 | 2.72 | 3.08 | 1.89 | 1.82 | 2.38 | 2.38 | 2.28 | 1.91 | 1.89 | 1.93 | 1.82 | 2.75 | 2.07 | 1.73 | 2.69 | 1.34 | 2.26 |
| PC ae C40:4 | 1.65 | 2.86 | 3.83 | 2.59 | 1.74 | 2.96 | 2.98 | 2.96 | 2.35 | 2.75 | 2.84 | 3.36 | 3.56 | 2.58 | 2.69 | 3.85 | 2.24 | 3.67 |
| PC ae C40:5 | 5.17 | 8.51 | 13.97 | 6.83 | 4.90 | 9.10 | 8.63 | 8.61 | 6.09 | 8.01 | 8.40 | 9.48 | 11.17 | 8.36 | 8.18 | 11.26 | 6.56 | 11.12 |
| PC ae C40:6 | 3.76 | 7.48 | 10.84 | 5.71 | 4.75 | 7.93 | 7.18 | 7.37 | 5.25 | 7.00 | 7.82 | 8.29 | 9.59 | 7.33 | 6.57 | 9.91 | 5.16 | 9.79 |
| PC ae C42:0 | 1.08 | 1.64 | 2.54 | 1.55 | 1.71 | 2.16 | 2.31 | 2.26 | 1.47 | 1.31 | 1.48 | 1.52 | 1.96 | 1.81 | 1.44 | 2.15 | 1.36 | 2.17 |
| PC ae C42:2 | 0.40 | 0.65 | 0.84 | 0.66 | 0.65 | 0.86 | 0.75 | 0.87 | 0.61 | 0.74 | 0.56 | 0.75 | 0.99 | 0.71 | 0.63 | 1.11 | 0.53 | 1.12 |
| PC ae C42:3 | 0.67 | 0.91 | 1.43 | 0.65 | 0.78 | 1.11 | 1.18 | 1.11 | 0.88 | 0.73 | 0.86 | 0.81 | 1.15 | 1.07 | 0.74 | 1.55 | 0.65 | 1.11 |
| PC ae C42:4 | 0.50 | 0.99 | 1.02 | 0.72 | 0.52 | 0.83 | 0.88 | 0.78 | 0.73 | 0.61 | 0.65 | 0.72 | 1.03 | 0.71 | 0.58 | 1.08 | 0.63 | 1.01 |
| PC ae C42:5 | 1.28 | 2.15 | 2.46 | 1.70 | 1.45 | 2.03 | 2.14 | 1.93 | 1.63 | 1.54 | 1.44 | 1.92 | 2.16 | 1.73 | 1.63 | 2.22 | 1.37 | 2.35 |
| PC ae C44:3 | 0.38 | 0.39 | 0.64 | 0.41 | 0.39 | 0.55 | 0.46 | 0.41 | 0.37 | 0.34 | 0.38 | 0.43 | 0.50 | 0.58 | 0.37 | 1.07 | 0.22 | 0.43 |
| PC ae C44:4 | 0.32 | 0.50 | 0.50 | 0.29 | 0.36 | 0.38 | 0.40 | 0.34 | 0.23 | 0.24 | 0.26 | 0.27 | 0.32 | 0.43 | 0.28 | 0.45 | 0.26 | 0.34 |
| PC ae C44:5 | 0.26 | 0.50 | 0.57 | 0.40 | 0.41 | 0.47 | 0.48 | 0.40 | 0.47 | 0.44 | 0.35 | 0.41 | 0.46 | 0.42 | 0.35 | 0.47 | 0.26 | 0.51 |
| PC ae C44:6 | 0.36 | 0.61 | 0.88 | 0.44 | 0.39 | 0.57 | 0.51 | 0.52 | 0.40 | 0.45 | 0.47 | 0.48 | 0.55 | 0.51 | 0.40 | 0.64 | 0.36 | 0.53 |
| Sugars | 1805.38 | 2700.42 | 4305.21 | 3142.69 | 2348.16 | 2303.33 | 3670.15 | 3438.34 | 3956.49 | 3203.01 | 4279.05 | 3689.19 | 3389.13 | 4311.86 | 3248.64 | 2790.08 | 3073.88 | 4987.83 |
| Ala | 810.00 | 1423.33 | 1520.00 | 930.00 | 770.00 | 880.00 | 693.33 | 680.00 | 746.67 | 4166.67 | 4766.67 | 4466.67 | 2963.33 | 2920.00 | 3163.33 | 1553.33 | 1876.67 | 2380.00 |
| Arg | 179.67 | 228.67 | 269.33 | 181.00 | 145.33 | 110.00 | 142.67 | 150.67 | 169.67 | 300.00 | 293.67 | 298.00 | 235.67 | 215.33 | 217.00 | 137.00 | 170.00 | 220.00 |
| Asn | 1350.00 | 1903.33 | 1856.67 | 1643.33 | 1240.00 | 1533.33 | 1260.00 | 1126.67 | 1240.00 | 1993.33 | 2136.67 | 2083.33 | 2380.00 | 1420.00 | 1866.67 | 866.67 | 1286.67 | 1393.33 |
| Asp | 128.00 | 216.33 | 228.00 | 226.33 | 186.67 | 149.33 | 187.00 | 214.67 | 275.00 | 71.33 | 63.33 | 51.67 | 211.67 | 169.00 | 218.33 | 201.00 | 299.33 | 305.33 |
| Cit | 10.07 | 19.23 | 20.20 | 10.53 | 6.23 | 8.27 | 7.77 | 6.27 | 6.90 | 22.27 | 21.23 | 22.20 | 15.43 | 15.10 | 16.57 | 6.93 | 9.20 | 11.43 |
| Gln | 1100.00 | 1680.00 | 1973.33 | 2106.67 | 2176.67 | 1683.33 | 1760.00 | 1536.67 | 1786.67 | 21.57 | 19.20 | 15.13 | 603.33 | 423.33 | 420.00 | 706.67 | 540.00 | 733.33 |
| Glu | 2653.33 | 4800.00 | 4366.67 | 4400.00 | 3223.33 | 3800.00 | 4266.67 | 3933.33 | 4600.00 | 730.00 | 970.00 | 843.33 | 2763.33 | 2563.33 | 2570.00 | 2596.67 | 2763.33 | 3016.67 |
| Gly | 550.00 | 913.33 | 1010.00 | 756.67 | 726.67 | 573.33 | 676.67 | 596.67 | 653.33 | 970.00 | 860.00 | 990.00 | 703.33 | 560.00 | 716.67 | 516.67 | 483.33 | 643.33 |
| His | 70.67 | 114.33 | 131.00 | 81.00 | 53.00 | 65.33 | 53.00 | 53.67 | 57.00 | 142.33 | 132.67 | 155.33 | 108.00 | 105.00 | 104.33 | 55.67 | 56.00 | 75.00 |
| Ile | 206.67 | 301.33 | 373.33 | 225.00 | 142.33 | 193.00 | 169.67 | 159.00 | 167.00 | 463.33 | 463.33 | 503.33 | 343.33 | 327.33 | 353.33 | 204.00 | 209.00 | 267.00 |
| Leu | 242.67 | 304.33 | 366.67 | 207.00 | 140.67 | 179.33 | 193.33 | 149.00 | 182.33 | 446.67 | 393.33 | 453.33 | 340.00 | 283.33 | 343.33 | 225.33 | 229.00 | 264.00 |
| Lys | 9.83 | 21.10 | 21.83 | 13.00 | 14.63 | 5.13 | 11.50 | 12.00 | 16.67 | 26.53 | 19.50 | 28.00 | 18.60 | 15.60 | 14.87 | 12.73 | 13.53 | 27.83 |
| Met | 40.33 | 60.00 | 78.33 | 51.00 | 48.67 | 48.67 | 44.33 | 42.33 | 44.67 | 75.67 | 75.00 | 82.00 | 67.33 | 65.67 | 69.33 | 47.00 | 45.67 | 57.00 |
| Orn | 13.70 | 31.27 | 32.13 | 21.40 | 12.77 | 7.70 | 11.50 | 12.83 | 19.00 | 46.67 | 31.80 | 43.00 | 28.00 | 18.10 | 21.17 | 10.63 | 13.93 | 23.03 |
| Phe | 38.33 | 54.00 | 70.00 | 47.67 | 40.33 | 41.33 | 42.33 | 38.67 | 42.00 | 69.33 | 71.00 | 75.33 | 61.00 | 58.00 | 64.00 | 45.33 | 45.33 | 55.00 |
| Pro | 523.33 | 580.00 | 616.67 | 540.00 | 319.33 | 413.33 | 356.67 | 350.00 | 346.67 | 570.00 | 503.33 | 566.67 | 530.00 | 500.00 | 530.00 | 413.33 | 416.67 | 433.33 |
| Ser | 10.50 | 17.73 | 13.97 | 27.73 | 126.00 | 22.90 | 78.00 | 60.67 | 77.33 | 62.33 | 53.33 | 72.67 | 49.00 | 25.53 | 31.83 | 85.33 | 61.33 | 107.67 |
| Thr | 195.33 | 312.33 | 325.00 | 236.33 | 220.33 | 179.33 | 180.00 | 187.00 | 198.00 | 350.00 | 436.67 | 390.00 | 321.67 | 260.67 | 325.67 | 179.67 | 222.67 | 274.33 |
| Trp | 13.37 | 18.60 | 24.23 | 16.27 | 13.10 | 13.90 | 13.73 | 12.53 | 13.93 | 26.87 | 27.17 | 28.67 | 22.40 | 19.07 | 21.50 | 15.43 | 14.73 | 17.77 |
| Tyr | 77.33 | 112.67 | 142.67 | 86.67 | 60.67 | 71.00 | 65.67 | 60.00 | 67.67 | 154.33 | 156.33 | 169.00 | 114.67 | 107.33 | 119.00 | 72.67 | 70.67 | 94.33 |
| Val | 43.00 | 60.00 | 79.33 | 50.67 | 45.00 | 44.33 | 48.00 | 44.33 | 47.33 | 83.00 | 81.00 | 89.67 | 69.67 | 67.67 | 69.33 | 55.33 | 52.33 | 69.00 |
| Ac-Orn | 3.47 | 7.83 | 9.13 | 5.23 | 2.54 | 3.40 | 2.23 | 2.32 | 2.79 | 16.30 | 8.50 | 12.37 | 7.97 | 6.37 | 7.13 | 2.77 | 2.69 | 4.50 |
| ADMA | 0.35 | 0.54 | 0.65 | 0.60 | 0.20 | 0.49 | 0.31 | 0.57 | 0.58 | 0.85 | 0.97 | 0.54 | 0.63 | 0.75 | 0.47 | 0.42 | 0.41 | 0.72 |
| alpha-AAA | 2.44 | 6.87 | 8.30 | 5.00 | 2.58 | 3.83 | 2.31 | 2.15 | 2.84 | 3.47 | 2.74 | 2.40 | 5.13 | 5.27 | 3.80 | 2.85 | 3.01 | 3.63 |
| Carnosine | 0.35 | 0.52 | 0.81 | 0.53 | 0.43 | 0.38 | 0.55 | 0.50 | 0.58 | 0.34 | 0.52 | 0.44 | 0.46 | 0.64 | 0.41 | 0.36 | 0.44 | 0.87 |
| DOPA | 0.13 | 0.14 | 0.23 | 0.16 | 0.13 | 0.11 | 0.11 | 0.15 | 0.11 | 0.27 | 0.34 | 0.42 | 0.30 | 0.25 | 0.33 | 0.15 | 0.23 | 0.23 |
| Kynurenine | 0.86 | 1.09 | 1.56 | 0.96 | 0.50 | 0.77 | 0.53 | 0.62 | 0.67 | 1.81 | 1.73 | 1.97 | 1.21 | 1.13 | 1.29 | 0.60 | 0.69 | 0.90 |
| Met-SO | 3.32 | 4.43 | 6.30 | 4.03 | 2.28 | 3.57 | 2.48 | 2.65 | 3.00 | 5.77 | 5.10 | 5.97 | 4.00 | 3.70 | 3.90 | 2.68 | 2.81 | 2.91 |
| Putrescine | 28.77 | 50.33 | 57.00 | 37.00 | 57.00 | 16.53 | 38.00 | 36.33 | 36.33 | 37.67 | 39.67 | 35.00 | 43.00 | 36.67 | 22.37 | 25.93 | 33.33 | 39.67 |
| Serotonin | 0.09 | 0.12 | 0.16 | 0.13 | 0.11 | 0.11 | 0.16 | 0.15 | 0.15 | 0.09 | 0.11 | 0.09 | 0.10 | 0.13 | 0.06 | 0.09 | 0.10 | 0.18 |
| Spermidine | 10.40 | 11.67 | 10.40 | 9.77 | 7.73 | 3.63 | 8.70 | 6.00 | 7.30 | 10.40 | 11.27 | 9.33 | 8.33 | 6.37 | 9.77 | 4.67 | 5.97 | 9.77 |
| Spermine | 9.70 | 8.87 | 6.50 | 7.87 | 4.50 | 2.17 | 7.63 | 4.60 | 5.93 | 6.63 | 8.33 | 5.63 | 3.40 | 1.97 | 7.23 | 2.29 | 3.60 | 5.30 |
| t4-OH-Pro | 576.67 | 713.33 | 900.00 | 643.33 | 360.00 | 536.67 | 433.33 | 430.00 | 426.67 | 1093.33 | 1080.00 | 1180.00 | 903.33 | 770.00 | 900.00 | 466.67 | 493.33 | 553.33 |
| Taurine | 188.33 | 221.33 | 217.00 | 201.33 | 123.33 | 182.00 | 226.67 | 210.00 | 211.00 | 259.33 | 254.33 | 234.33 | 242.33 | 238.00 | 233.33 | 217.00 | 248.33 | 253.33 |
| total DMA | 0.38 | 0.72 | 0.81 | 0.40 | 0.33 | 0.34 | 0.30 | 0.30 | 0.36 | 0.71 | 1.01 | 0.88 | 0.65 | 0.58 | 0.50 | 0.30 | 0.32 | 0.59 |
| SM (OH) C14:1 | 0.89 | 1.37 | 2.17 | 1.56 | 1.63 | 1.70 | 1.87 | 1.95 | 1.45 | 1.57 | 1.76 | 1.90 | 3.16 | 2.01 | 1.67 | 2.74 | 1.55 | 2.94 |
| SM (OH) C16:1 | 0.13 | 0.22 | 0.32 | 0.26 | 0.30 | 0.29 | 0.44 | 0.36 | 0.30 | 0.28 | 0.27 | 0.23 | 0.60 | 0.35 | 0.28 | 0.54 | 0.33 | 0.60 |
| SM (OH) C22:1 | 0.03 | 0.07 | 0.18 | 0.07 | 0.08 | 0.05 | 0.06 | 0.13 | 0.08 | 0.16 | 0.13 | 0.13 | 0.19 | 0.11 | 0.12 | 0.14 | 0.08 | 0.14 |
| SM (OH) C22:2 | 0.02 | 0.02 | 0.00 | 0.09 | 0.06 | 0.01 | 0.01 | 0.00 | 0.05 | 0.14 | 0.14 | 0.17 | 0.13 | 0.16 | 0.15 | 0.24 | 0.04 | 0.22 |
| SM (OH) C24:1 | 0.00 | 0.00 | 0.00 | 0.00 | 0.00 | 0.00 | 0.00 | 0.00 | 0.00 | 0.00 | 0.00 | 0.00 | 0.01 | 0.00 | 0.01 | 0.00 | 0.00 | 0.00 |
| SM C16:0 | 11.07 | 17.89 | 28.04 | 20.34 | 20.74 | 24.28 | 27.88 | 27.92 | 22.12 | 15.35 | 17.32 | 19.54 | 31.88 | 19.46 | 16.69 | 30.95 | 15.64 | 32.88 |
| SM C16:1 | 1.69 | 3.00 | 5.16 | 3.17 | 2.90 | 3.62 | 4.33 | 4.62 | 3.46 | 3.25 | 3.60 | 3.80 | 6.13 | 4.38 | 3.25 | 5.63 | 3.18 | 6.12 |
| SM C18:0 | 0.06 | 0.03 | 0.08 | 0.06 | 0.28 | 0.13 | 0.46 | 0.22 | 0.24 | 0.05 | 0.05 | 0.05 | 0.05 | 0.05 | 0.05 | 0.09 | 0.05 | 0.16 |
| SM C18:1 | 0.33 | 0.58 | 0.88 | 0.61 | 0.61 | 0.66 | 0.71 | 0.82 | 0.51 | 0.64 | 0.72 | 0.76 | 1.29 | 0.89 | 0.64 | 1.01 | 0.62 | 1.33 |
| SM C20:2 | 0.07 | 0.16 | 0.31 | 0.17 | 0.12 | 0.17 | 0.13 | 0.22 | 0.13 | 0.16 | 0.25 | 0.23 | 0.32 | 0.15 | 0.10 | 0.25 | 0.07 | 0.25 |
| SM C22:3 | 0.04 | 0.02 | 0.14 | 0.05 | 0.10 | 0.03 | 0.20 | 0.16 | 0.06 | 0.14 | 0.08 | 0.11 | 0.14 | 0.06 | 0.00 | 0.12 | 0.11 | 0.20 |
| SM C24:0 | 0.88 | 1.28 | 2.31 | 1.60 | 1.25 | 1.65 | 2.53 | 2.32 | 1.74 | 1.65 | 1.72 | 2.06 | 2.76 | 1.63 | 1.43 | 2.41 | 1.79 | 3.35 |
| SM C24:1 | 0.36 | 0.43 | 0.65 | 0.57 | 0.38 | 0.53 | 0.84 | 0.74 | 0.62 | 0.79 | 0.77 | 0.96 | 1.10 | 0.84 | 0.73 | 0.93 | 0.81 | 1.30 |
| SM C26:0 | 0.01 | 0.02 | 0.02 | 0.01 | 0.03 | 0.01 | 0.04 | 0.03 | 0.02 | 0.03 | 0.05 | 0.03 | 0.02 | 0.01 | 0.03 | 0.04 | 0.03 | 0.04 |
| SM C26:1 | 0.03 | 0.04 | 0.09 | 0.07 | 0.06 | 0.05 | 0.07 | 0.07 | 0.07 | 0.07 | 0.09 | 0.06 | 0.13 | 0.06 | 0.05 | 0.08 | 0.04 | 0.13 |
| C0 | 6.22 | 7.44 | 10.10 | 7.52 | 6.65 | 7.67 | 9.17 | 7.49 | 9.64 | 22.50 | 23.29 | 21.81 | 27.21 | 26.81 | 26.19 | 23.85 | 24.00 | 25.31 |
| C14 | 0.04 | 0.06 | 0.07 | 0.05 | 0.06 | 0.04 | 0.08 | 0.07 | 0.06 | 0.07 | 0.07 | 0.07 | 0.07 | 0.06 | 0.06 | 0.08 | 0.08 | 0.08 |
| C16 | 0.02 | 0.04 | 0.05 | 0.03 | 0.03 | 0.03 | 0.04 | 0.04 | 0.04 | 0.10 | 0.10 | 0.10 | 0.13 | 0.10 | 0.12 | 0.24 | 0.15 | 0.20 |
| C18 | 0.02 | 0.03 | 0.04 | 0.03 | 0.03 | 0.03 | 0.03 | 0.03 | 0.03 | 0.03 | 0.04 | 0.03 | 0.05 | 0.05 | 0.04 | 0.13 | 0.07 | 0.10 |
| C18:1 | 0.04 | 0.05 | 0.05 | 0.05 | 0.10 | 0.05 | 0.11 | 0.09 | 0.07 | 0.08 | 0.10 | 0.09 | 0.10 | 0.07 | 0.09 | 0.15 | 0.17 | 0.15 |
| C2 | 4.17 | 5.52 | 6.59 | 5.46 | 2.88 | 5.38 | 4.43 | 4.25 | 3.73 | 15.98 | 17.66 | 18.38 | 11.41 | 10.18 | 10.31 | 6.84 | 6.67 | 8.43 |
| C3 | 0.46 | 0.69 | 0.90 | 0.73 | 0.46 | 0.73 | 0.64 | 0.59 | 0.54 | 1.83 | 2.00 | 1.73 | 2.18 | 2.00 | 1.80 | 1.48 | 1.63 | 1.73 |
| C3-DC-M / C5-OH | 0.03 | 0.05 | 0.05 | 0.04 | 0.04 | 0.04 | 0.05 | 0.05 | 0.05 | 0.07 | 0.07 | 0.07 | 0.08 | 0.07 | 0.08 | 0.07 | 0.06 | 0.07 |
| C4 | 3.43 | 4.94 | 6.54 | 4.72 | 2.15 | 4.65 | 3.94 | 3.68 | 3.83 | 12.39 | 12.90 | 12.43 | 11.86 | 11.78 | 11.99 | 8.71 | 9.25 | 10.22 |
| C5 | 9.58 | 12.53 | 16.48 | 13.13 | 7.23 | 13.43 | 15.28 | 13.22 | 13.69 | 37.32 | 41.86 | 39.99 | 27.26 | 26.47 | 28.28 | 25.18 | 24.32 | 28.17 |
| C5-M-DC | 0.06 | 0.06 | 0.06 | 0.05 | 0.10 | 0.05 | 0.08 | 0.08 | 0.06 | 0.06 | 0.08 | 0.07 | 0.07 | 0.05 | 0.06 | 0.07 | 0.09 | 0.10 |
| C5:1 | 0.04 | 0.05 | 0.06 | 0.05 | 0.05 | 0.05 | 0.05 | 0.05 | 0.06 | 0.09 | 0.10 | 0.08 | 0.09 | 0.09 | 0.09 | 0.09 | 0.08 | 0.10 |
| C6 / C4:1-DC | 0.03 | 0.03 | 0.04 | 0.03 | 0.03 | 0.03 | 0.03 | 0.03 | 0.03 | 0.07 | 0.08 | 0.08 | 0.07 | 0.06 | 0.06 | 0.04 | 0.05 | 0.05 |

1. first replicate, b) second replicate, c) third replicate

**Supplementary Table S2**. Concentration ratio (treated versus untreated) of the discriminant metabolites (from OPLS-DA, s-plot) in KRAS-WT and KRAS-G12C clones treated with BEZ235(25 nM) or BKM120 (1 µM), at 6, 24 and 48h.

|  | **BEZ235** | | | | | | **BKM120** | | | | | |
| --- | --- | --- | --- | --- | --- | --- | --- | --- | --- | --- | --- | --- |
|  | **WT KRAS** | | | **G12C KRAS** | | | **WT KRAS** | | | **G12C KRAS** | | |
| **Metabolite** | **6** | **24** | **48** | **6** | **24** | **48** | **6** | **24** | **48** | **6** | **24** | **48** |
| Sugars | 1.29 | 0.92 | 0.88 | 1.58 | 0.67 | 1.00 | 1.00 | 0.58 | 1.26 | 1.33 | 0.37 | 1.00 |
| Ala | 1.37 | 1.41 | 0.69 | 2.15 | 1.16 | 0.68 | 1.00 | 0.85 | 0.56 | 2.31 | 0.65 | 0.43 |
| Arg | 1.43 | 1.00 | 0.64 | 1.51 | 1.00 | 0.75 | 1.00 | 0.64 | 0.68 | 1.48 | 1.00 | 0.59 |
| Asn | 1.38 | 1.30 | 0.86 | 1.92 | 1.16 | 0.91 | 1.00 | 0.74 | 0.71 | 1.77 | 0.72 | 0.57 |
| Asp | 1.61 | 1.33 | 1.00 | 1.66 | 1.00 | 3.21 | 1.00 | 0.89 | 1.18 | 1.89 | 0.76 | 4.32 |
| Cit | 1.00 | 1.00 | 0.51 | 1.00 | 1.00 | 1.00 | 1.00 | 1.00 | 0.42 | 1.00 | 1.00 | 0.42 |
| Gln | 1.70 | 1.53 | 1.26 | 2.35 | 2.06 | 25.88 | 1.00 | 0.94 | 1.07 | 1.79 | 1.67 | 35.42 |
| Glu | 1.18 | 0.95 | 0.97 | 1.63 | 1.10 | 3.10 | 1.00 | 0.65 | 1.08 | 1.63 | 0.73 | 3.29 |
| Gly | 1.29 | 1.30 | 0.83 | 2.59 | 1.42 | 0.70 | 1.00 | 0.89 | 0.78 | 2.40 | 1.00 | 0.58 |
| His | 1.00 | 1.14 | 0.63 | 1.87 | 1.15 | 0.74 | 1.00 | 1.00 | 0.52 | 1.96 | 1.00 | 0.43 |
| Ile | 1.38 | 1.07 | 0.64 | 1.81 | 1.07 | 0.72 | 1.00 | 0.70 | 0.56 | 1.91 | 0.76 | 0.48 |
| Leu | 1.65 | 1.10 | 0.58 | 1.79 | 1.08 | 0.75 | 1.00 | 0.78 | 0.57 | 1.83 | 1.00 | 0.56 |
| Lys | 1.00 | 1.00 | 0.62 | 1.00 | 1.00 | 1.00 | 1.00 | 1.00 | 1.00 | 1.00 | 1.00 | 1.00 |
| Met | 1.00 | 1.22 | 0.83 | 2.06 | 1.25 | 1.00 | 1.00 | 1.00 | 0.74 | 1.89 | 1.00 | 0.64 |
| Orn | 1.00 | 1.00 | 0.54 | 1.00 | 1.00 | 1.00 | 1.00 | 1.00 | 0.56 | 1.00 | 1.00 | 0.39 |
| Phe | 1.00 | 1.18 | 0.80 | 2.03 | 1.22 | 1.00 | 1.00 | 1.00 | 0.76 | 2.00 | 1.00 | 0.68 |
| Pro | 1.10 | 1.00 | 0.74 | 1.69 | 1.03 | 0.95 | 1.00 | 0.68 | 0.61 | 1.79 | 0.76 | 0.77 |
| Ser | 2.45 | 1.59 | 4.19 | 2.55 | 1.55 | 0.56 | 1.00 | 1.28 | 5.12 | 1.77 | 1.35 | 1.00 |
| Thr | 1.66 | 1.31 | 0.76 | 2.41 | 1.43 | 0.77 | 1.00 | 0.85 | 0.68 | 2.13 | 1.00 | 0.58 |
| Trp | 1.00 | 1.00 | 1.00 | 1.00 | 1.00 | 1.00 | 1.00 | 1.00 | 1.00 | 1.00 | 1.00 | 0.58 |
| Tyr | 1.00 | 1.13 | 0.66 | 1.88 | 1.16 | 0.71 | 1.00 | 0.77 | 0.58 | 1.97 | 1.00 | 0.50 |
| Val | 1.00 | 1.20 | 0.77 | 2.06 | 1.28 | 1.00 | 1.00 | 1.00 | 0.77 | 1.94 | 1.00 | 0.70 |
| Putrescine | 1.00 | 1.41 | 1.00 | 1.00 | 1.34 | 1.00 | 1.00 | 1.00 | 1.00 | 1.00 | 1.00 | 1.00 |
| Spermine | 1.00 | 1.00 | 1.00 | 1.00 | 1.00 | 1.00 | 1.00 | 1.00 | 1.00 | 1.00 | 1.00 | 1.00 |
| t4-OH-Pro | 1.31 | 1.23 | 0.70 | 1.77 | 1.16 | 0.77 | 1.00 | 0.72 | 0.59 | 1.71 | 0.69 | 0.45 |
| Taurine | 0.89 | 0.91 | 0.81 | 1.28 | 0.96 | 0.95 | 1.00 | 0.79 | 1.00 | 1.30 | 0.81 | 1.00 |
| C5 | 1.00 | 1.00 | 1.00 | 1.00 | 1.00 | 1.00 | 1.00 | 1.00 | 1.00 | 1.41 | 0.59 | 0.65 |
| SM C16:0 | 1.00 | 1.75 | 1.00 | 1.00 | 1.00 | 1.00 | 1.00 | 1.00 | 1.00 | 1.00 | 1.00 | 1.00 |
| PC aa C30:0 | 1.00 | 1.59 | 1.00 | 1.00 | 1.00 | 1.00 | 1.00 | 1.00 | 1.00 | 1.00 | 1.00 | 1.00 |
| PC aa C32:0 | 1.45 | 1.62 | 1.00 | 1.00 | 1.20 | 1.00 | 1.00 | 1.00 | 1.00 | 1.00 | 1.00 | 1.00 |
| PC aa C32:1 | 1.33 | 1.21 | 0.76 | 1.00 | 0.77 | 1.00 | 1.00 | 0.75 | 0.70 | 0.75 | 0.50 | 0.82 |
| PC aa C32:2 | 1.22 | 1.00 | 0.67 | 1.00 | 0.63 | 1.00 | 1.00 | 0.62 | 0.60 | 0.79 | 0.41 | 0.70 |
| PC aa C34:1 | 1.17 | 1.28 | 0.81 | 0.95 | 0.92 | 1.00 | 1.00 | 0.86 | 0.85 | 0.78 | 0.64 | 1.00 |
| PC aa C34:2 | 1.40 | 1.16 | 0.73 | 1.00 | 0.79 | 1.00 | 1.00 | 0.75 | 0.66 | 0.81 | 0.51 | 0.82 |
| PC aa C34:3 | 1.00 | 1.00 | 0.76 | 1.00 | 1.00 | 1.00 | 1.00 | 1.00 | 0.67 | 1.00 | 0.50 | 1.00 |
| PC aa C34:4 | 1.00 | 1.00 | 1.00 | 1.00 | 1.00 | 1.00 | 1.00 | 1.00 | 1.00 | 0.79 | 0.52 | 0.95 |
| PC aa C36:1 | 1.80 | 1.67 | 1.00 | 1.00 | 1.24 | 1.00 | 1.00 | 1.00 | 1.00 | 1.00 | 1.00 | 1.00 |
| PC aa C36:2 | 1.46 | 1.34 | 0.79 | 1.00 | 0.93 | 1.00 | 1.00 | 0.88 | 0.79 | 0.81 | 0.62 | 1.00 |
| PC aa C36:3 | 1.56 | 1.41 | 0.80 | 1.00 | 1.00 | 1.00 | 1.00 | 1.00 | 0.78 | 0.78 | 0.65 | 1.00 |
| PC aa C36:4 | 1.52 | 1.35 | 1.00 | 1.00 | 1.00 | 1.23 | 1.00 | 1.00 | 1.00 | 1.00 | 0.66 | 1.00 |
| PC aa C38:4 | 1.61 | 1.53 | 1.00 | 1.00 | 1.00 | 1.00 | 1.00 | 1.00 | 1.00 | 1.00 | 1.00 | 1.00 |
| PC aa C38:5 | 1.54 | 1.40 | 1.00 | 1.00 | 1.00 | 1.24 | 1.00 | 1.00 | 1.00 | 1.00 | 1.00 | 1.00 |
| PC aa C38:6 | 1.45 | 1.42 | 1.00 | 1.00 | 1.00 | 1.00 | 1.00 | 1.00 | 1.00 | 1.00 | 1.00 | 1.00 |
| PC aa C40:5 | 1.76 | 1.00 | 0.98 | 1.00 | 1.00 | 1.00 | 1.00 | 1.00 | 1.00 | 1.00 | 1.00 | 1.00 |
| PC aa C40:6 | 1.64 | 1.00 | 0.96 | 1.00 | 1.00 | 1.00 | 1.00 | 1.00 | 1.00 | 1.00 | 1.00 | 1.00 |
| PC ae C32:1 | 1.38 | 1.00 | 0.69 | 1.01 | 0.76 | 0.83 | 1.00 | 0.74 | 0.58 | 0.76 | 0.48 | 0.63 |
| PC ae C32:2 | 1.00 | 1.00 | 1.00 | 1.00 | 1.00 | 1.00 | 1.00 | 1.00 | 1.00 | 1.00 | 1.00 | 1.00 |
| PC ae C34:0 | 1.00 | 1.50 | 1.00 | 1.00 | 1.00 | 1.00 | 1.00 | 1.00 | 0.69 | 1.00 | 1.00 | 1.00 |
| PC ae C34:1 | 1.50 | 1.36 | 0.75 | 1.08 | 0.94 | 0.97 | 1.00 | 1.00 | 0.67 | 0.76 | 1.00 | 0.82 |
| PC ae C34:2 | 1.50 | 1.28 | 0.80 | 1.00 | 1.00 | 1.00 | 1.00 | 1.00 | 0.71 | 0.75 | 0.54 | 1.00 |
| PC ae C36:1 | 1.66 | 1.52 | 1.00 | 1.00 | 1.00 | 1.00 | 1.00 | 1.00 | 1.00 | 1.00 | 1.00 | 1.00 |
| PC ae C36:2 | 1.57 | 1.38 | 0.80 | 1.00 | 1.00 | 1.00 | 1.00 | 1.00 | 0.75 | 0.79 | 0.60 | 1.00 |
| PC ae C36:3 | 1.54 | 1.41 | 0.69 | 1.00 | 1.00 | 1.00 | 1.00 | 1.00 | 0.66 | 1.00 | 0.53 | 1.00 |
| PC ae C36:4 | 1.57 | 1.35 | 0.72 | 1.00 | 1.00 | 1.00 | 1.00 | 1.00 | 1.00 | 1.00 | 1.00 | 1.00 |
| PC ae C38:3 | 1.00 | 1.62 | 1.00 | 1.00 | 1.00 | 1.00 | 1.00 | 1.00 | 1.00 | 1.00 | 1.00 | 1.00 |
| PC ae C38:4 | 1.00 | 1.55 | 1.00 | 1.00 | 1.00 | 1.00 | 1.00 | 1.00 | 1.00 | 1.00 | 1.00 | 1.00 |
| PC ae C38:5 | 1.61 | 1.45 | 1.00 | 1.00 | 1.00 | 1.00 | 1.00 | 1.00 | 1.00 | 1.00 | 1.00 | 1.00 |

Value at 1.00 (grey shaded cell) indicates metabolite whose concentration ratio does not contribute to distinguish treated and untreated samples by OPLS-DA, s-plot multivariate analysis.
