# Supplementary material for: Different metabolic responses to PI3K inhibition in NSCLC cells harboring wild-type and G12C mutant KRAS
Source: Oncotarget. 2016 Jun 6;7(32):51462–72. doi: 10.18632/oncotarget.9849 (PMC5239488; doi:10.18632/oncotarget.9849)
Supplement: Supplementary file 3 [file oncotarget-07-51462-s003.docx]

Supplementary Table S5. List of metabolites determined using the Biocrates Absolute IDQ p180 kit.

| mETABOLITE CLASS | nUMBER | mETABOLITE NAME OR ABBREVIATION | Biological relevance  (SELECTED EXAMPLES) |
| --- | --- | --- | --- |
| Amino acids | 21 | Alanine, arginine, aspartate, citrulline, glutamine, glutamate, glycine, histidine, isoleucine, leucine, lysine, methionine, ornithine, phenylalanine, proline, serine, threonine, tryptophan, tyrosine, valine | Amino acid metabolism, urea cycle, activity of gluconeogenesis and glycolysis, insulin sensitivity, neurotransmitter metabolism, oxidative stress |
| Carnitine | 1 | C0 | Energy metabolism, fatty acid transport and mitochondrial fatty acid oxidation, ketosis, oxidative stress, mitochondrial membrane damage |
| Acylcarnitine | 39 | C2, C3, C3:1, C3-OH, C4, C4:1, C4-OH, C5, C5:1, C5:1-DC, C5-DC, C5-M-DC, C5-OH, C6, C6:1, C7-DC, C8, C9, C10, C10:1, C10:2, C12, C12-DC, C14, C14:1, C14:1-OH, C14:2, C14:2-OH, C16, C16:1, C16:1-OH, C16:2, C16:2-OH, C16-OH, C18, C18:1, C18:1-OH, C18:2 |  |
| Biogenic amines | 19 | Acetylornithine, asymmetric dimethylarginine, total dimethylarginine, alpha-aminoadipic acid, carnosine, creatinine, histamine, kynurenine, methionine sulfoxide, nitrotyrosine, hydroxyproline, phenylethylamine, putrescine, sarcosine, serotonin, spermidine, spermine, taurine | Neurological disorders, cell proliferation, cell cycle progression, DNA stability, oxidative stress |
| Lyso-phosphatidylcholines | 14 | lysoPC a C14:0/C16:0/C16:1/C17:0/C18:0/C18:1/C18:2/C20:3/C20:4/C26:0/C26:1/C28:0/C28:1 | Degradation of phospholipids, membrane damage, signaling cascades, fatty acid profile |
| Diacyl-phosphatidylcholines | 38 | PC aa C24:0/C26:0/C28:1/C30:0/C30:2/C32:0/C32:1/C32:2/C32:3/C34:1/C32:2/C34:3/C32:4/C36:0/C36:1/C36:2/C36:3/C36:4/C36:5/C36:6/C38:0/C38:1/C38:3/C38:4/C38:5/C38:6/C40:1/C40:2/C40:3/C40:4/C40:5/C40:6/C42:0/C42:1/C42:2/C42:4/C42:5/C42:6 | Dyslipidemia, membrane composition and damage, fatty acid profile, activity of desaturases |
| Acyl-alkyl-phosphatidylcholine | 38 | PC ae C30:0/C30:2/C32:1/C32:2/C34:0/C34:1/C34:2/C34:3/C36:0/C36:1/C36:2/C36:3/C36:4/C36:5/C38:0/C38:1/C38:2/C38:3/C38:4/C38:5/C38:6/C40:1/C40:2/C40:3/C40:4/C40:5/C40:6/C42:0/C42:1/C42:2/C42:3/C42:4/C42:5/C44:3/C44:4/C44:5/C44:6 |  |
| Sphingomyelins | 15 | SM (OH) C14:1, SM C16:0, SM C16:1, SM C16:1, SM C18:0, SM C18:1, SM C20:2, SM C22:3, SM (OH) C22:1, SM (OH) C22:2, SM C24:0, SM C24:1, SM (OH) C24:1, SM C26:0, SM C26:1 | Signaling cascades, membrane damage (eg. neurodegeneration) |
| Hexose | 1 | H1 | Carbohydrate metabolism |
| Total | 186 |  |  |
